# A structure-based framework for selective inhibitor design and optimization

**DOI:** 10.1038/s42003-025-07840-3

**Published:** 2025-03-12

**Authors:** Yurong Zou, Tao Guo, Zhiyuan Fu, Zhongning Guo, Weichen Bo, Dengjie Yan, Qiantao Wang, Jun Zeng, Dingguo Xu, Taijin Wang, Lijuan Chen

**Affiliations:** 1 State Key Laboratory of Biotherapy and Collaborative Innovation Center of Biotherapy, West China Hospital, Sichuan University, Chengdu, China; 2 Key Laboratory of Drug-Targeting and Drug Delivery System of the Education Ministry and Sichuan Province, West China School of Pharmacy, Sichuan University, Chengdu, China; 3 Western Health, Faculty of Medicine Dentistry and Health Sciences, University of Melbourne, Carlton, VIC Australia; 4 MOE Key Laboratory of Green Chemistry and Technology, College of Chemistry, Sichuan University, Chengdu, China; 5 Chengdu Zenitar Biomedical Technology Co., Ltd., Chengdu, China

**Keywords:** Computational models, Structure-based drug design

## Abstract

Structure-based drug design aims to create active compounds with favorable properties by analyzing target structures. Recently, deep generative models have facilitated structure-specific molecular generation. However, many methods are limited by inadequate pharmaceutical data, resulting in suboptimal molecular properties and unstable conformations. Additionally, these approaches often overlook binding pocket interactions and struggle with selective inhibitor design. To address these challenges, we developed a framework called Coarse-grained and Multi-dimensional Data-driven molecular generation (CMD-GEN). CMD-GEN bridges ligand-protein complexes with drug-like molecules by utilizing coarse-grained pharmacophore points sampled from diffusion model, enriching training data. Through a hierarchical architecture, it decomposes three-dimensional molecule generation within the pocket into pharmacophore point sampling, chemical structure generation, and conformation alignment, mitigating instability issues. CMD-GEN outperforms other methods in benchmark tests and controls drug-likeness effectively. Furthermore, CMD-GEN excels in cases across three synthetic lethal targets, and wet-lab validation with PARP1/2 inhibitors confirms its potential in selective inhibitor design.

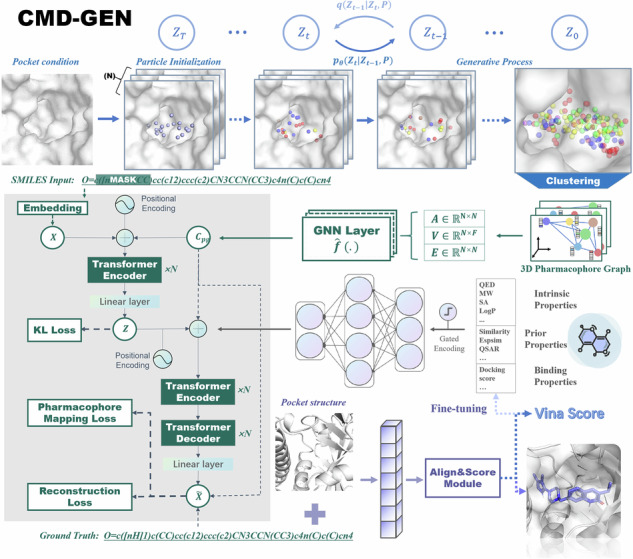

## Abstract (web summary)

CMD-GEN is a structure-based framework for selective inhibitor design, combining coarsegrained pharmacophore sampling with generative models to optimize molecular stability, druglikeness, and binding interactions in drug discovery.

## Introduction

The persistent quest for drug discovery stands as an enduring theme in the trajectory of human development. Relying solely on serendipitous drug discovery and empirical design proves insufficient for the demands of modern society. Within this context, the complex landscape of drug discovery highlights the significance of uncovering lead compounds^1^. Computational chemistry and bioinformatics have become the usual for lead compound rational design in recent decades^2–4^. Nevertheless, the intricate nature of biological systems poses a challenge in achieving precise simplification in physical models or empirical formulas, which could limit efficiency and result in false positives^5^. There is an urgent need for new technologies to accelerate the discovery of lead compounds and drive advances in drug development. The rise of artificial intelligence in recent years has breathed new vitality into the field of drug discovery^6^, particularly with models generated by deep learning technologies. These models learn from the different types of pharmaceutical data to make independent decisions for accomplishing specific objectives^7^. To a certain extent, they may be likened to the experience held by experts in the field of drug design. Hence, thoughtfully integrating artificial intelligence into the rational design and optimization of lead compounds holds promising potential.

In principle, the design of drug molecules revolves around their specific binding to target pockets, thereby influencing biological processes. Therefore, a pivotal consideration lies in the generation of molecules precisely tailored to bind to the cavity of these pockets^8^. In recent years, deep generative models have demonstrated impressive capabilities in addressing this challenge. Multiple studies suggest that effective molecular generation can greatly accelerate the identification of lead compounds^9–11^. The deep generative models for active molecule generation broadly divided into two categories: ligand-based and structure-based. Ligand-based models require learning compound space and fine-tuning the model using an active set of molecules^12^. However, the fundamental limitation is the inability to incorporate structural information of proteins (novel target family), hindering the generation of hits with unique binding patterns. In the field of structure-based generative models, some researchers have considered incorporating protein pockets as conditional into ligand-based model generation^13–15^. Others, taking a more ambitious approach, aiming to generate binding molecular conformations within the pocket. Models like LiGAN^16^, GraphBP^17^, and DiffSBDD^18^ aim to produce pocket-aware ligands with topology and three-dimensional (3D) geometry directly within the pocket. However, in tackling the complexity of molecular generation, the incorporation of conformational prediction introduces further intricacy. This may lead to molecular conformations that are non-optimal^19^, thereby failing to guarantee the biological activity in generated molecules. To tackle the aforementioned issues, some researchers have leveraged prior knowledge as generative conditions. These encompass molecular fragments^20,21^, pharmacophores^22^, interactions between molecules and targets^23^, and various molecular properties^24^. This approach steers the model towards approximating molecules with “biological activity” during the generation process.

However, The physical meaning of the molecular conformations produced under these conditions is unclear and they often deviate from the crystal conformation^19^. Moreover, these models may not handle more specialized design challenges well, such as generating dual-target inhibitors or highly selective inhibitors. The crux of the issue lies in the scarcity and substantial noise inherent in pharmaceutical data. Directly transferring the logic of text generation and image recognition, which has proven effective in other domains^25^, to drug design becomes problematic. Simultaneously, current models seem to be trained on the same and single dataset, limiting the potential of advanced algorithms, as shown in Table S1. Reflecting on the success of AlphaFold2^26^, its success stems from considering a co-evolutionary strategy and integrating multi-dimensional data. In the realm of pioneering drug design, it is crucial to incorporate the scientific concepts into AI, such as physical models, and leverage multi-dimensional data^27^. Therefore, devising a overarching architecture and workflow that goes beyond the algorithms themselves has become a key challenge for advancing the field of deep learning in drug discovery.

Considering the strengths and limitations of existing methodologies and drawing inspiration from coarse-grained molecular dynamics approaches, we introduce an innovative, structure-based 3D molecular generation framework. By decomposing the complex problem into sub-tasks, we transform molecules into pharmacophore point clouds and use pharmacophores as intermediaries, combined with diffusion model^28^ and transformer encoder-decoder, to establish associations between a finite number of 3D protein-ligand complex structures and a large number of drug molecule sequences. This hierarchical approach facilitates the incremental generation of molecules with potential biological activity. Concurrently, through iterative model training integrating molecular properties as conditions, molecular docking, and fine-tuning techniques, we achieve the generation of specific, active and drug-like molecules tailored for predefined target points. Leveraging pharmacophore point clouds, the generated molecular entities seamlessly align with target pockets, yielding physically meaningful three-dimensional molecules. Furthermore, by incorporating matching analysis of pharmacophore point clouds, Our model has performed capability in tasks such as generating selective inhibitors or dual-target inhibitors. Our approach complements the state-of-the-art structure-based molecular generation models in terms of model architecture. In this study, we present extensive experimental evidence, including wet-lab validations in designing highly effective PARP1/2 selective inhibitors, demonstrating that CMD-GEN is a powerful tool for addressing diverse drug design challenges and yielding practical outcomes. Our framework contributes to the existing drug discovery toolbox, providing new insights for both generation and prediction.

## Results and discussion

### Model architecture of CMD-GEN

**Fig. 1:**
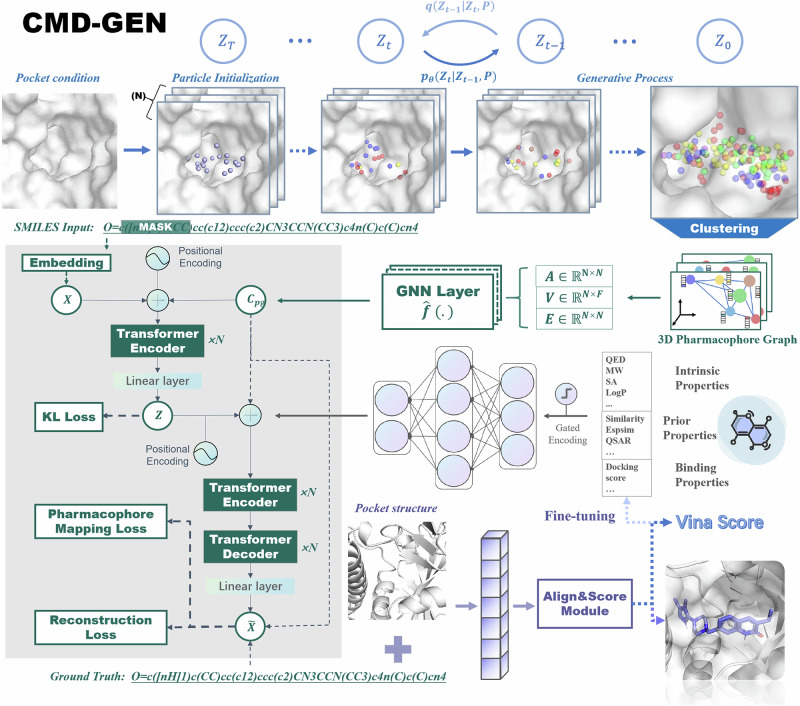
The overall architecture of CMD-GEN. It comprises a series of closely integrated modules: pocket-conditioned three-dimensional pharmacophore sampling module, space and feature-based gaussian mixture density clustering module, gating condition mechanism and pharmacophore-based molecular generation module and binding conformation generation module based on pharmacophore alignment.

The overall model architecture of CMD-GEN is shown in Fig. 1, which mainly includes a coarse-grained three-dimensional pharmacophore sampling module to the generation of coarse-grained ligand three-dimensional pharmacophore points under the constraint of protein pockets, a molecule generation module based on gating condition mechanism and pharmacophore constraints (GCPG) to convert the sampled important pharmacophore point cloud into a chemical structure, and a conformation prediction module based on pharmacophore alignment to align the pharmacophore point cloud sampled by diffphar with the chemical structure sampled by the GCPG module in three dimensions. The details of each module can be found in the methods section, and the training and generation process flowchart of the framework can be found in the Fig. S1.

### Performance of pocket-conditioned pharmacophore sampling

**Fig. 2:**
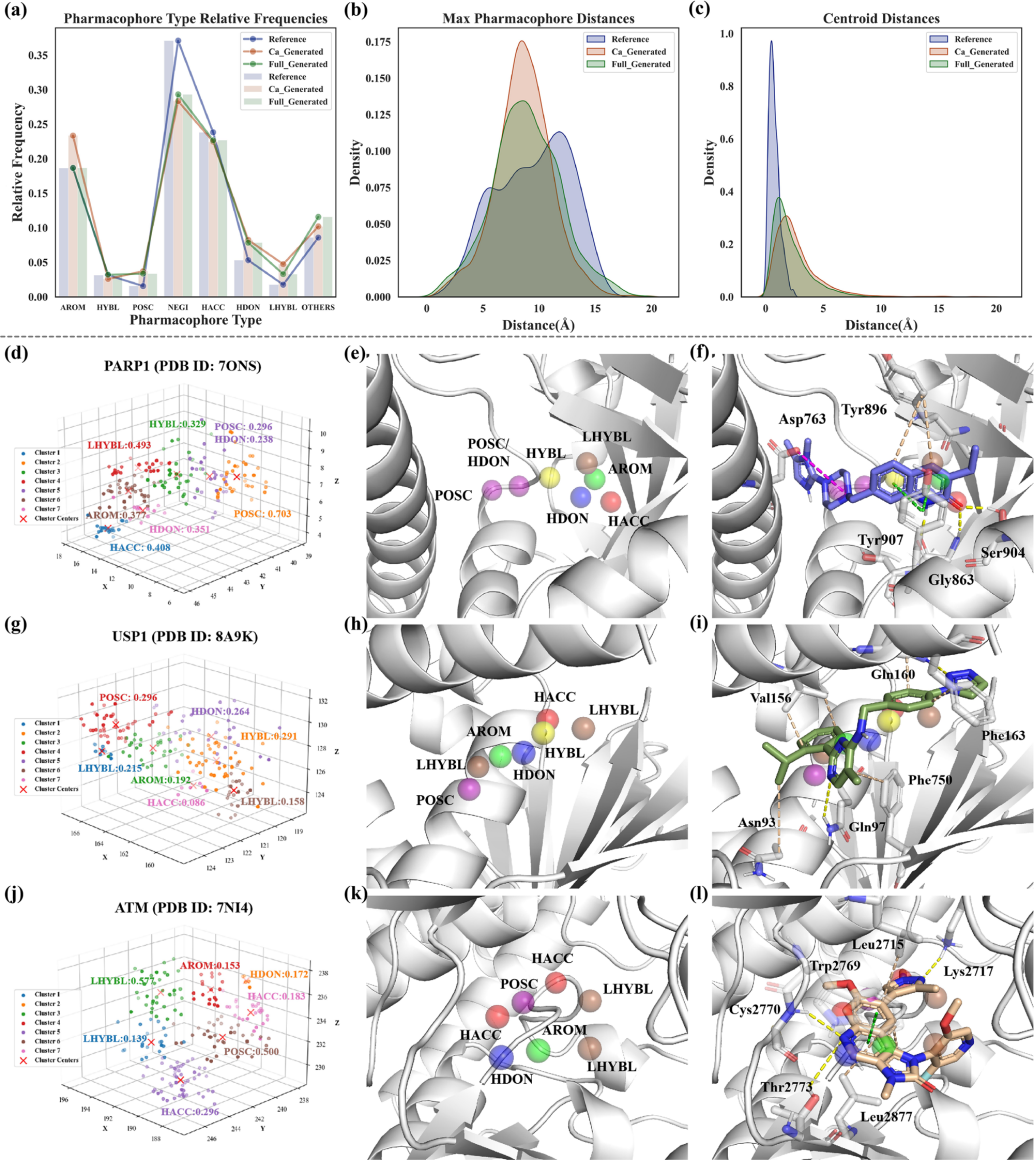
Evaluation and visualization of the pharmacophore sampling module. **a** The probability distribution of sampled pharmacophore types with respect to the original ligands. **b** Distributions of maximum distances between sampled and reference pharmacophores. **c** Distance Distribution between the centroids of sampled pharmacophores and reference pharmacophores. **d–f** Visualization of clustered sampled pharmacophore model within the PARP1 domain (PDB ID: 7ONS). The Figure also showcases the most probable pharmacophore types along with their corresponding frequency values. Additionally, a comparative visualization is provided with known active complexes in the pocket. **g–i** Visualization of clustered sampled pharmacophore model within the USP1 domain (PDB ID: 8A9K). **j–l** Visualization of clustered sampled pharmacophore model within the ATM domain (PDB ID: 7NI4).

We trained our module on the crossdocked dataset, analysing protein pocket descriptions using two methods: considering all atoms except hydrogen and focusing only on alpha carbon (Cα) atoms within residues. A comparison of the two models on the test set revealed distributions for pharmacophore types, maximum distances between features, and distances between pharmacophore centroids, as shown in Fig. 2.

Figure 2a demonstrates that the sampled distributions of three-dimensional pharmacophore types on the test set exhibit a good fit, regardless of whether the pocket residue representation is based on full atoms or Cα atoms. The distributions closely match those observed in the training set. The trained models exhibit matching distributions of maximum distances between pharmacophores, whether based on full-atom or Cα-atom representations, when compared with the original distances within the pockets (Fig. 2b). As shown in Fig. 2c, distances between the sampled pharmacophore point cloud centers and the original pharmacophore centers in complexes are compared, revealing that our model not only extends the spatial range of pharmacophore generation by learning the distribution within the pocket but also maintains proximity to the pocket without undue deviation.

In addition to validating the pharmacophore sampling module on the test set, we conducted analyses in real-world scenarios. Poly [ADP-ribose] polymerase 1 (PARP1)^29^ emerges as a crucial target in cancer therapy. Its inhibitors aim to disrupt the function of Poly(ADP-ribose) polymerase, utilizing a “synthetic lethality” mechanism for cancer cell treatment. Ubiquitin-specific protease 1 (USP1) is part of the Ubiquitin-specific protease family. ML323 is a selective inhibitor that likely interacts allosterically with USP1. In 2022, Rennie and Walden^30^ used cryo-electron microscopy to study the assembly of the USP1-ML323 enzyme-substrate-inhibitor complex. Additionally, the Ataxia telangiectasia mutated (ATM)^31^ target falls under the kinase category, and most of its inhibitors, including the clinical Phase I candidate M4076, bind to the kinase domain. We applied this model to these three targets of cancer drug development, which are PARP1 (PDB ID: 7ONS^29^), USP1 (PDB ID: 8A9K^30^), and ATM (PDB ID: 7NI4^32^), respectively. The sampled and visualized pharmacophore models are presented in Fig. 2d–l. The results reveal that the sampled pharmacophore features resemble the binding modes of ligands in the original crystal complexes. For instance, the pharmacophore model in Fig. 2d–f accurately captures the atomization, hydrogen bonding acceptor, and donor pharmacophore combination of the Isocarbostyril core. Simultaneously, the sampled PosIonizable pharmacophore and hydrophobic pharmacophore also fall within a reasonable spatial range. This underscores the significance of the pharmacophore sampling approach under the concept of virtual coarse-grained dynamics. Additionally, our approach enables rapid sampling of various combinations of pharmacophores and shows the pharmacophore models from five runs for each target, allowing generation modules to use them later (Supporting Information Table S2).

### Performance of GCPG module

Aligned with the aforementioned pharmacophore sampling module, we benchmark our designed GCPG module against alternative SMILES-based generation methodologies, encompassing ORGAN^33^, VAE^34^, SMILES LSTM^35^, Syntalinker^36^, and PGMG^22^. This comparative analysis serves to evaluate the performance of our model in molecular generation tasks. The assessment of molecular generation performance involves four key metrics: effectiveness, novelty, uniqueness, and the ratio of usable molecules. The comparison results are summarized in Table 1. We have trained the GCPG module and its other test models on the ChEMBL dataset^37^ based on the train-test split used in the GuacaMol dataset^38^. During testing, this module, relying on pharmacophore-conditioned constraints, generates molecules by approximating the distribution of conditioned compounds through random pharmacophore feature sampling. Simultaneously, a gating mechanism is employed to control molecular weight (MW) at 400, LogP at 3, QED at 0.6, and SA at 2, while closing the gating channels for RotaNumBonds and TPSA. This setup enables a comparison with the unconditional GCPG model, and the results are also collectively presented in Table 1.

**Table 1:** Performance of GCPG and other SMILES-based models

Methods	Validity$\uparrow$	Uniqueness$\uparrow$	Novelty$\uparrow$	Ratio of available molecules$\uparrow$
ORGAN	0.379	0.841	0.687	21.9%
VAE	0.870	0.999	0.974	84.7%
SMILES LSTM	0.959	**1.000**	0.912	87.5%
Syntalinker	**1.000**	0.880	0.903	79.5%
PGMG	0.982	0.979	0.976	93.8%
GCPG_noC_GT	0.992	0.973	0.970	93.5%
GCPG_noC_EGAT	0.989	0.986	0.977	95.3%
GCPG_noC_GINE	0.986	0.982	0.972	94.2%
GCPG_GatedGCN	0.980	0.997	0.969	94.8%
GCPG_EGAT	0.975	0.998	**0.983**	**95.6%**

An upward arrow next to each metric indicates that higher values represent better performance.

The best performance among all methods for each metric is shown in bold.

As shown in Table 1, the GCPG exhibits superior performance in novelty and the ratio of available molecules, particularly when leveraging EAGT to capture pharmacophore features. The GCPG_noC_EGAT module has yielded advancements surpassing the original PGMG across four key metrics, notably achieving a 1.5% increase in the ratio of available molecules. Furthermore, the comprehensive GCPG, integrating a gating condition mechanism, attains further refinements in generating novelty, uniqueness, and the ratio of available molecules. The available molecule ratio exceeds PGMG by approximately 1.8%, all while maintaining a comparable level of validity and uniqueness to other leading models, such as Syntalinker and SMILES LSTM. In contrast, incorporating EGAT for embedding pharmacophore features consistently yields superior results, potentially attributed to the significance of edge in pharmacophore graph within graph neural network, which can find some concurrence in recent literature^39^.

Moreover, we employed the match score to evaluate the model’s ability to generate molecules corresponding to pharmacophores, the results shown in Supporting Information Fig. S2. It indicates that the non-gating-condition “GCPG_noC_EGAT” achieved matching ratio of 78.25%, surpassing the 77.45% achieved by PGMG. Nevertheless, we observed a marginal decline in the matching ratio to 74.48% upon the introduction of the gating condition mechanism in GCPG. This decrease may be attributed to the heightened training complexity associated with the additional gating condition. Nevertheless, the marginal reduction in matching ratio brings about significant advantages. The supplementary gating condition enhances our ability to control parameters influencing molecule generation, and when combined with the fine-tuning method, it enables the optimization of computable property.

**Fig. 3:**
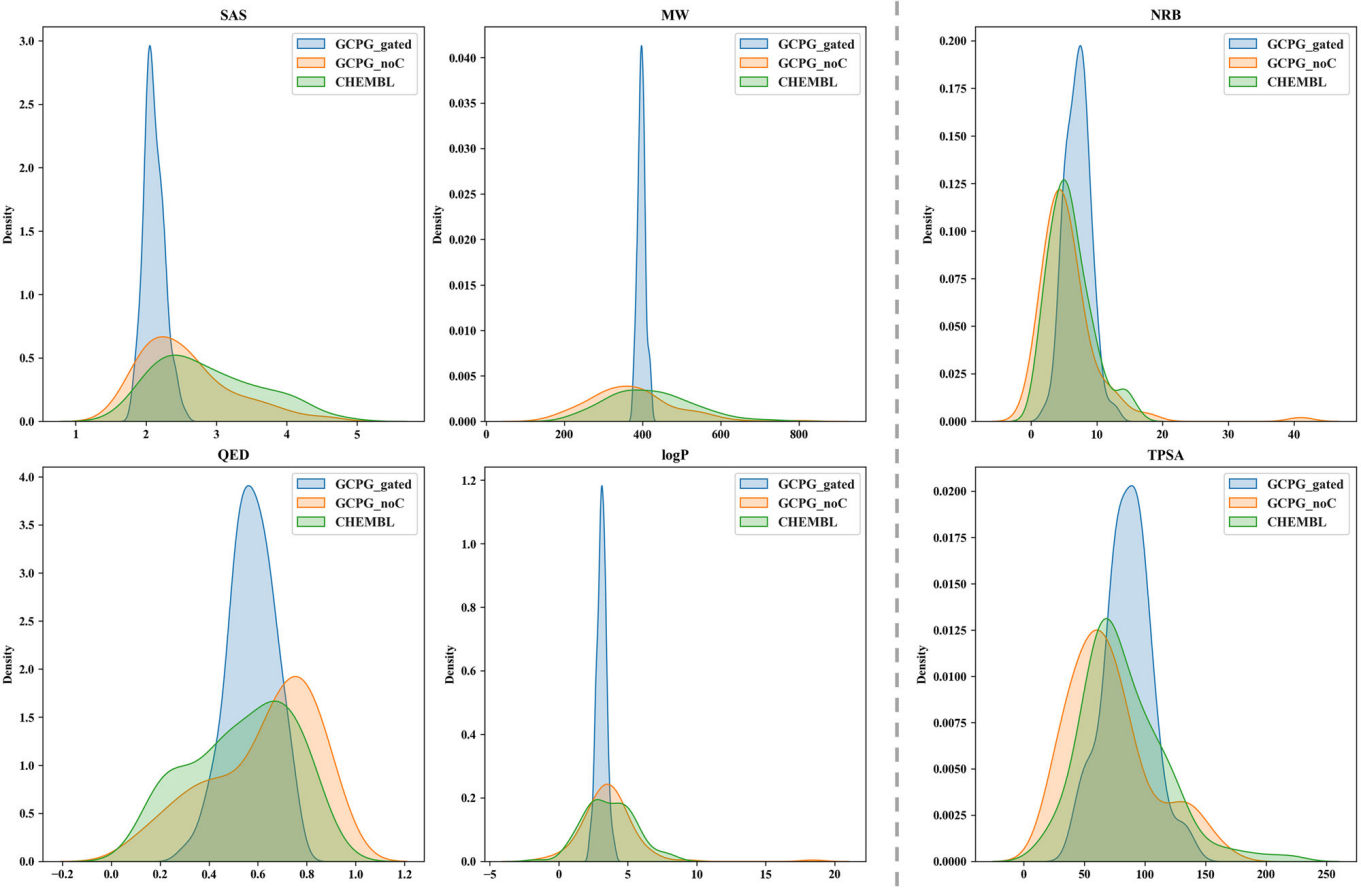
Distribution of the physicochemical properties for the ChEMBL training set and molecules generated by GCPG module with and without the gating condition mechanism. It Includes synthetic accessibility score (SAS, 0–10 range); Molecule weight (MW); number of rotatable bonds (NRB); quantitative estimate of druglikeness (QED), the Wildman–Crippen partition coefficient (LogP) and topological polar surface area (TPSA). The GCPG generated molecules include a total of 100,000 molecules from random pharmacophore hypotheses and the ChEMBL molecules comprise 100,000 molecules randomly sampled from the ChEMBL training datasets. In the gating condition mechanism, SAS is set to 2, MW is set to 400, QED is set to 0.6, and LogP is set to 3, with no restrictions on NRB and TPSA.

Figure 3 shows the comparison of the distribution of physicochemical properties between the ChEMBL training set and molecules generated by GCPG with and without the gating condition mechanism. It is evident that the GCPG with gating condition can adeptly reproduce the distribution of the ChEMBL training set (Fig. 3). In contrast, the GCPG module without the gating condition mechanism tends to generate molecules that align closely with the specified parameters, as evident in MW, SAS, QED, and LogP. Notably, properties unaffected by the gating channel, such as RotaNumBonds and TPSA, maintain distributions that closely resemble those of the ChEMBL training set. Those underscore the robust generative capabilities of our gating condition and pharmacophore-constrained module, showcasing not only its prowess in pharmacophore-based molecule generation but also our ability to control the properties of the generated molecules through parameter settings. Such multi-parameter control and optimization methods are highly beneficial for specific tasks, such as designing brain-permeable or low-toxicity molecules.

### Structure-based drug design for specific targets using CMD-GEN

The ultimate objective in the development of structure-based deep molecular generation models is their application to real systems. Here, we continue to focus on the three synthetically lethal targets, PARP1, USP1, and ATM, to explore the performance of the CMD-GEN model in real-world drug design projects within these specific targets.

As a comparative analysis, we focused on state-of-the-art (SOTA) models for structure-based molecular generation, including pocket2mol^40^, ResGen^41^, and Surfgen^19^. These models have previously demonstrated superior affinity score predictions compared to experimentally validated active molecules. Specifically, we conducted dedicated testing on the molecular generation results of CMD-GEN after fine-tuning the scoring values, solely controlling the scoring through a gating condition mechanism, referred to as CMD-GEN(R1). Detailed settings are provided in the Methods section. The version directly controlling physicochemical properties is termed CMD-GEN(R2). In each of the three target molecules, we sampled five pharmacophore models which can be find in Supporting Information Table S2. Based on these models, we generated 100 molecules for each, resulting in a total of 500 molecules. The remaining models, Pocket, ResGen, and Surfgen, underwent two rounds of generation, producing 250 molecules in each round and totaling 500 molecules.

**Fig. 4:**
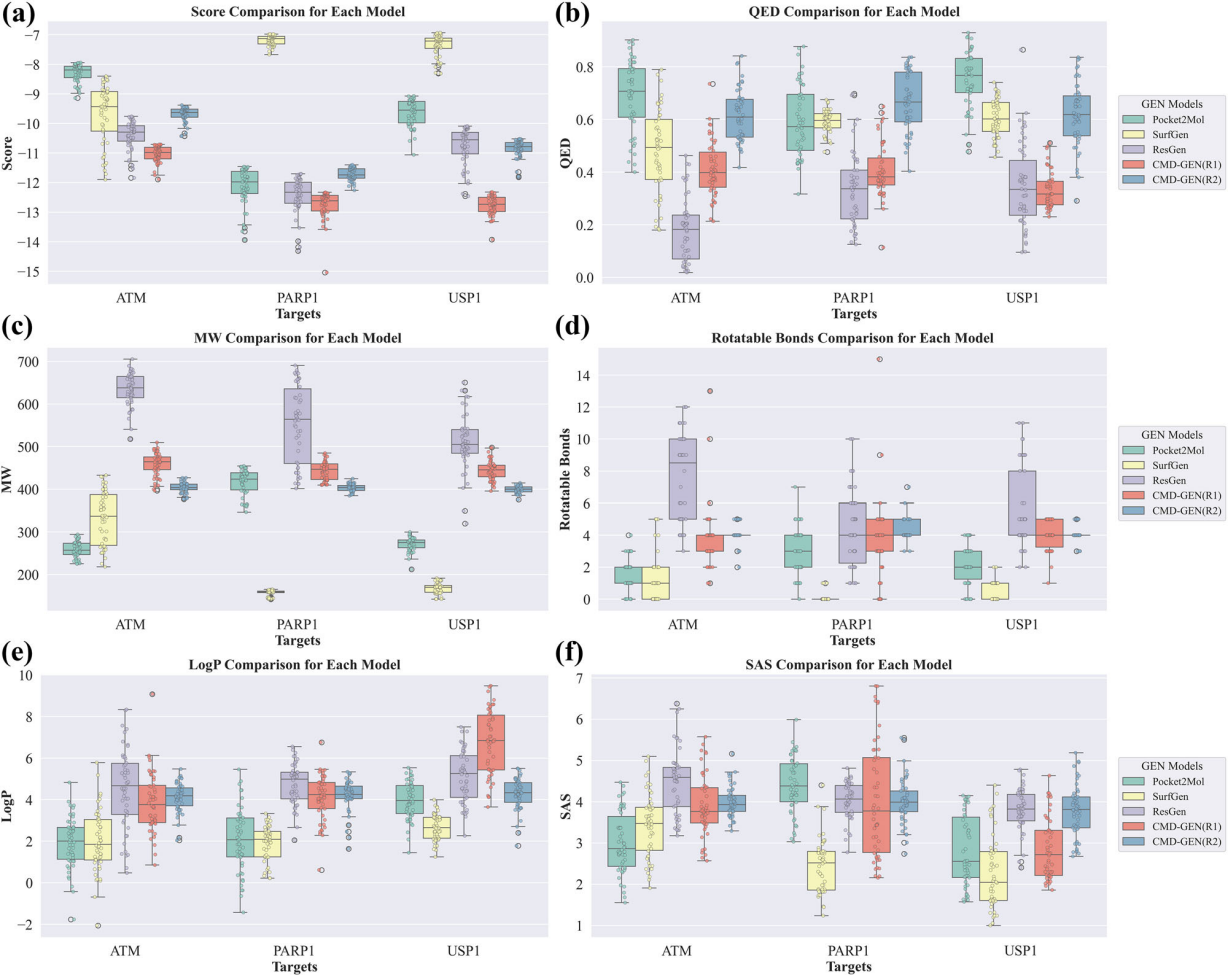
The boxplot of top 50 molecules for each model in three targets mean binding energies and drug-likeness properties. **a** The docking score ($\downarrow$) comparison. **b** The QED ($\uparrow$) comparison. **c** The MW comparison. **d** The rotatable bonds comparison. **e** The LogP comparison. **f** The SAscore ($\uparrow$) comparison. Calculation of drug-likeness properties uses molecular structures directly generated by the models. In the gating condition mechanism of CMD-GEN(R1), the docking score is fixed at −13 kcal/mol. For CMD-GEN(R2), the gating conditions include setting SAS to 4, MW to 400, QED to 0.6, LogP to 4, and rotatable bonds to 4.

We compared and analyzed the top 50 scored molecules from each model, presenting the results in Fig. 4. From the boxplot in Fig. 4a, it can be observed that the CMD-GEN(R2) model, governing the drug-like properties of gate molecules, achieves comparable scores across all three targets. It only slightly trails behind ResGen at the ATM and PARP1 targets. After fine-tuning the docking score through the gating mechanism, the CMD-GEN(R1) model surpasses all other models in the score distribution of generated molecules, showcasing the success of the fine-tuning strategy employed in CMD-GEN. As shown in Fig. 4b, the CMD-GEN(R2) model maintains a control value of 0.6 for QED. As a result, the QED scores of generated molecules consistently maintain a high level, with median values surpassing 0.6. Notably, the Pocket2mol model performs better in QED, maintaining high levels across all three targets. However, it displays instability in the weight of generated molecules, with big variations between the three targets, as shown in Fig. 4c. A similar trend is observed with SurfGen, which can only sample molecules with a molecular weight of around 100 on PARP1 and USP1 targets, showing relatively better performance on ATM. Resgen tends to produce compounds with larger molecular weights, averaging over 600 in the ATM target. Conversely, CMD-GEN(R1) and CMD-GEN(R2) exhibit a smaller distribution around 400, attributed to the presence of the gating mechanism. Furthermore, we are pleased to observe that the presence of the gating mechanism enables the CMD-GEN model to precisely control the number of rotatable bonds, a parameter reflecting molecular flexibility. The CMD-GEN(R2) model, without undergoing score fine-tuning, demonstrates superior control over parameters. Similar results are evident in the comparison of LogP in Fig. 4e and other drug-like properties. While the SAscore of all molecules in Fig. 4f remains favorable, it is important to note that the molecular conformation after docking from previous generative models significantly worsened, as depicted in Supporting Information Fig. S3. In comparing the conformations of directly generated molecules and after docking, we observed the emergence of multiple chiral centers, increasing synthetic challenges. This could be attributed to the generated molecular bond lengths and angles adopting unrealistic patterns^19,42^, causing the docking software to misinterpret them as chiral centers during the docking process. Contrarily, our CMD-GEN model avoids such issues due to the designed architecture. The initial conformation is directly generated based on RDKit^43^, merely aligning into the sampled coarse-grained pharmacophores.

**Fig. 5:**
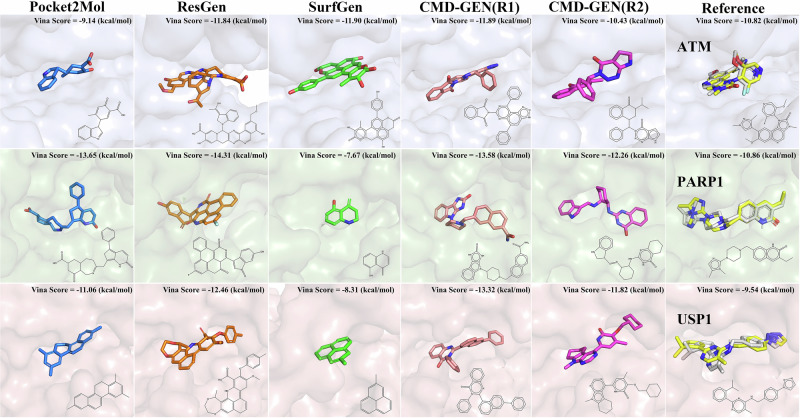
Docking conformations of the reference ligand and the top-scoring molecules in 500 sampled molecules from each model. Displayed with the blue background for the ATM, the green background for the PARP1, and the orange background for the USP1. Molecules from Pocket2mol are displayed in blue, Resgen in orange, surfgen in green, CMD-GEN(R1) in pink, and CMD-GEN(R2) in purple. Re-docking was conducted to validate the authenticity of the reference scoring. The docking conformations of reference molecules are highlighted in yellow, with their crystal structure conformations in white.

In Fig. 5, we present the highest-scoring molecular structures for each model and re-docked the original PDB, confirming their ability to replicate the native binding modes for these three targets. This affirms the reference value of the scoring results. Consistent with the above, molecular structures generated by Pocket2mol and SurfGen are relatively small, while those from ResGen are larger. In most cases, the scoring values of generated molecules can surpass those of the reference ligands. However, it is noteworthy that the highest-scoring molecules for these three models are predominantly multi-ring structures (four rings and above), with this tendency being particularly prominent in ResGen. Adding polycyclic structures is an easy way to increase docking scores. This strategy was probably used by these models, which favored the generation of polycyclic compounds. However, polycyclic aryl fused rings can easily cause safety issues and belong to a class of structural alerts^44–46^. In CMD-GEN(R1) and CMD-GEN(R2), the highest-scoring molecules do not adopt this strategy. Our gating mechanism limits the generation of excessively large molecules, and constraints on properties like QED and LogP provide implicit guidance, discouraging the blind generation of high-scoring molecules.

### Ablation experiment

**Fig. 6:**
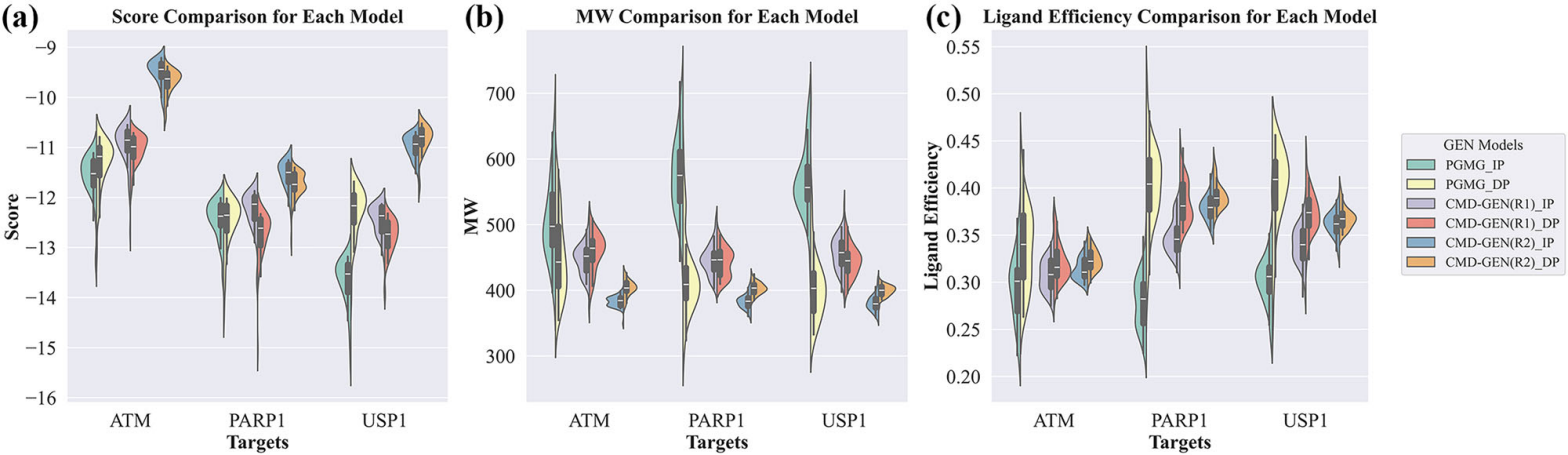
Violin plot of top 50 molecules generated using different pharmacophore models in three targets. **a** The docking score ($\downarrow$) comparison. **b** The MW comparison. **c** The ligand efficiency ($\uparrow$) comparison. “IP” refers to the interaction pharmacophore model of the reference complex, and “DP” refers to the pharmacophore model obtained by the pocket-conditioned pharmacophore sampling module of the CMD-GEN model.

We further conducted ablation experiments to confirm that the CMD-GEN model, after explicitly specifying pharmacophores, outperforms the PGMG model. Additionally, we demonstrated the capability of our pocket-conditioned pharmacophore sampling. The results are depicted in Fig. 6. Beyond PGMG, we observed that both CMD-GEN(R1) and CMD-GEN(R2) generally achieve better docking performance when utilizing their own sampled coarse-grained pharmacophores, as shown in Fig. 6a. In contrast, PGMG obtains superior scores when employing interaction-based pharmacophores, particularly in the ATM and PARP1 targets. Interestingly, across all three targets, PGMG generated molecules even outscore our fine-tuned docking model CMD-GEN(R1). To further investigate the reasons behind this result, we analyzed the molecular weights of generated molecules, as depicted in Fig. 6b. It becomes evident that PGMG tends to generate excessively large molecules. Molecules with MW greater than 700 were detected in the three targets. Additionally looked into ligand efficiency in docking, and the findings show that the CMD-GEN model has a better ligand efficiency distribution than the PGMG model. It can be concluded that larger molecules are typically produced in PGMG, which results in higher docking scores, because there is no gating condition mechanism in place. This emphasizes how crucial gating condition mechanisms are to realistic generative models of drug design. In addition, molecules generated by the diffuse pharmacophore of CMD-GEN generally showed higher ligand efficiency than those generated by known complex interaction pharmacophore models. This supports the validity of our coarse-grained pharmacophore sampling based on the diffusion model.

### CMD-GEN can generate physically meaningful binding conformations of molecules

**Fig. 7:**
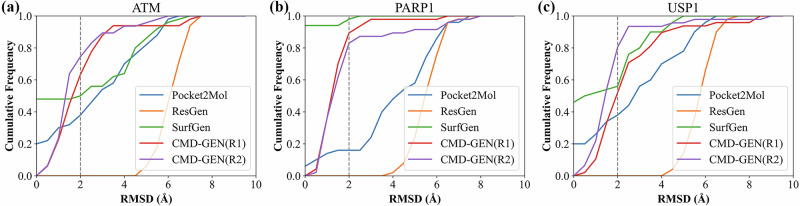
The root mean square deviation (RMSD) values of the generated conformations by the models and the docking conformations across all three targets. **a** RMSD comparison in ATM. **b** RMSD comparison in PARP1. **c** RMSD comparison in USP1.

In the context of CMD-GEN’s ability to generate high-quality molecules and adeptly avoid inaccuracies conformation in three-dimensional generative models regarding bond lengths and angles, we investigate the efficacy of CMD-GEN in generating molecular binding conformations. Given that the structural molecular generation models discussed above are trained on crossdocked docking structure datasets, it becomes interesting to explore whether the binding conformations of molecules generated by these models possess physical significance. Calculating the RMSD values of the overlapped conformations between the generated structures and the docking conformations for the aforementioned top 50 molecules, the results are depicted in Fig. 7. The SurfGen model exhibits excellent performance, particularly evident in PARP1, displaying nearly coincident RMSD values. The Pocket2Mol model demonstrates stable performance across all three targets, achieving favorable matching. However, the performance of ResGen is less satisfactory across all targets, primarily due to the larger molecular size and structural complexity, leading to suboptimal RMSD results. Additionally, its model architecture, based on a flow-based model, may contribute to differences between the generated molecules and the docking conformations. Alternatively, our CMD-GEN model samples the global pharmacophore and then aligns the molecules with the pharmacophore. It is evident that our CMD-GEN model, guided by this strategy, consistently performs well across all three targets. The conformations generated by the CMD-GEN (R2) model within less than 2 Å exceeded 75% on all three targets. This success is attributed to accurate pharmacophore sampling and the design of tolerance parameters (refer to the Methods description), allowing our molecule generation model to produce multiple possible binding conformations, in contrast to other models generating only a single conformation. The above illustrates that our molecular generation model can generate conformations with certain physical meaning.

### CMD-GEN model exhibits a fast molecular generation speed

The CMD-GEN model demonstrates a significant advantage in runtime due to its hierarchical design, where the final molecule generation relies on the global molecular structure decoded by Transformer, as shown in Supporting Information Table S3. In comparison to flow-based models like ResGen, our model achieves much higher efficiency. Calculations indicate that, after sampling pharmacophores, generating molecules, and aligning binding conformations, an average time of only 1.2 s is required for one molecule in CMD-GEN. In contrast, Resgen takes approximately 50–70 s. This rapid generation speed positions our model for widespread application in drug design processes, expediting drug development.

### CMD-GEN can be employed for the development of selective inhibitor molecules

Interestingly, due to the hierarchical design of the CMD-GEN framework, our model not only achieves the common task of generating three-dimensional molecules within a pocket given a structure, but also can dealing with more complex drug design scenarios, such as the development of selective inhibitors. In this process, the model integrates core pharmacophore features and ‘selective’ pharmacophore points to enhance selectivity, thus improving the specificity of the molecule-target interaction. Here, we take the development of selective inhibitors for PARP1 and PARP2 as an example. Both PARP1 and PARP2 share a significant homology in the C-terminal catalytic domain structure^47,48^. Currently, all approved PARP inhibitors (PARPi) are classified as ‘first-generation’ clinical PARPi, exhibiting non-selective binding and inhibitory effects on both PARP1 and PARP2^49^. However, it has been demonstrated that selectively inhibiting PARP1 alone is sufficient to induce cell death in homologous recombination-deficient cancer cells, while inhibiting PARP2 is associated with hematological toxicity^50^. Therefore, there is an urgent need for the development of selective inhibitors targeting PARP1.

**Fig. 8:**
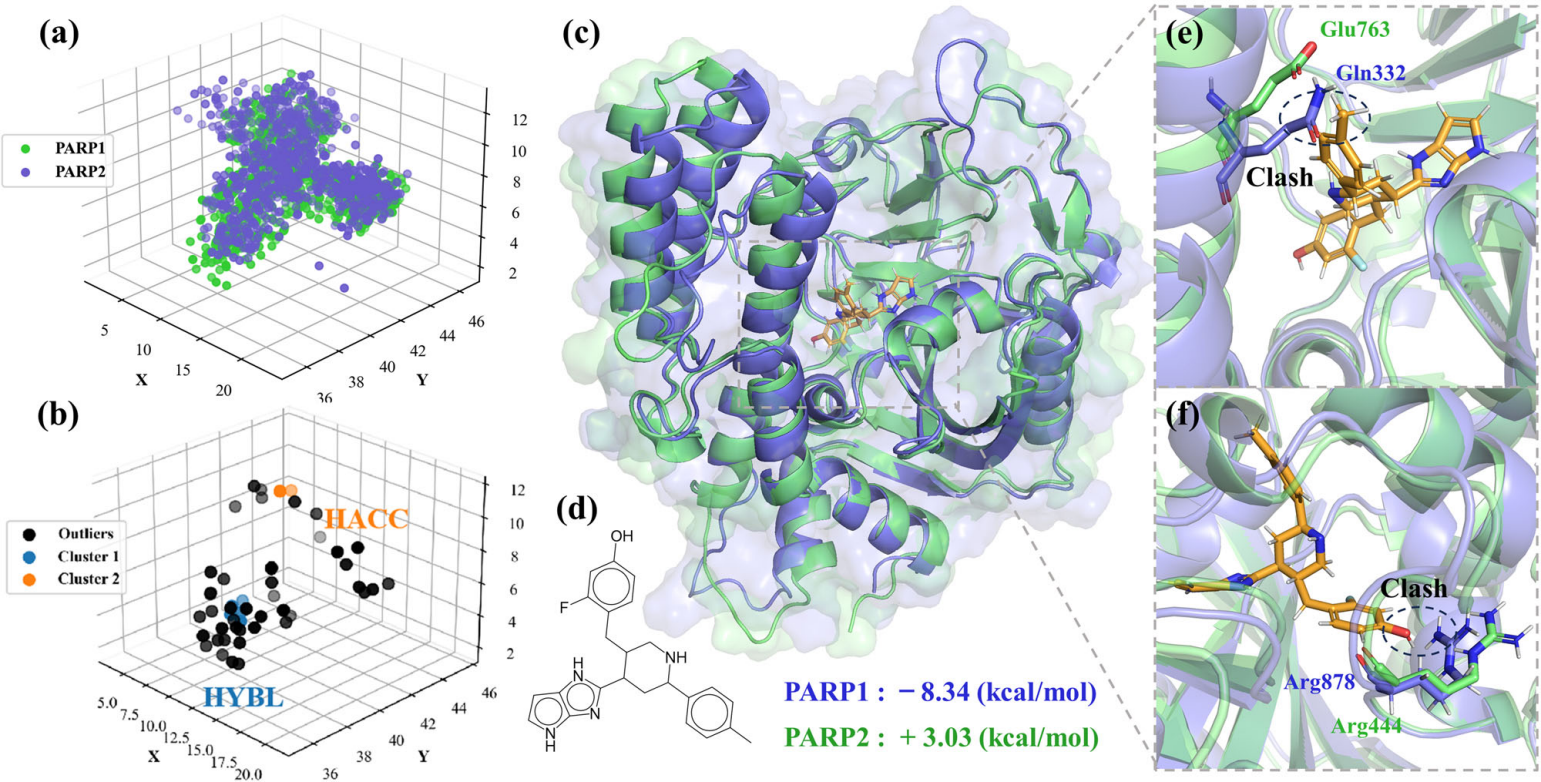
Illustration of the development of selective inhibitors based on the CMD-GEN model. **a** Matching pharmacophore point clouds. **b** Pharmacophore identification for selective features. **c** Binding landscape of the case molecule on the entire PARP1 (PDB ID: 7ONS) and PARP2 protein (PDB ID: 7R59^72^). **d** The chemical structure of case molecule. **e**, **f** Binding details of example ligand in the region of the selective pharmacophore.

In this task, point cloud representations of the coarse-grained pharmacophores from both pockets were aligned using a point cloud matching algorithm. A straightforward strategy considered points outside the geometric shape formed by the pharmacophore sampled for PARP1 compared to PARP2. The most probable pharmacophore features for these points were identified using a density-based DBSCAN^51^ clustering approach. Results are illustrated in Fig. 8a, b. In Fig. 8c, we showcase the binding mode of one compound generated by the CMD-GEN model with PARP1 and PARP2, while Fig. 8d displays its chemical structure. We observe the generation of a methyl group at the site of the selective hydrophobic pharmacophore in Fig. 8e. The structural presence of Gln332 in PARP2 conflicts with the methyl group in our molecule. Additionally, glutamine belongs to hydrophilic residues, substantiating the effectiveness of sampling the hydrophobic pharmacophore. This critical residue was similarly highlighted in studies by Hu et al. ^52^ and Wang et al. ^53^ when investigating the selective inhibition mechanism of NMS-P118 for PARP1/2. At another position, represented in Fig. 8f, corresponds to the sampled H-bond acceptor pharmacophore. We observe conflicts in terms of both charge and position with Arg878. In contrast, the different orientation of Arg444 in PARP1 avoids these conflicts while forming a hydrophobic pocket with Leu769^54^. Those confirms the validity of the sampled selective pharmacophore. We also employed a set of 100 molecules and assessed the in-situ scoring variations to ensure the results are not incidental, shown in Fig. S4. Over half had a gap of 2 kcal/mol or more, while PARP1-based configurations lacked selectivity.

Because the CMD-GEN model integrates the design advantages of pharmacophore models, it can also be adapted for multi-target drug design strategies, as shown in Fig. S5. Although CMD-GEN’s pharmacophores, derived from static crystal structures, can capture ‘selective’ conformations for semi-flexible docking tasks, it is important to consider that real-world target proteins are dynamic, often necessitating molecular dynamics simulations. Nevertheless, the strength of our model lies in its ability to translate residue differences into novel selective inhibitors.

### Real-World Applications of CMD-GEN in Developing PARP1/2 Selective Inhibitors

Given the limited wet-lab validation of generative models, we aim to further assess CMD-GEN’s potential in practical drug development. As mentioned above, we validated the CMD-GEN model across multiple experiments, highlighting its ability to generate potential inhibitors targeting specific sites. We also explored its capacity for selective inhibitor development. Continuing our focus on selective PARP1/2 inhibitors, we first investigated the selectivity mechanisms of AZD5305, a second-generation PARP1/2 inhibitor currently in Phase III clinical trials. AZD5305 underwent 1000 ns molecular dynamics simulations within the binding pockets of PARP1 and PARP2, followed by residue-based free energy decomposition, with results presented in Supplementary Fig. S6. Energy analysis revealed that AZD5305’s selectivity is primarily driven by a hydrophobic pocket formed by LEU769, ARG878, and PRO881 residues, which favor binding, whereas the analogous pocket is absent in PARP2 due to GLY338 and HIS447. This finding is consistent with CMD-GEN’s pharmacophore point cloud comparison for PARP1/2, as shown in Fig. 8a.

Based on the integrated analysis of CMD-GEN’s structure-based pharmacophore and the dynamic selectivity mechanisms of AZD5305, we set two development modes to assess the practical utility of the model. The first mode involves de novo design based on target structure, evaluating the model’s capability in generating novel molecules from structural information. The second mode employs a more conservative optimization strategy, retaining the AZD5305’s *N*-methyl-5-(piperazin-1-yl)picolinamide scaffold while generating other motifs of the molecule to assess the model’s optimization potential. Through a generation-and-screening process, 110 molecules were identified from 10,000 generated compounds using binding mode analysis and visual inspection (Fig. S7). Details are provided in the section “The setting of generation and screening process” in Supplementary Information. We have depicted all currently approved PARPi, including Olaparib, Rucaparib, Niraparib, and Talazoparib and a reported selective PARP1 inhibitors AZD5305. In addition, CMD-GEN successfully generated several “drug-likeness” structures consistent with these reported inhibitors (considering motifs with lactam ring systems), as shown in molecules 1–19. On this basis, the CMD-GEN model demonstrated its ability to generate more novel structures (molecule numbers 20–110), which were classified by us into 10 innovative strategies containing pharmacologized thinking, namely: single-ring strategy (lactam); single-ring strategy (isosterism); Double-ring Strategy (lactam); Double-ring Strategy (isosterism); Three-ring Strategy (lactam); Three-ring Strategy (isosterism); Four-ring Strategy; Ring-opening strategy; Macrocyclization strategy; and Other strategy. Subsequently, we considered to identify the most promising candidates among various strategies and practical factors such as patent rights and synthetic feasibility, selecting 12 molecules from two modes for molecular dynamics simulations to assess their binding stability, as illustrated in Table S4. For all selected molecules, we conducted 100 ns molecular dynamics simulations with both PARP1 and PARP2, with results shown in Figs. S8 and 9 and Table S5. The results demonstrated that all molecules remained stably bound within the pocket of PARP1 domain in the initial binding mode, as shown in Fig. S8. Free energy calculations revealed that molecules exhibited more favorable binding energies with PARP1 compared to PARP2. This indicates that CMD-GEN, by capturing receptor interaction pharmacophore patterns, generates molecules with a degree of dynamic stability and selective binding towards PARP1 from an energetic perspective.

We synthesized eight molecules from de novo design and optimization mode, as shown in Table 2. ELISA assays were performed to evaluate their biological activity against PARP1 and PARP2 at the protein level, as shown in Table 2 and Figs. S11 and 12. Of the eight synthesized molecules, six exhibited notable biological activity, demonstrating the model’s capability in structure-based inhibitor design and optimization. All molecules from the optimization mode displayed biological activity, with **Y5** achieving an IC50 of 12.7 nM, approaching drug-like levels. Additionally, both de novo designed and optimized molecules demonstrated selectivity, with **Y5** showing the highest selectivity ratio, exceeding 787-fold. These results validate our model’s ability to effectively distinguish protein pocket differences and integrate simulation and existing inhibitor knowledge, enhancing drug design efficiency.

**Table 2:** Biological activity and selectivity of PARP1-selective molecules generated by CMD-GEN

Entry	Structure	PARP1 Enzyme			PARP2 Enzyme			Selectivity PARP-2/1
		% control @ 0.01 μM^a^	% control @ 10 μM^a^	IC_50_ (nM)^b^	% control @ 0.01 μM^a^	% control @ 1 μM^*a*^	IC_50_ (nM)^b^	
Z2*		29.6	99.2	20.9	3.9	100.3	282.1	13.49
**Z5**		7.3	12.7	>10000	−0.8	1.1	>10000	
Z6*		16.3	62.8	6789	6.5	2.0	>10000	>1.47
Y1*		28.1	97.7	42.6	3.6	2.8	>10000	>234.74
**Y2**		−0.1	47.4	9800	2.3	−1.8	>10000	>1.02
**Y4**		20.7	99.0	86.5	−1.6	99.0	741.4	8.57
**Y5**		46.0	98.8	12.7	1.4	16.4	>10000	>787.4
**Y6**		70.5	99.1	6.0	87.6	101.3	2.4	0.4
**Olaparib**				0.7			1.4	

^a^% control, The inhibitory activity was evaluated at the compound.

^b^IC50, compound’s concentration required to inhibit PARP1/2 enzyme activity by 50%; To improve practical synthetic accessibility, some selected molecules were structurally simplified and annotated with * (Fig. S10).

Notably, the **Y6** molecule shows optimal binding activity, with an IC50 of 6 nM for PARP1, but exhibits even higher inhibitory activity against PARP2 (IC50 of 2.4 nM). We hypothesize that this lack of selectivity arises from structural sampling diverging to a critical pharmacophoric point beyond the established parameters. CMD-GEN pharmacophore sampling analysis identified key divergences between PARP1 and PARP2, particularly near GLU763 (GLN332), with Region 1 recognized as a promising site for PARP2 selectivity (see Fig. S13 and supported by selective PARP2 inhibitor UPF1069). Incorporating these insights, we performed ‘AROM’ sampling in this region during the generation process and get the R-series compounds (see Tables S4 and S6 and Fig. S14). The synthesized molecules **R1** and **R2** exhibited enhanced inhibitory activity against PARP2 relative to PARP1, with R1 showing an IC50 of 255 nM and a 13-fold selectivity for PARP2, underscoring the model’s potential in elucidating target selectivity mechanisms.

It must be acknowledged that the pharmacophore-based approach entails a degree of uncontrollability, as the number of initially defined pharmacophores is typically less than the total present in the molecule. Even when all set pharmacophores are satisfied, the model may still expand due to chemical structure sampling. In the future, we will consider more localized molecular optimization strategies, such as scaffold hopping and R-group substitutions, to more precisely control the generation and optimization of compounds.

## Discussion

Within the broader context of AI for Science, we believe that AI-contained domain knowledge is equally crucial for drug development. Undoubtedly, this approach will bring new solutions or perspectives to many challenging problems. Our study introduces the CMD-GEN framework, which connects 3D complexes with 2D drug-like molecule data through coarse-grained pharmacophore points. This model enriches generative training data with 2D drug-like molecules and 3D protein-ligand complexes information. The CMD-GEN framework decomposes the generation process of three-dimensional molecules within active pockets into three modules: the pocket-conditioned three-dimensional pharmacophore sampling module, the gating condition mechanism and pharmacophore-based molecular generation module, and the molecular binding conformation generation based on pharmacophore alignment. This approach effectively tackles the instability issue of molecular conformations that may arise in current generative models. Comprehensive analysis through benchmark experiments and real-world drug design tasks shows that the model preserves drug-like properties and produces meaningful binding conformations. Optimization of molecule properties is achieved via fine-tuning with our gating condition mechanism, accommodating multi-parameter constraints. The model surpasses other state-of-the-art methods in molecular generation efficiency and excels in complex drug design tasks, such as generating high-selectivity or multi-target inhibitors, facilitated by point cloud matching algorithms. Wet-lab experiments confirm the model’s effectiveness, exemplified by Y5, which shows over 787-fold selectivity for PARP1/2. This validates CMD-GEN’s capability to produce highly specific drug-like molecules and underscores its practical utility in drug development. CMD-GEN broadens the scope of drug design models, offering significant prospects for future research.

Although CMD-GEN performs optimally with static holo protein structures, static structures may exhibit inaccuracies due to resolution limitations, and many target proteins may only have apo conformations available. Future work will incorporate more domain knowledge in the CMD-GEN framework, e.g., by considering incorporating pocket dynamics. We believe that this framework, which integrates scientific principles with model design under conditions of limited pharmaceutical data, embodies the concept of ‘Science into AI’ and will offer new insights and opportunities for developing advanced AI-driven drug design models.

## Methods

### Dataset preparation

The training dataset for pocket-conditioned three-dimensional pharmacophore sampling module of CMD-GEN is derived from CrossDock2020^55^, a substantial collection of small molecules interacting with receptors. The initial assembly of this dataset comprises over 22 million protein-ligand pairs, clustered based on a 40% sequence similarity as per the original paper. Following the Pocket2mol^40^, conformations with an rmsd exceeding 2 Å are disregarded. While this reduces the dataset size, the screening ensures high-quality conformations, preserving the model’s ability to learn key pharmacophore features effectively.The training and testing datasets are separated to ensure a fair comparison in terms of model performance, with sequence similarity limited to less than 40%. Consequently, the remaining training set encompasses approximately 100,000 protein-ligand pairs, while the testing set comprises 100 protein pockets.

For the gating condition mechanism and pharmacophore-based molecular generation Module of CMD-GEN, we retrieved over 2 million unique SMILES (Simplified Molecular Input Line Entry System) format molecules from the ChEMBL 31 database^37^. To ensure data quality, preprocessing steps were implemented, including filtering molecules with a molecular weight exceeding 800, removal of small fragments and metals, and elimination of duplicate and invalid SMILES.

### Pocket-conditioned three-dimensional pharmacophore sampling module

The first segment of CMD-GEN is dedicated to the generation of coarse-grained ligand three-dimensional pharmacophore points under the constraint of protein pockets. Following previous work Hoogeboom et al. ^56^ and Schneuing et al.^18^, we employ an expanded equivariant Denoising Diffusion Probabilistic Model^57^ which inspired by non-equilibrium thermodynamics to sample latent three-dimensional coordinates of ligand pharmacophores within the pocket. In our settings, we provide protein pocket nodes ${{{{\mathcal{z}}}}}_{{data}}^{P}({{{{\mathcal{x}}}}}_{P},{{{{\mathcal{h}}}}}_{P})$ with 3D geometric coordinates ${{{{\mathcal{x}}}}}_{P}\in \,{{\mathbb{R}}}^{{{{{\rm{N}}}}}_{P}\times 3}$ categorical features ${{{{\mathcal{h}}}}}_{P}\in \,{{\mathbb{R}}}^{{{{{\rm{N}}}}}_{P}\times {{{{\rm{d}}}}}_{P}}$, where ${{{{\rm{N}}}}}_{P}$ is the number of protein pocket point cloud, as fixed three-dimensional context in each step of the denoising process and ${{{{\rm{d}}}}}_{P}$ corresponds to the type feature dimensions. This supplements the coarse-grained ligand pharmacophore point cloud ${{{{\mathcal{z}}}}}_{{data}}^{C}({{{{\mathcal{x}}}}}_{C},{{{{\mathcal{h}}}}}_{C})$ with 3D geometric coordinates ${{{{\mathcal{x}}}}}_{C}\in \,{{\mathbb{R}}}^{{{{{\rm{N}}}}}_{C}\times 3}$ pharmacophore categorical features ${{{{\mathcal{h}}}}}_{C}\,\in \,{{\mathbb{R}}}^{{{{{\rm{N}}}}}_{C}\times {{{{\rm{d}}}}}_{C}}$, refining the samples throughout the denoising process. ${{{{\rm{d}}}}}_{C}$ refers to the number of pharmacophoric categories. In this study, eight pharmacophoric categories were set: Aromatic, Hydrophobic, Localizable, Negativable, Acceptor, Donor, LumpedHydrophobe and Others. A fixed noise process add noise to the ${{{{\mathcal{z}}}}}_{{data}}^{C}$, yielding latent noisy ${{{{\mathcal{z}}}}}_{t}$ at each time step ${{{\rm{t}}}}$.

1 $$q({{{{\mathcal{z}}}}}_{t}|{{{{\mathcal{z}}}}}_{{data}}^{C})=N({{{{\mathcal{z}}}}}_{t}|{\alpha }_{t}{{{{\mathcal{z}}}}}_{{data}}^{C},{\sigma }_{t}^{2}{\rm I})$$

When employing a variance-preserving noising process with ${\alpha }_{t}=\sqrt{1-{\sigma }_{t}^{2}}$ ^58^, and considering the noising process to be Markovian, we can write the denoising transition from time step *t* to *s* < *t* in closed form as

2 $$q({{{{\mathcal{z}}}}}_{s}|{{{{\mathcal{z}}}}}_{{data}}^{C},{{{{\mathcal{z}}}}}_{t})={{{\mathcal{N}}}}\left({{{{\mathcal{z}}}}}_{s}|\frac{{\alpha }_{{t|s}}{\sigma }_{s}^{2}}{{\sigma }_{t}^{2}}{{{{\mathcal{z}}}}}_{t}+\frac{{\alpha }_{s}{\sigma }_{{t|s}}^{2}}{{\sigma }_{t}^{2}}{{{{\mathcal{z}}}}}_{{data}}^{C},\frac{{\sigma }_{{t|s}}^{2}{\sigma }_{s}^{2}}{{\sigma }_{t}^{2}}{\rm I}\right)$$

where ${\alpha }_{{t|s}}={\alpha }_{t}/{\alpha }_{s}$, and ${\sigma }_{{t|s}}={\sigma }_{t}/{\sigma }_{s}$. The true denoising process depends on the sample ${\hat{{{{\mathcal{z}}}}}}_{{data}}^{C}$. Instead, we reparameterize Eq. (1) and parameterize the noise predictor as indicated in Eq. (4) to directly predict Gaussian noise for obtaining approximation of the real samples, ${\hat{{{{\mathcal{z}}}}}}_{{data}}^{C}$. We parameterize the noise predictor with an E(n) Equivariant Graph Neural Networks (EGNN)^59^.

3 $${{{{\mathcal{z}}}}}_{t}={\alpha }_{t}{{{{\mathcal{z}}}}}_{{data}}^{C}+{\sigma }_{t}\epsilon ,\epsilon \in {{{\mathscr{N}}}}(0,{\rm I}),$$

4 $${\hat{\epsilon }}_{\theta }={\phi }_{\theta }\left({{{{\mathcal{z}}}}}_{t},{{{{\mathcal{z}}}}}_{{data}}^{P},t\right),$$

5 $${\hat{{{{\mathcal{z}}}}}}_{{data}}^{C}=\frac{1}{{\alpha }_{t}}{{{{\mathcal{z}}}}}_{t}-\frac{{\sigma }_{t}}{{\alpha }_{t}}{\hat{\epsilon }}_{\theta }$$

The neural network is trained to maximise the likelihood of observed data by optimizing a variational lower bound on the data, which is equivalent to the simplified training objective^60,61^ as ${{{{\mathcal{L}}}}}_{1}={\frac{1}{2}{{{\rm{||}}}}{{{\rm{\epsilon }}}}-{\phi }_{\theta }\left({{{{\mathcal{z}}}}}_{t},{{{{\mathcal{z}}}}}_{{data}}^{P},t\right){||}}^{2}$.

### Space and feature-based Gaussian mixture density clustering algorithm

To integrate with the coarse-grained pharmacophore sampling algorithm, we developed a Gaussian Mixture Models (GMM)-based algorithm^62^ for clustering pharmacophoric points in three-dimensional space. By combining spatial coordinates and feature information, the algorithm identifies the most probable pharmacophoric feature clusters within pharmacophoric point clouds obtained through multiple samplings. The algorithm begins with three-dimensional coordinate clustering using GMM. It then determines the most probable pharmacophoric feature within each cluster by jointly considering the overall feature probability and its likelihood within a specific cluster.

6 $${{Max}\_{prob}\_{feature}\_{for}\_{cluster}}_{i}={arg }{max }_{f}\left(\sum P({f|sample}\in {C}_{i})\right)$$

$P(f)$ represents the probability of feature $f$ in the overall pharmacophoric point cloud. $P({f|sample}\in {C}_{i})$ signifies the probability that a given data point has feature $f$ given that it belongs to cluster ${C}_{i}$.This concise yet effective approach allows us to capture the spatial distribution of pharmacophoric features while identifying the dominant features within distinct clusters.

### Gating condition mechanism and pharmacophore-based molecular generation module (GCPG)

Expanding on the methodologies implemented in the prior modules, using pharmacophores as a bridge, we have developed a sequence-based molecular generation module termed GCPG. This module integrates conditional gating and pharmacophore constraints to generate molecules with “high activity” and “high drug-likeness” for effective target binding. Following the approach of PGMG^22^, we have implemented a Transformer encoder-decoder architecture to handle the intricate many-to-many mappings between pharmacophores and molecules within the latent variable space. In order to further constrain the pharmacokinetics properties of the generated molecules, an additional gating mechanism has been incorporated into the model. This mechanism aims to optimize molecular properties or fine-tune any user-specified property, thereby enhancing the practical utility and efficiency of the molecule generation process.The form is as follows:

7 $$P({x|}{c}^{{phar}},{g}({c}^{{feat}}))=\int P({x|}{c}^{{phar}},{g}({c}^{{feat}}))P({z|}{c}^{{phar}},{g}({c}^{{feat}})){dz}$$

Where $g\left(\cdot \right)$ denotes the gating condition mechanism, applied at the latent state level to control how conditional embeddings and pharmacophore features influence the latent space $z$. Specifically, we have adopted a network framework based on the Transformer encoder-decoder^63^, where the encoder network, denoted as ${P}_{\phi }({z|}{c}^{{com}},x)$ to approximate $P({z|}{c}^{{com}})$ indirectly. Instead, the decoder network ${P}_{\phi }({x|}{c}^{{com}},z)$ approximate $P({x|}{c}^{{com}},z)$, where ${c}^{{com}}$ the combined condition vector between between${c}^{{phar}}$ and ${{{\rm{g}}}}({c}^{{feat}})$. In our configuration, we embed molecules in the SMILES format into dense feature vectors and utilize the EGAT^64^, a graph attention network, with the best-tested performance to embed pharmacophore features. We also tested other graph neural network layers such as Graph Transformer (GT)^65^ and GINE^66^.

The loss function in this module consists of three different terms, KL Loss, LM Loss, and the mapping loss. The first two terms are the negative evidence lower bound of the log likelihood log ${P}_{\theta }({x|}{c}^{{com}})$.

8 $${{{\mathcal{L}}}}_{2} = \underbrace{{-KL({P}_{\varphi }(z|{c}^{com},x)||P(z|{c}^{com}))}}_{KL \, Loss}+\underbrace{{\log {P}_{\theta }(x|z,{c}^{com})}}_{LM\,Loss}+Mapping \, Loss$$

Where ${{{\rm{KL\; Loss}}}}$ denotes the Kullback-Leibler divergence and we assume ${{{\rm{P}}}}({{{\rm{z|}}}}{{{{\rm{c}}}}}^{{{{\rm{com}}}}})$ the prior distribution of ${{{\rm{z}}}}$ follows a standard Gaussian ${{{\mathscr{N}}}}(0,{{{\rm{{I}}}}})$. The ${{{\rm{LM}}}}\; {{{\rm{Loss}}}}$ represents the reconstruction loss for language modeling, considering that x takes the form of a SMILES string. The mapping loss evaluates the model’s performance in predicting the mapping between the heavy atoms and pharmacophore elements, following the original configuration in PGMG. Importantly, it incorporates the use of the shortest path distance on the molecular graph represented by SMILES, as a substitute for the Euclidean distance assumed between two pharmacophore features^22^.

### Molecular binding conformation generation based on pharmacophore alignment

CMD-GEN aims to generate three-dimensional molecular binding conformations based on structure, utilizing a two-part approach involving pharmacophore sampling in the initial phase and property-constrained molecular generation in the second phase. Consequently, a straightforward overlay strategy is employed to attain the predicted binding conformations. In practical scenarios, the inherent stochastic errors in the model may prevent perfect alignment with pharmacophores. To address this, we introduce a tolerance parameter; for instance, a value of 1 indicates the model can tolerate one unmatched pharmacophore point in the current pharmacophore model. This flexible approach allows our generation model to potentially yield multiple computationally feasible ligand binding conformations, a capability not achievable by other structure-based generative models.

### Point cloud registration algorithm

Our hierarchically designed CMD-GEN is versatile, catering to specialized applications such as the generation of highly selective inhibitors for closely related targets and the generation of dual-target inhibitors. In both cases, the model can align the pharmacophores sampled for two targets by overlaying their point clouds^67^. In this study, we utilized the Kabsch 3D best-fit algorithm^68^ to maximize the alignment of pharmacophore point clouds from two pockets. For the generation of selectively inhibiting compounds, we consider the space sampled by the target pocket after overlaying, which was not sampled by the homologous target. This spatial pharmacophore point information facilitates the design of inhibitors with selective properties. In the development of dual-target inhibitors, the approach of pre-aligning point clouds followed by cluster sampling aids in facilitating a more comprehensive analysis of shared pharmacophore features for users.

### Evaluation

In the assessment of the pocket-conditioned three-dimensional pharmacophore sampling module, we conducted evaluations on a test set comprising 100 complexes. For each protein, the sampling involved a random number of pharmacophores. Evaluation metrics encompassed comparing sampled pharmacophore types’ probability distribution to the original ligands, analyzing maximum distance distributions between sampled and original ligand pharmacophores, and assessing centroid distance. These metrics collectively provide a comprehensive analysis of the model’s performance on the test set.

The gating condition mechanism and pharmacophore-based molecular generation module’s molecular outputs undergo assessment using four key metrics: validity, uniqueness, novelty, and the ratio of available molecules. Validity measures the percentage of chemically valid molecules, while uniqueness evaluates non-repetition among valid molecules. Novelty gauges the percentage of chemically valid molecules not found in the training set, and the ratio of available molecules indicates the proportion of novel molecules in all generated results. Additionally, a match score^22^ is employed to assess the alignment between generated molecules and a specified pharmacophore.

AutoDock Vina^69^ docking scores proxy the binding activity of generated molecules to target. We conduct semiflexible docking using AutoDock Vina with default parameters, considering ligand flexibility against a rigid receptor. Additionally, we employ widely-used metrics^70^ to assess the quality of our generated molecules: QED (Quantitative Estimate of Druglikeness), MW (molecular weight), SAS (synthetic accessibility score), logP (the Wildman–Crippen partition coefficient), RotaNumBonds (number of rotatable bonds), and TPSA (topological polar surface area) are employed as standard metrics to gauge the drug-likeness, molecular weight, synthetic accessibility, lipophilicity, flexibility, and polar surface area of the generated molecules, respectively. In the case of Structure-Based Drug Design for Specific Targets using CMD-GEN, the PDB structures used are ATM (7NI4), PARP1 (7ONS), and USP1 (8A9K). The CMD-GEN (R2) model employs five parameters, including MW, LogP, QED, SAS, and the number of rotatable bonds, as gating conditions. For fine-tuning the scoring model CMD-GEN (R1), molecular generation is conducted by controlling parameter ranges: MW (325–425), LogP (1–4), QED (0.4–0.8), SAS (1–5), and the number of rotatable bonds (4–6). Subsequently, docking scores are obtained for the top 50,000 generated molecules. These scoring values serve as gating conditions for fine-tuning the model, wherein only the scoring parameter is considered during the fine-tuning training process.

Concerning the generation of three-dimensional conformations, given the model’s training on a dataset rich in complex docking information, we compute the Root Mean Square Deviation (RMSD) between the generated ligand conformations and the highest-scoring conformation from the docking results. This serves as an indicator of the meaningfulness of the conformational generation process in capturing physical relevance. for the context of designing selective inhibitors, where considerations extend to molecular distinctions within two distinct pockets, we introduce an in-situ scoring approach based on AutoDock Vina to directly assess the affinity of generated conformations within homologous proteins.

### Statistics and reproducibility

In CMD-GEN, the pocket-conditioned 3D pharmacophore sampling module runs 500 diffusion steps with gradient clipping, using the Adam1 optimizer (learning rate 1e–4, batch size 16). The EGNN for noise prediction has a 6 Å cutoff, 256 dimensions, and 5 stacked layers. Training spans 1000 epochs, with “Full-atom” and “Cα-atom” representations showing optimal performance at epochs 281 and 472, respectively. The sampling process is repeated 5 times to ensure it is not a random event. The pharmacophore-based molecular generation module uses noise injection via an infilling scheme and a 384 hidden dimension with 8 transformer blocks. The Adam optimizer (learning rate 3e–4, weight decay 1e–6) is used, with cyclic cosine annealing every 4 epochs. Training lasts 32 epochs on a setup with 10 Intel Xeon Gold 6240R CPUs and 1 NVIDIA A100 GPU. The sampling process for this module also samples 100 molecules per iteration and is repeated 5 times. Data and code are provided for reproducibility purposes.

### Supporting information

Training Details; The Setting of Generation and Screening Process; Application of CMD-GEN in Developing PARP2 Selective Inhibitors; Fig. S1. Comparison of the Density Distribution Plots for Match Score among Models Conditioned on Pharmacophore; Fig. S2. Boxplot Comparison of SAscore After Docking Between Structure-based Molecular Generation Models; Fig. S3. The Comparison of Docking Score Gap from Generated 100 Molecules with Selectively Incorporated Pharmacophores; Fig. S4. Illustration of The Generation of Dual-target Molecules Based on the CMD-GEN model; Fig. S5. Root Mean Square Deviation (RMSD) of the PARP1 Domain in Complex with AZD5305 Over Simulation Time and The Corresponding MM/GBSA Binding Free Energy Decomposition Per Residue; Fig. S6. The Molecules Generated by The CMD-GEN Model were Screened to Identify 110 Potential PARP1 Inhibitors, Categorized According to Pharmacochemical Strategies; Fig. S7. RMSD of PARP1 and PARP2 Domains in Complex with Selected PARP1 Selective Inhibitor Molecules over the Simulation Time; Fig. S8. RMSD of PARP1 and PARP2 Domains in Complex with Selected PARP2 Selective Inhibitor Molecules over the Simulation Time; Fig. S9. Structural Simplification Strategy; Fig. S10. Binding Modes and IC50 Values of The Z-Series Compounds with PARP1 and PARP2; Fig. S11. Binding Modes and IC50 Values of The Y-Series Compounds with PARP1 and PARP2; Fig. S12. Pharmacophore Point Cloud Sampling and Docking Conformations of The Selective PARP2 Inhibitor UPF1069 in PARP1 and PARP2; Fig. S13. Binding Modes and IC50 Values of The R-Series Compounds with PARP1 and PARP2; Fig. S14. Illustrates Key Components and Performance Metrics of The Graph Neural Network Prediction Model; Table S1. The Training Data for Structure-based Three-dimensional Molecular Generation Models; Table S2. Five Pharmacophore Models Dampled and Clustered for Each of the Three Synthetic Lethality Targets, along with Pharmacophore Models for The Receptor-Ligand Complex Obtained through Schrödinger’s Develop Pharmacophore Model; Table S3. The Comparison of Runtime between Models; Table S4. PARP1/2 Selective Inhibitors Identified Using a Generate-Screen Strategy Within De Novo Design and Optimization Development Modes; Table S5. Binding Free Energies of All Selected Molecules with PARP1 and PARP2; Table S6. Biological Activity and Selectivity of PARP2-Selective Molecules Generated by CMD-GEN; Synthetic method; Biological Assay Methods; 1H NMR and 13C NMR Spectra of All Compounds. Supplementary Data 1 shows the source data behind the graphs in the paper.

### Reporting summary

Further information on research design is available in the Nature Portfolio Reporting Summary linked to this article.

## Supplementary information

Reporting Summary

Supplementary Data 1

Description of Additional Supplementary Materials

Supplementary Information

## Data Availability

The source dataset used to train and evaluate the overall model is provided at Zenodo^71^. Source Data for Figs. 2–4 and Figs. 6 and 7 are provided in Supplementary Data 1. For the drug discovery case studies, the PDB and ligand files of 7ONS, 8A9K, 7NI4, and 7R59 are downloaded from RCSB Protein Data Bank.

## Code availability

The source code of CMD-GEN is available at https://github.com/zyrlia1018/CMD-GEN. We also deposit the data and codes of CMD-GEN in Zenodo with DOI^71^.
