# Supplementary material for: A structure-based framework for selective inhibitor design and optimization
Source: Commun Biol. 2025 Mar 12;8:422. doi: 10.1038/s42003-025-07840-3 (PMC11903766; doi:10.1038/s42003-025-07840-3)
Supplement: Supplementary file 4 — Supplementary Information [file 42003_2025_7840_MOESM4_ESM.pdf]

Supporting Information

A Structure-Based Framework for Selective Inhibitor Design and Optimization

Yurong Zou[1,§], Tao Guo[1,§], Zhiyuan Fu[1,§], Zhongning Guo[1], Weichen Bo[1], Dengjie Yan[3], Qiantao Wang[3], Jun Zeng[4], Dingguo Xu[5], Taijin Wang[2,*] and Lijuan Chen[1,2,*]

[1]State Key Laboratory of Biotherapy and Collaborative Innovation Center of Biotherapy, West China Hospital, Sichuan University, Chengdu, 610041, China

[2]Chengdu Zenitar Biomedical Technology Co., Ltd., Chengdu, 610045, China

[3]Key Laboratory of Drug-Targeting and Drug Delivery System of the Education Ministry and Sichuan Province, West China School of Pharmacy, Sichuan University, Chengdu, 610041, China

[4]Western health, Faculty of medicine, dentistry and health sciences. University of Melbourne. Carlton, VIC 3010, Australia

[5]MOE Key Laboratory of Green Chemistry and Technology, College of Chemistry, Sichuan University, Chengdu, 610064, China

[§]These authors contributed equally and should be considered as co-first authors

*Corresponding authors. Cancer Center, West China Medical School, West China Hospital, Sichuan University, Chengdu 610041, PR China.

E-mail addresses: chenlijuan125@163.com.

Supporting Information

List of Contents

Training details S4

The setting of generation and screening process S6

Application of CMD-GEN in Developing PARP2 Selective Inhibitors S7

Figure S1. Comparison of the density distribution plots for match score among models conditioned on pharmacophore. S10

Figure S2. Comparison of the density distribution plots for match score among models conditioned on pharmacophore. S11

Figure S3. Boxplot comparison of SAscore after docking between structure-based molecular generation models. S12

Figure S4. The Comparison of docking score gap from generated 100 molecules with selectively incorporated pharmacophores. S13

Figure S5. Illustration of the generation of dual-target molecules based on the CMD-GEN model. S14

Figure S6. Root mean square deviation (RMSD) of the PARP1 domain in complex with AZD5305 over simulation time and the corresponding MM/GBSA binding free energy decomposition per residue. S15

Figure S7. The molecules generated by the CMD-GEN model were screened to identify 110 potential PARP1 inhibitors, categorized according to pharmacochemical strategies. S16

Figure S8. Root mean square deviation (RMSD) of PARP1 and PARP2 domains in complex with selected PARP1 selective inhibitor molecules over the simulation time. S18

Figure S9. Root mean square deviation (RMSD) of PARP1 and PARP2 domains in complex with selected PARP2 selective inhibitor molecules over the simulation time. S19

Figure S10. Structural Simplification Strategy. S20

Figure S11. Binding modes and IC50 values of the Z-Series compounds with PARP1 and PARP2. S21

Figure S12. Binding modes and IC50 values of the Y-Series compounds with PARP1 and PARP2.

S22

Figure S13. Pharmacophore point cloud sampling and docking conformations of the selective PARP2 inhibitor UPF1069 in PARP1 and PARP2. S23

Figure S14. Binding modes and IC50 values of the R-Series compounds with PARP1 and PARP2. S24

Figure S15. Illustrates key components and performance metrics of the graph neural network prediction model. S25

Table S1. The training data for structure-based three-dimensional molecular generation models. S26

Table S2. Five pharmacophore models sampled and clustered for each of the three synthetic lethality targets, along with pharmacophore models for the receptor-ligand complex obtained through Schrödinger's Develop Pharmacophore Model. S27

Table S3. The comparison of runtime between models. S32

Table S4. PARP1/2 selective inhibitors identified using a generate-screen strategy within de novo design and optimization development modes. S33

Table S5. Binding free energies of all selected molecules with PARP1 and PARP2. S34

Table S6. Biological Activity and Selectivity of PARP2-Selective Molecules Generated by CMD-GEN. S36

Synthetic method S37

Biological Assay Methods S53

^{1}H NMR and ^{13}C NMR spectra of all compounds S55

Training details

For the pocket-conditioned three-dimensional pharmacophore sampling module, the diffusion process is executed 500 times. Gradient clipping technique is applied during training, utilizing the Adam[1] optimizer with a learning rate of 1e–4 and a batch size of 16. In the denoising process. The EGNN employed for noise prediction is configured with a 6 Å cutoff to mitigate model training expenses. The dimensions are set to 256, and this layer is iteratively stacked five times to extract ligand coordinates and features following each denoising step. The overall training consists of 1000 epochs. Model parameters for both "Full-atom" and "Ca-atom" representations of protein pockets remain consistent. The model with the "Full-atom" representation achieves optimal performance at the 281st epoch, whereas the model with the "Ca-atom" representation attains its best performance at the 472nd epoch.

Within the framework of the gating condition mechanism and pharmacophore-based molecular generation module, we enhance training robustness by injecting noise through an infilling scheme. Random subsequences within input sequences undergo substitution with a [mask] token. The hidden dimension is set at 384, featuring the integration of eight stacked transformer blocks, both in the encoder and decoder domains. The attention mechanism employs eight heads, and the feed-forward dimension is configured at 1024. Optimization strategies encompass the utilization of an Adam optimizer with a 3e–4 learning rate and a 1e–6 weight decay. A cosine learning rate annealing strategy is implemented cyclically every four epochs.

Gradient clipping is applied, capping the maximum gradient at five. The training process spans 32 epochs. the computational backbone for the training regimen is grounded in the formidable synergy of 10 Intel(R) Xeon(R) Gold 6240R CPUs @ 2.40GHz and a singular NVIDIA A100.

The setting of generation and screening process

In the *de novo* generation of PARP1/2 inhibitors using the CMD-GEN model, we first generated approximately 490,000 molecules from a sampled pharmacophore model with selective pharmacophore points and subjected them to docking scores. Molecular weight (MW) ranged from 375-415 with a step size of 20; LogP from 1 to 4 with a step size of 1; QED from 0.5 to 0.7 with a step size of 0.1; and synthetic accessibility scores (SAS) from 2.5 to 3.5 with a step size of 0.5. The number of rotatable bonds was set at 2 to 5, with docking scores ranging from -13 to -10. These molecules and their docking scores were used to systematically pre-train and fine-tune the CMD-GEN model with a learning rate of 10e-8. We then employed a graph neural network to map SMILES to docking scores, as shown in Fig. S15, refining the model through three rounds of scoring and fine-tuning. The final model generated 10,000 molecules, from which candidates were selected based on pharmacophore matching scores, predicted score and visual inspection, resulting in a total of 110 molecules representing diverse design strategies, as shown in Fig. S7. We found that some generated molecules can exhibit local scaffold similarity to the marketed compounds and AD5305 within the lactam-binding domain.

Furthermore, the model demonstrates robust drug design capabilities. In the single-ring strategy, molecules incorporating a lactam structure encompass five-membered and six-membered rings, as well as aromatic and saturated rings, and extend to other structures based on the concept of isosterism (utilizing atomified

nitrogen or carbonyl groups as hydrogen bond acceptors). Within the double-ring strategy for the lactam binding domain, the model diversifies pharmacochemical approaches by adjusting the size and position of the fused rings, conducting ring design around the lactam-containing ring, and employing amino or hydroxyl groups as hydrogen bond donors in the isosteric components of the dual rings. For the design of three-ring inhibitors, the model innovates upon a dual-ring framework; for instance, molecule 100 utilizes a cyano group as a hydrogen bond acceptor and exhibits strong binding affinity within the pocket. Additionally, the design strategies for four-ring and open-ring inhibitors, as well as macrocyclic inhibitors, were explored. Notably, CMD-GEN also attempted to use bridged rings as linkers connecting the lactam moiety. This binding mode is challenging to identify within two-dimensional structures, yet CMD-GEN, leveraging its three-dimensional insights, generates novel molecules with similar conformations.In summary, the CMD-GEN model adeptly produces binding fragments analogous to known molecules based solely on structural data, and demonstrates a thought process akin to that of experienced medicinal chemists.

For the development of novel PARP1/2 inhibitors based on optimizing AZD5305, we retained the N-methyl-5-(piperazin-1-yl)picolinamide motif as a guiding principle, informed by molecular dynamics studies and existing literature. Fragmentation and recombination processes yielded a series of molecules, from which 6 candidates were selected based on comprehensive docking scores and binding mode analyses.

Application in Developing PARP2 Selective Inhibitors

While we have achieved some success in developing PARP1 selective inhibitors, the approach based on pharmacophore generation does not always prove effective, primarily because the number of defined pharmacophores is typically less than the total pharmacophores present in the molecules. Through CMD-GEN pharmacophore sampling analysis of PARP2 and PARP1, we identified notable differences in the sampling probability of pharmacophore points near GLU763 (GLN332), revealing a critical divergence between the two proteins (Fig. S13). Specifically, Region 1 exhibited a significantly higher sampling density in PARP2, highlighting it as a promising target for selectivity, a finding supported by the benzene ring placement in the selective PARP2 inhibitor UPF1069. In contrast, Region 2 showed exclusive AROM sampling in PARP1, suggesting that an aromatic ring in this region could contribute to PARP1 selectivity. Meanwhile, Region 3, located within a conserved domain of PARP1 and PARP2, displayed variations likely stemming from static conformational differences rather than pharmacophore characteristics. These insights underscore the CMD-GEN model's capability to pinpoint critical pharmacophore regions, facilitating targeted modifications that leverage specific structural features, thus advancing the precision of selective drug development.

Sequence alignment revealed this residue as the divergent position in the PARP1/2 binding region. Docking analysis of UPF1069 showed its binding mode corresponded to the AROM position identified by our model. Utilizing those insight, we introduced a pharmacophore point at this site, generating and screening three

potential molecules through both de novo design and AZD5305-based optimization, as shown in Table S4. Molecular dynamics simulations confirmed the stable binding of these molecules to the PARP2 domain, with energy analysis demonstrating selective affinity for PARP2 (Fig. S9 & Table S5).

Our team synthesized two molecules and tested their biological activity, as shown in Table S6. Both molecules exhibited good activity and PARP2 selectivity, in stark contrast to the Z and Y series. Remarkably, R1 achieved a selectivity ratio of 13-fold for PARP2, even while retaining the N-methyl-5-(piperazin-1-yl)picolinamide moiety typically associated with PARP1 selectivity from AZD5305. These results highlight the model's keen insight into protein pocket residues and its potential to aid in understanding selective binding mechanisms.

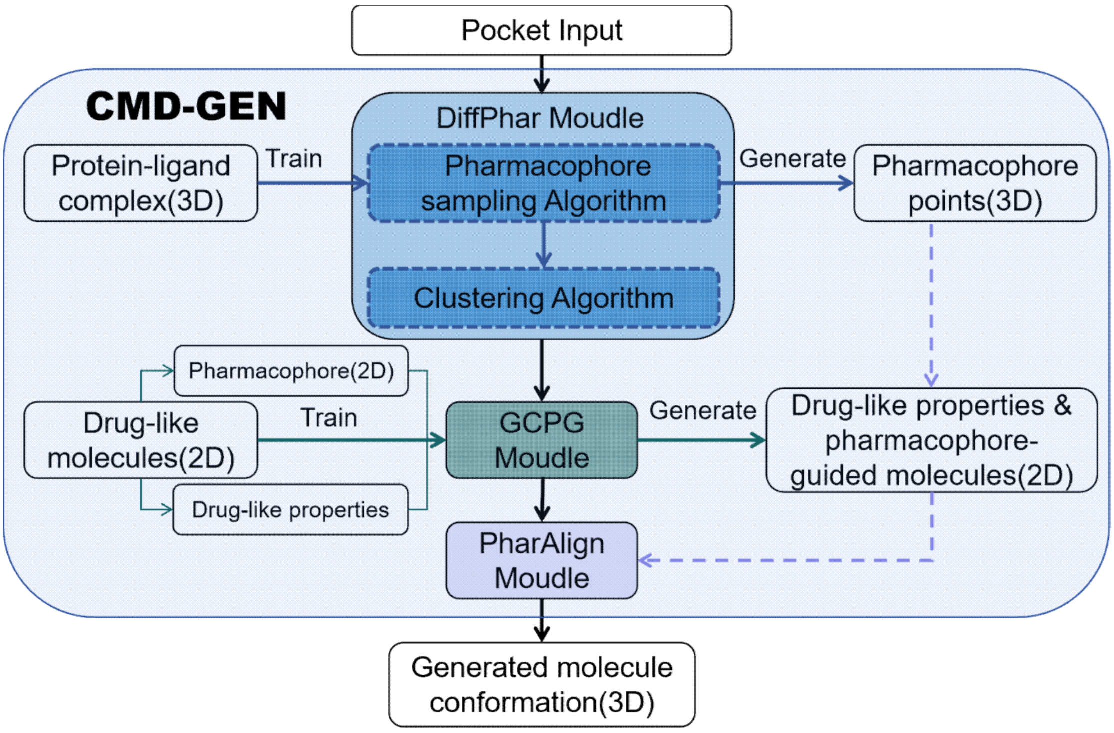

Fig. S1 Flowchart of the overall training and generation process of the CMD-GEN framework. pocket-conditioned three-dimensional pharmacophore sampling module named Diffphar module; Gating Condition Mechanism and Pharmacophore-Based Molecular Generation Module named GCPG module; Molecular Binding Conformation Generation Based on Pharmacophore alignment named PharAlign module.

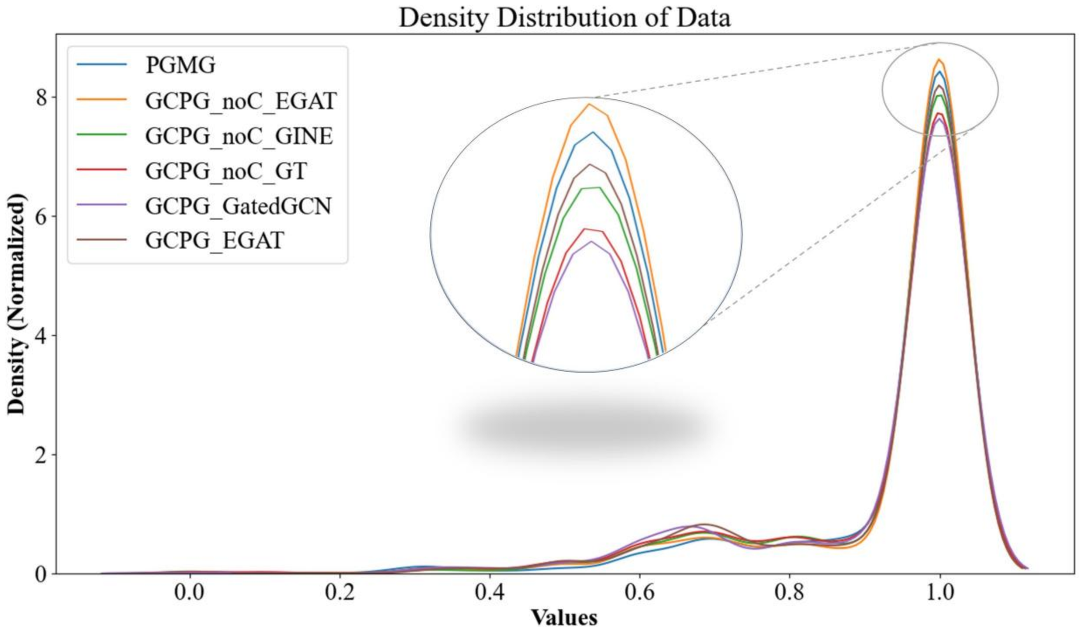

Fig. S2 Comparison of the density distribution plots for match score among models conditioned on pharmacophore.

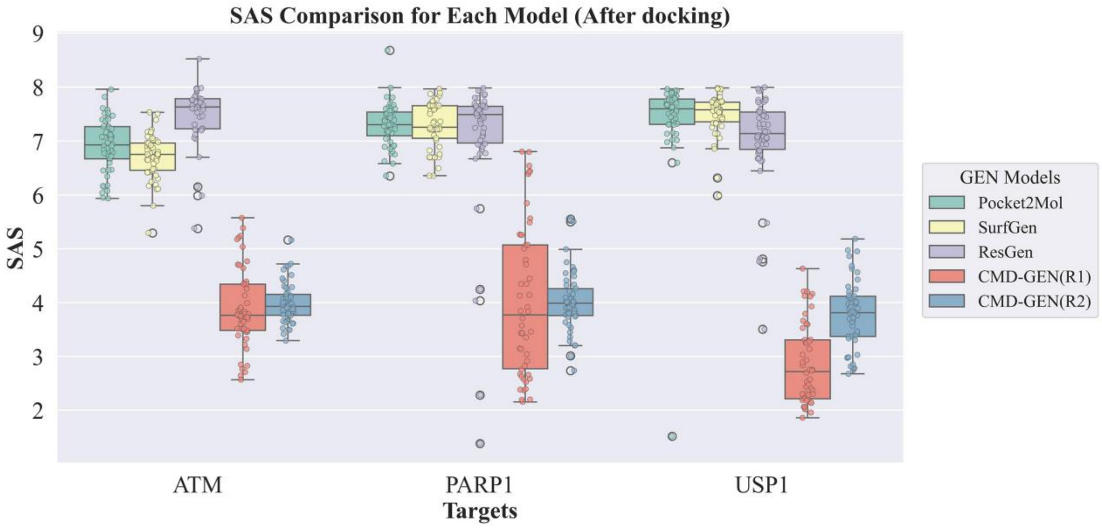

Fig. S3 Boxplot comparison of SAscore after docking between structure-based molecular generation models.

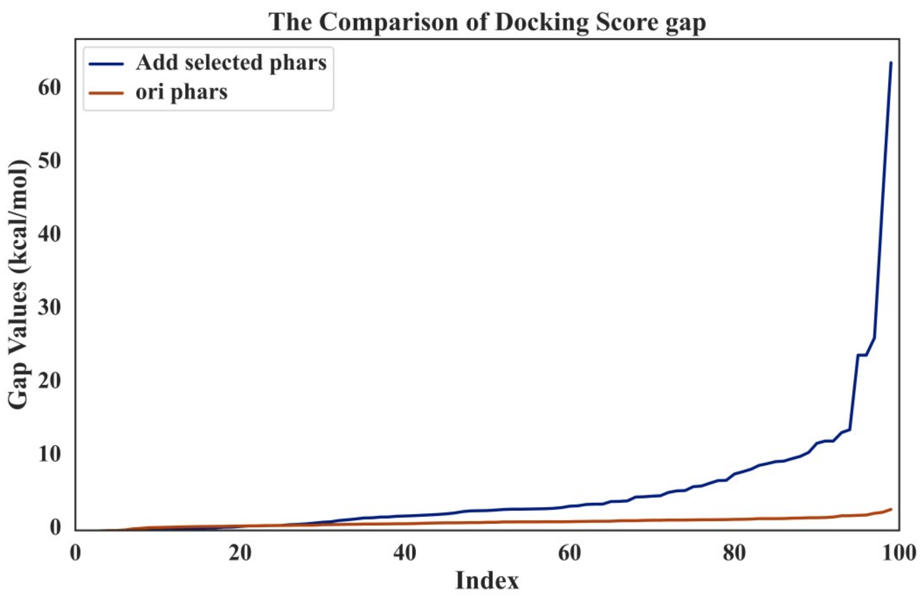

Fig. S4 The Comparison of docking score gap from generated 100 molecules with selectively incorporated pharmacophores through sampling. Evaluate the gap in docking scores between molecules featuring added pharmacophores and those without, with a specific emphasis on PARP1 and PARP2. Quantify the difference by subtracting the docking score for PARP2 from that of PARP1. Arrange the gap values in ascending order and visualize them through a line graph.

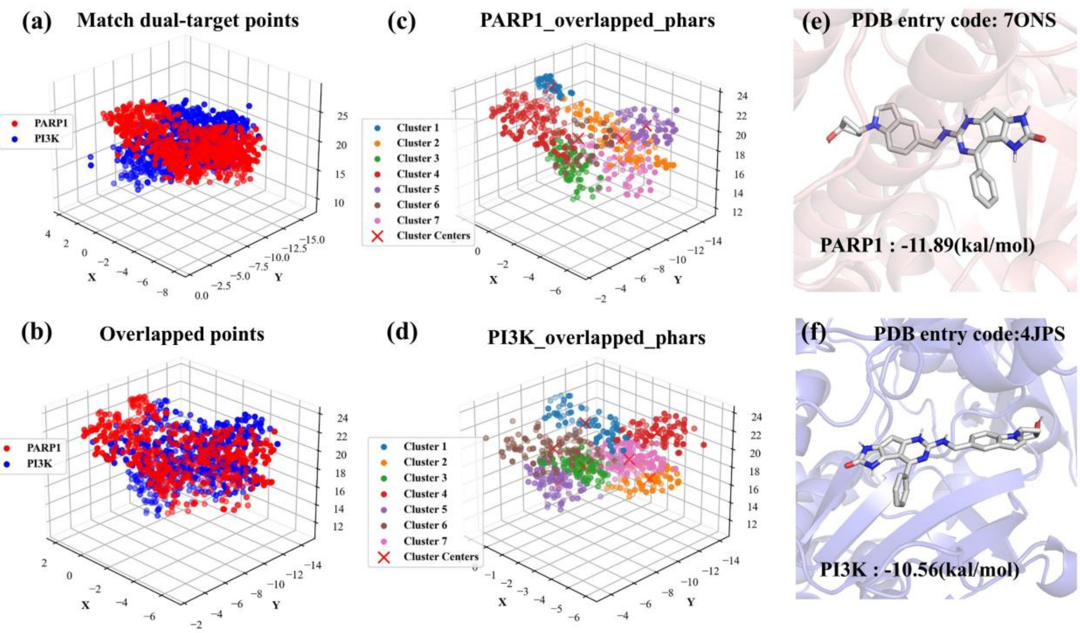

Fig. S5 Illustration of the generation of dual-target molecules based on the CMD-GEN model. The process involves aligning point clouds between two pockets, identifying overlapping regions, randomly sampling pharmacophores within the overlapping point clouds of each target, and merging pharmacophores to obtain dual-target pharmacophores. Finally, utilizing these pharmacophores to generate dual-target inhibitors.

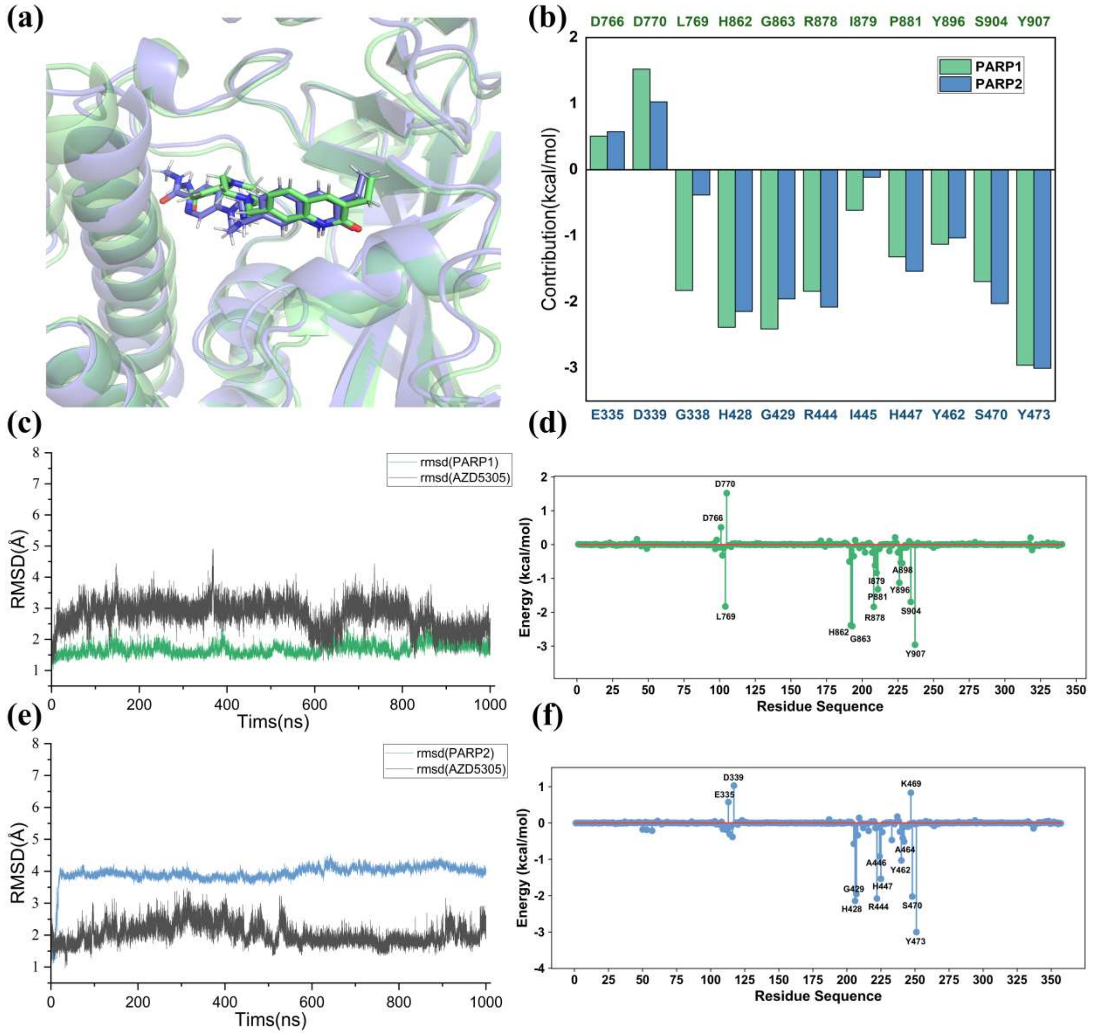

Fig. S6 (a) Docking poses of AZD5305 with PARP1 (PDB ID: 7ONS, green) and PARP2 (PDB ID: 7R59, blue). (b) Comparative energy decomposition of key residues interacting with AZD5305 in PARP1 and PARP2. (c, d) Root mean square deviation (RMSD) of the PARP1 domain in complex with AZD5305 over simulation time and the corresponding MM/GBSA binding free energy decomposition per residue. (e, f) RMSD of the PARP2 domain in complex with AZD5305 over simulation time and the corresponding MM/GBSA binding free energy decomposition per residue. Only residues with significant contributions are labeled.

Olaparib (2014) - Drug Likness

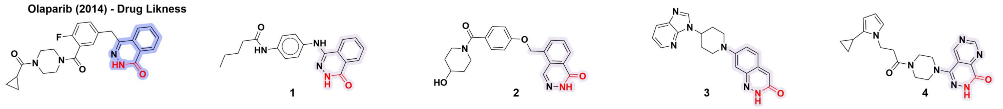

Rucaparib (2016) - Drug Likness

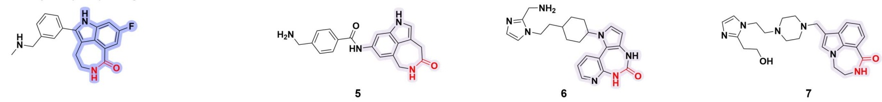

Niraparib (2017) - Drug Likness

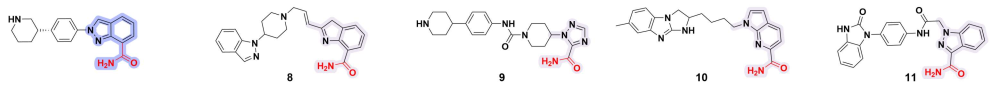

Talazoparib (2018) - Drug Likness

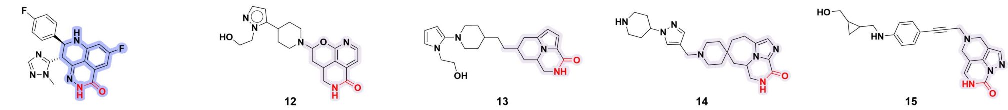

AZD5305 - Drug Likness

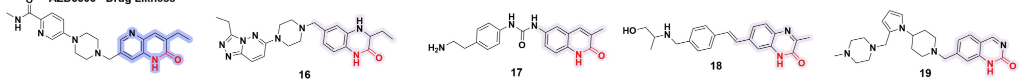

Single-ring Strategy(lactam)

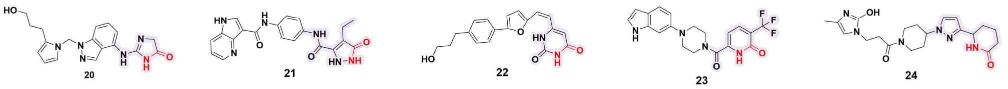

Single-ring Strategy(isosterism)

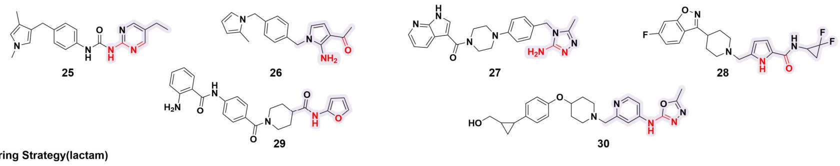

Double-ring Strategy(lactam)

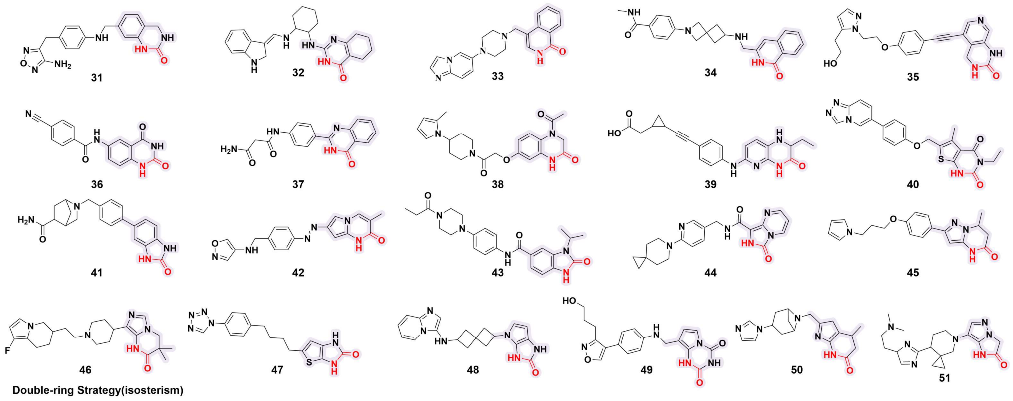

Double-ring Strategy(isosterism)

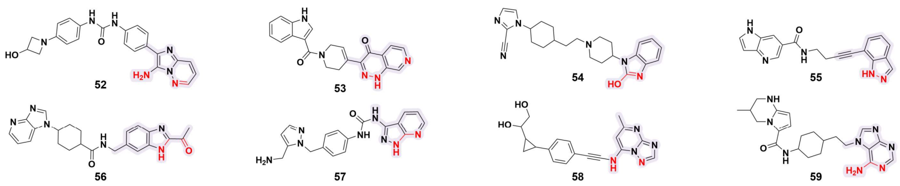

Three-ring Strategy(lactam)

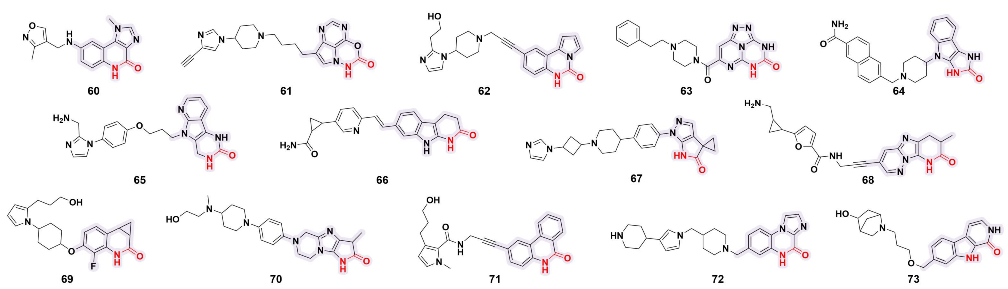

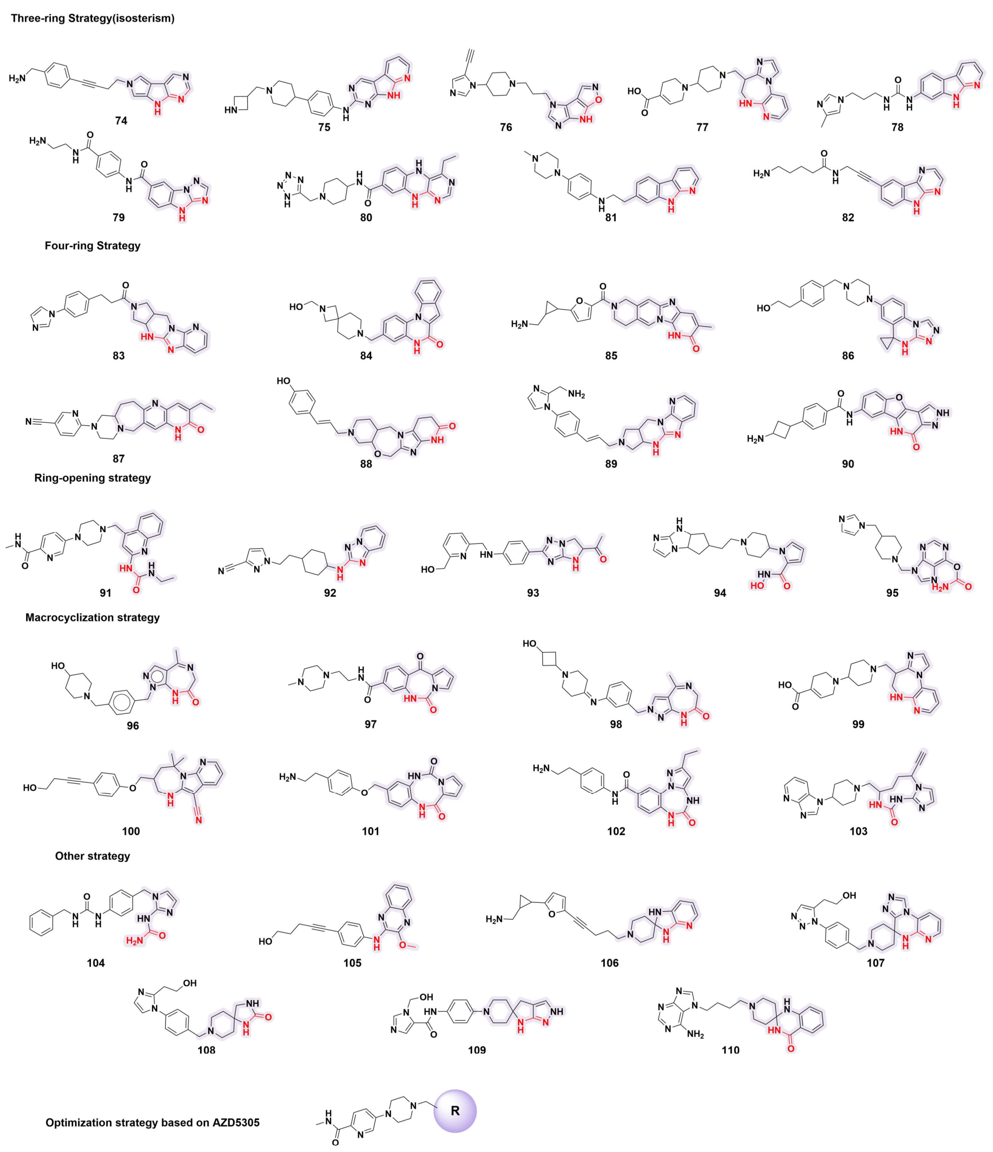

Fig. S7 The molecules generated by the CMD-GEN model were screened to identify 110 potential PARP1 inhibitors, categorized according to pharmacochemical strategies. The optimization approach based on AZD5305 involved the assembly of fragments derived from the generated molecules and N-methyl-5-(piperazin-1-yl)picolinamide scaffold

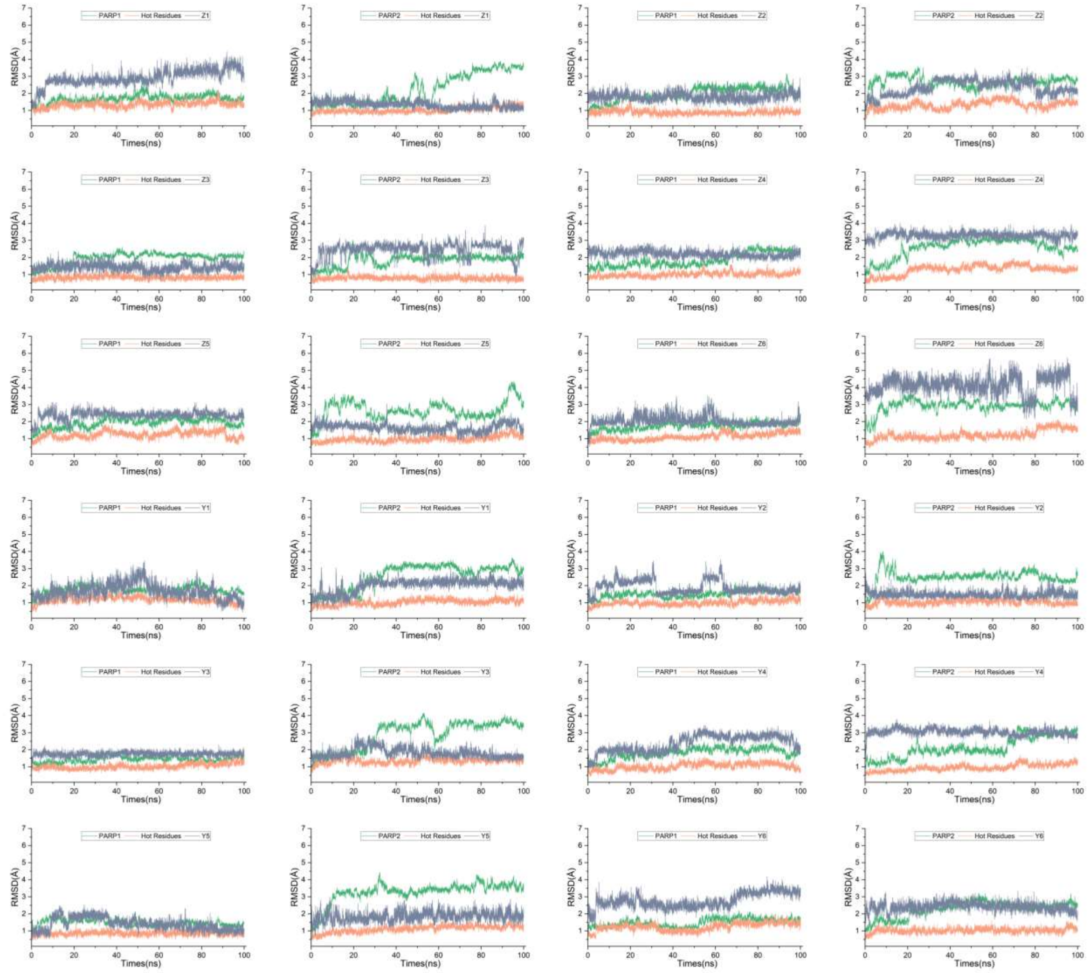

Fig. S8 Root mean square deviation (RMSD) of the PARP1 and PARP2 domains in complex with selected PARP1-selective inhibitor molecules from the Z and Y series over the simulation time.

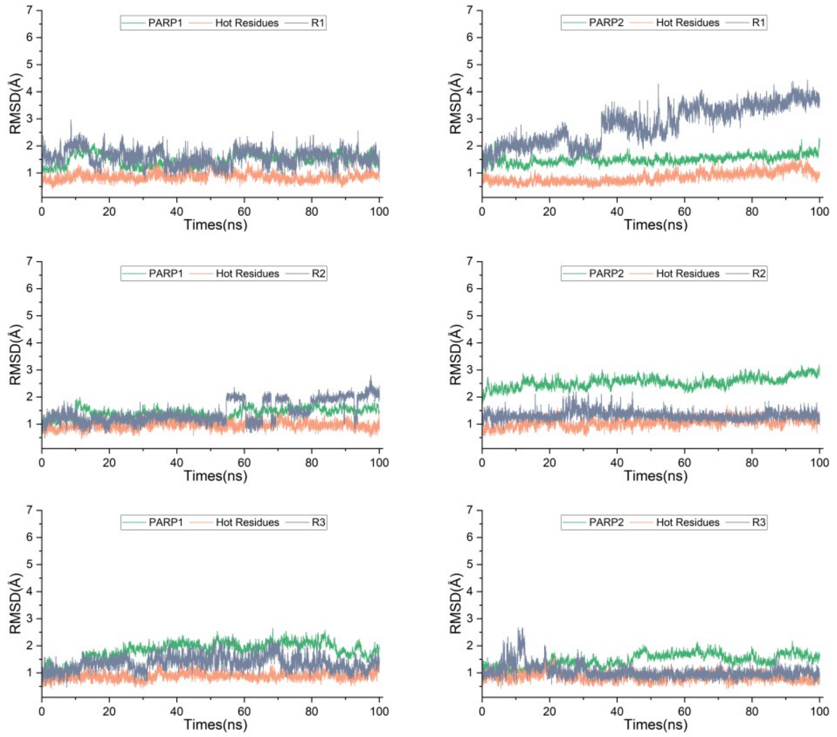

Fig. S9 Root mean square deviation (RMSD) of the PARP1 and PARP2 domains in complex with selected PARP2-selective inhibitor molecules from the R series over the simulation time.

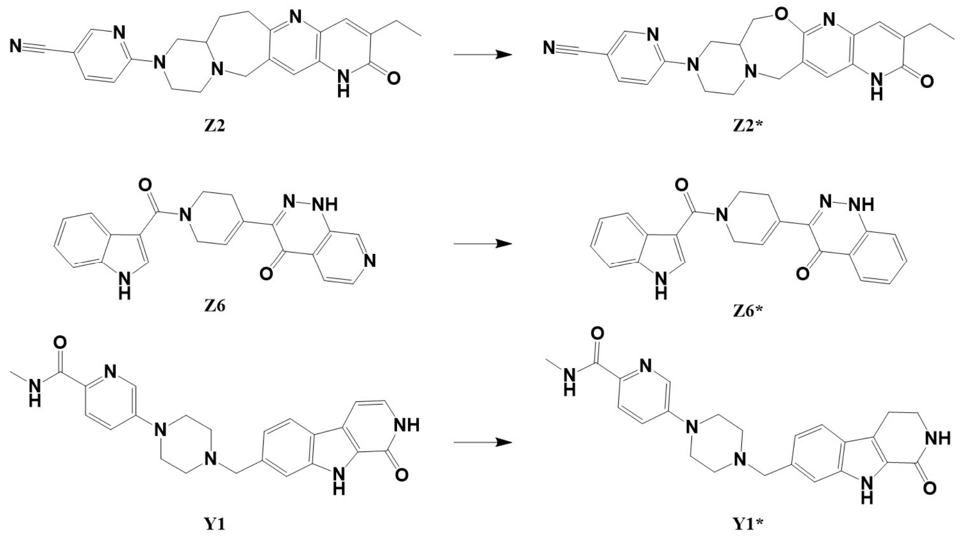

Fig. S10 Structural Simplification Strategy. To improve practical synthetic accessibility, some selected molecules were structurally simplified and annotated with *.

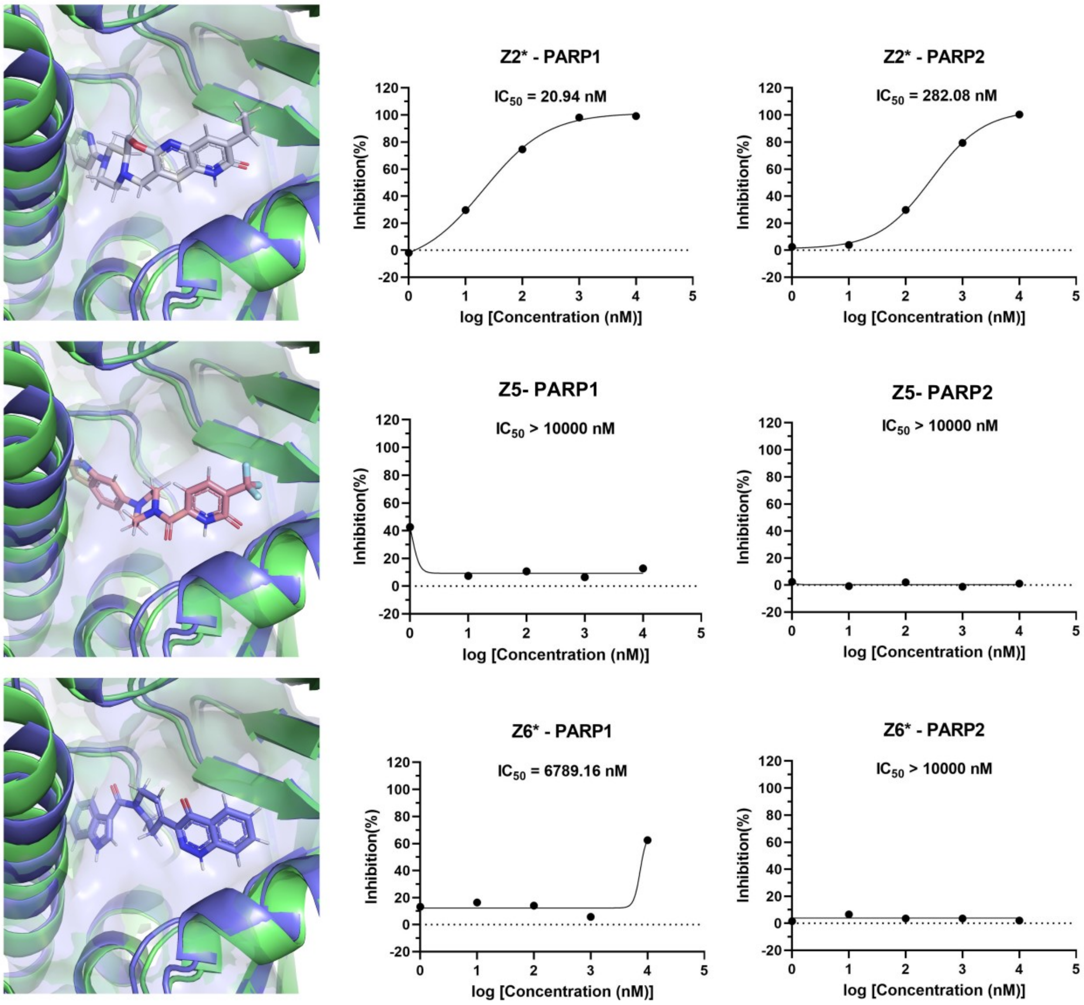

Fig. S11 Binding modes and IC50 values of the Z-Series compounds with PARP1 and PARP2.

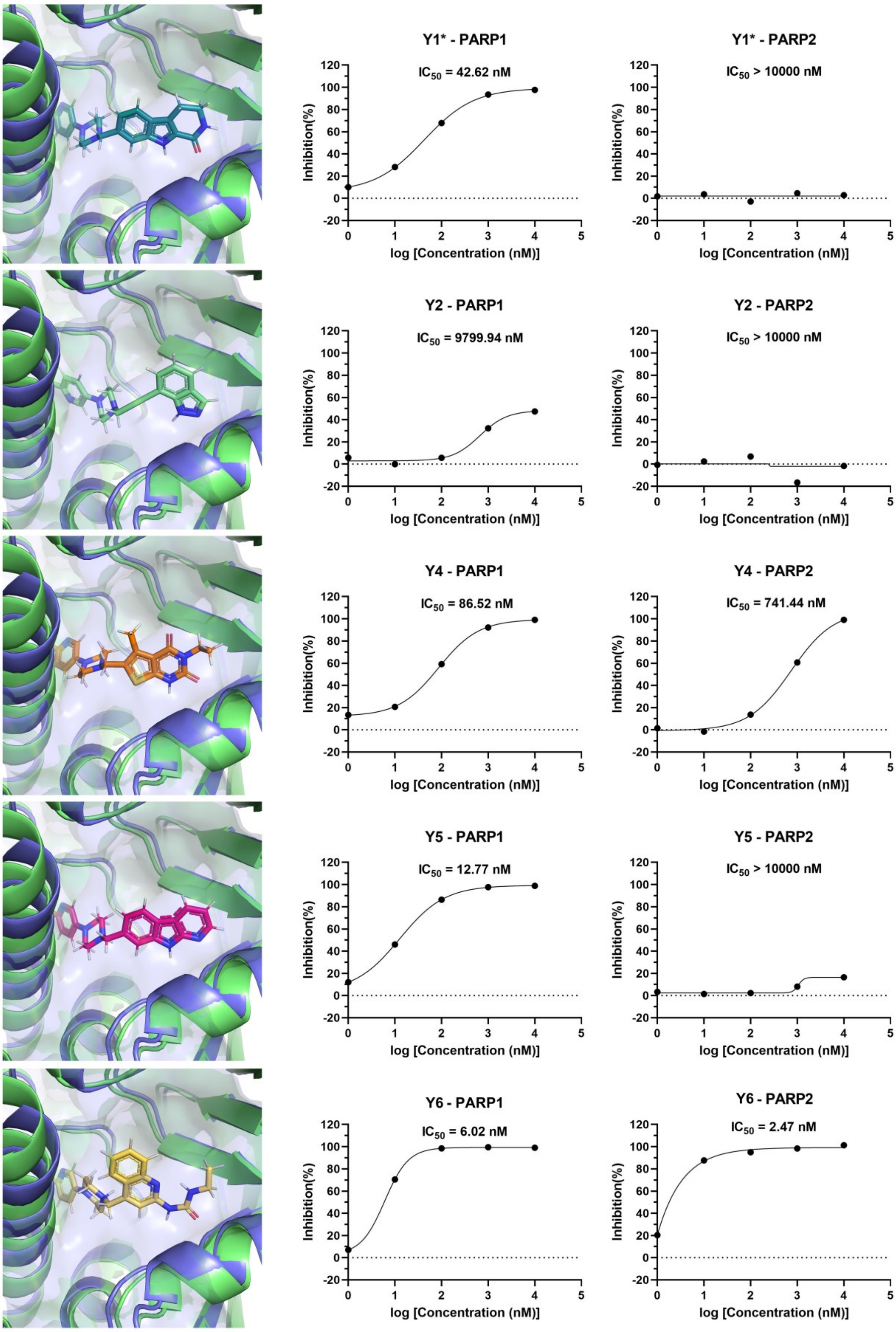

Fig. S12 Binding modes and IC50 values of the Y-Series compounds with PARP1 and PARP2.

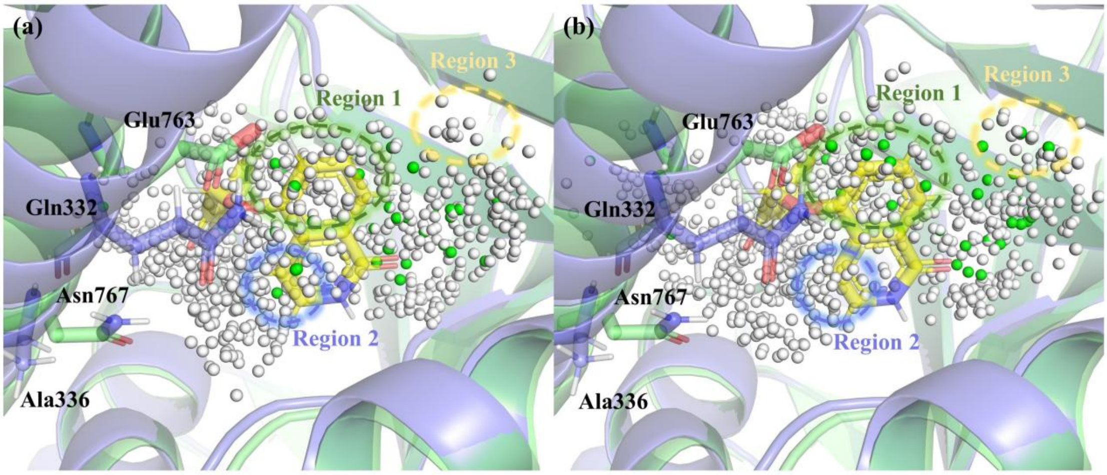

Fig. S13 Pharmacophore point cloud sampling and docking conformations of the selective PARP2 inhibitor UPF1069 in PARP1 and PARP2. (a) Point cloud density map sampled within the PARP1 domain, (b) Point cloud density map sampled within the PARP2 domain. The PARP1 structure is shown in green, the PARP2 structure in blue, and UPF1069 in yellow sticks. Green points represent the sampled AROM (aromatic) pharmacophore, while other sampled pharmacophores are shown in white.

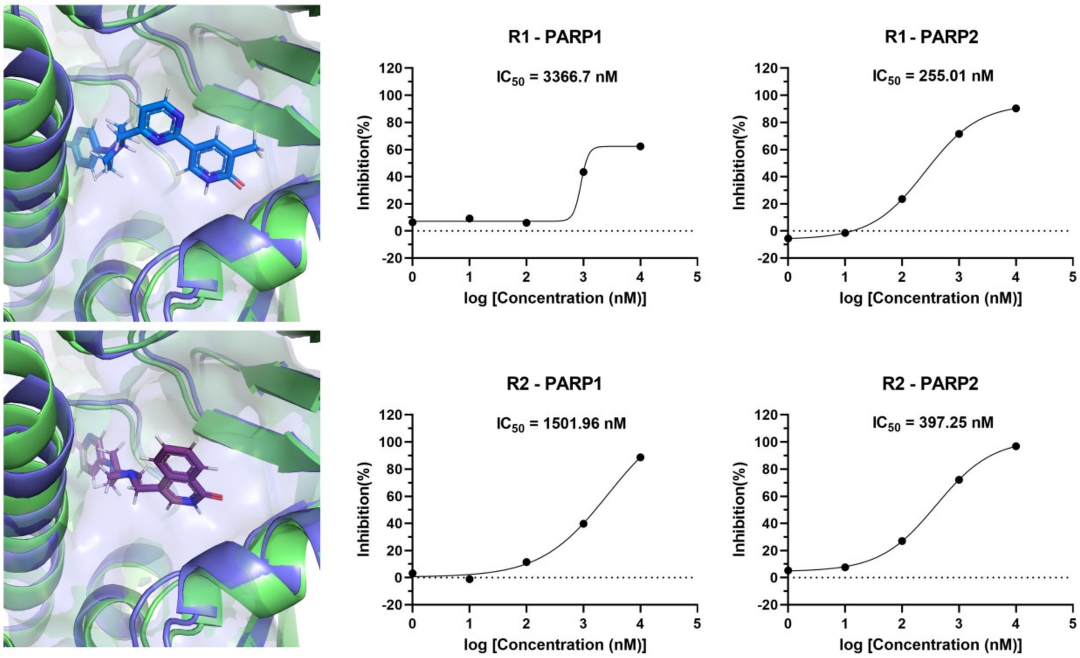

Fig. S14 Binding modes and IC50 values of the R-Series compounds with PARP1 and PARP2.

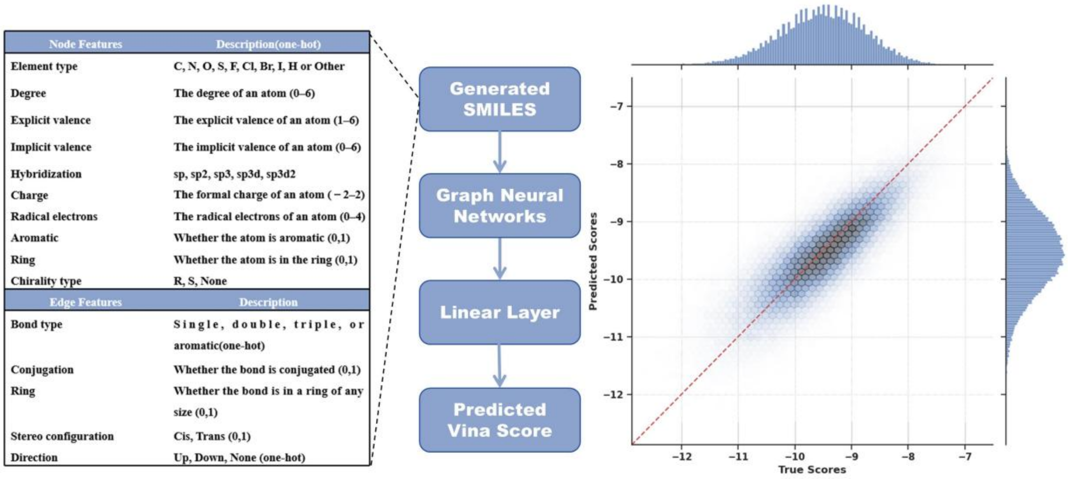

Fig. S15 illustrates key components and performance metrics of the graph neural network prediction model. From left to right, the figure presents: the molecular representations generated by the graph neural network, a schematic diagram of the prediction model architecture, and a Hexbin plot showing the relationship between predicted scores and true scores. During model training, 490,000 molecules were split into training, testing, and validation sets in an 8:1:1 ratio. The model ' s performance was evaluated using the validation set, achieving a correlation coefficient (R) of 0.833. The model was subsequently used to score molecules generated by CMD-GEN, and these scores were employed to fine-tune the CMD-GEN model.

Table S1. The training data for structure-based three-dimensional molecular generation models.

Model	Dataset	Amount of training data
LiGAN[2]	Crossdocked 2020	0.5 million
GraphBP[3]	Crossdocked 2020	0.5 million
Pocket2Mol[4]	Crossdocked 2020	0.1 million
DiffSBDD[5]	Crossdocked 2020	0.1 million
ResGen[6]	Crossdocked 2020	0.1 million
SurfGen[7]	Crossdocked 2020	0.1 million

Table S2. Five pharmacophore models sampled and clustered for each of the three synthetic lethality targets, along with pharmacophore models for the receptor-ligand complex obtained through Schrödinger's Develop Pharmacophore Model. "AROM" is for aromatic features. "HYBL" stands for hydrophobic characteristics. "POSC" represents positive ionizable groups. "HACC" is used for acceptor functionality. "HDON" represents donor attributes. "LHYBL" corresponds to lumped hydrophobic features. "UNKNOWN" encompasses other types of pharmacophores.

PARP1 (PDB ID: 7ONS)				
Pharmacophore Models	Pharmacophore Types	Coordinates		
Receptor- Ligand Interaction	AROM	4.77	42.24	9.12
	AROM	14.97	43.48	6.12
	HDON	13.67	44.16	4.25
	HDON	2.61	42.53	13.74
	HACC	15.83	45.33	4.40
	HYBL	17.18	44.05	7.15
Diff_Phar_1	HACC	15.41	45.17	4.46
	POSC	9.10	40.86	7.29
	HYBL	13.4	42.07	7.04
	LHYBL	15.95	43.04	7.27
	POSC	11.29	41.25	7.05
	AROM	15.64	43.80	6.18
Diff_Phar_2	AROM	16.22	43.69	6.67
	HYBL	9.76	40.96	6.38
	LHYBL	13.83	42.28	6.98
	POSC	11.87	41.21	7.65
	HACC	15.63	45.27	4.51
	HDON	14.65	43.54	5.87
Diff_Phar_3	LHYBL	16.4	43.7	6.82
	HYBL	11.05	40.94	6.82

	HACC	14.87	44.60	4.70
	POSC	11.81	41.66	9.16
	LHYBL	13.28	41.85	7.54
	AROM	14.61	42.97	6.58
	POSC	8.94	41.27	7.26
Diff_Phar_4	LHYBL	16.29	43.76	6.64
	HYBL	9.97	40.57	6.71
	HACC	14.94	44.62	4.77
	POSC	11.82	42.07	8.95
	HYBL	13.58	42.25	6.66
	POSC	7.60	41.79	7.89
Diff_Phar_5	HDON	14.92	44.65	4.67
	HDON	10.66	41.14	8.58
	LHYBL	16.76	43.87	6.83
	HYBL	5.84	42.63	9.07
	HACC	9.95	41.02	6.46
	POSC	14.79	42.95	6.70
USP1 (PDB ID: 8A9K)				
Pharmacophore Models	Pharmacophore Types	Coordinates		
Receptor- Ligand Interaction	AROM	166.31	123.58	126.97
	AROM	162.45	119.73	130.33
	AROM	161.04	119.24	125.64
	AROM	158.60	119.84	122.50
	HACC	159.74	119.82	122.19
	HACC	164.40	122.87	130.06
	HYBL	158.85	120.09	119.51
Diff_Phar_1	LHYBL	165.88	124.00	127.65

	HYBL	161.89	120.42	126.47
	AROM	164.26	122.30	127.64
	POSC	165.46	123.51	129.79
	HDON	162.36	120.37	128.33
	LHYBL	159.93	120.87	124.91
	HACC	162.36	121.39	124.64
Diff_Phar_2	HDON	164.00	121.59	129.71
	LHYBL	161.20	120.62	125.85
	POSC	165.88	123.74	129.35
	HDON	161.48	119.09	128.66
	AROM	164.20	122.43	127.33
\Diff_Phar_3	POSC	164.00	122.85	131.26
	UNKNOWN	158.48	120.90	121.67
	POSC	162.22	119.56	128.33
	HDON	162.69	120.90	131.92
	LHYBL	165.73	123.81	128.28
	AROM	160.62	120.84	125.06
Diff_Phar_4	HACC	160.20	121.02	124.7
	HDON	166.53	124.18	129.65
	LHYBL	161.57	120.47	126.48
	HDON	162.77	120.21	128.86
	POSC	164.24	122.52	127.24
	POSC	164.70	122.55	129.94
	AROM	165.49	123.64	128.06
Diff_Phar_5	UNKNOWN	159.15	121.08	123.99
	POSC	165.64	123.74	129.72
	HACC	161.99	119.62	129.90

	AROM	165.90	123.88	127.93
	HDON	163.43	120.73	128.28
	AROM	164.03	122.33	126.85
	LHYBL	161.17	120.74	125.82

ATM (PDB ID: 7NI4)

Pharmacophore Models	Pharmacophore Types	Coordinates		
Receptor- Ligand Interaction	AROM	190.91	239.38	235.63
	HYBL	194.72	241.06	233.54
	HACC	189.79	238.54	235.63
	AROM	189.67	244.42	232.05
	HACC	190.07	245.52	232.02
	HYBL	186.65	240.60	232.15
Diff_Phar_1	LHYBL	191.45	245.21	232.96
	HDON	190.60	238.00	236.05
	LHYBL	193.46	242.47	235.55
	AROM	191.65	240.03	235.56
	HACC	189.23	245.01	230.55
	POSC	188.92	241.82	233.07
	HACC	188.65	239.69	234.51
Diff_Phar_2	HYBL	189.10	244.17	231.11
	HDON	188.49	240.83	233.69
	POSC	191.19	239.72	235.47
	LHYBL	192.51	245.10	232.80
	LHYBL	192.59	243.31	235.06
Diff_Phar_3	AROM	189.03	243.62	231.46
	POSC	188.65	240.50	233.91
	LHYBL	194.4	241.63	236.03

	HDON	191.01	239.30	235.54
	LHYBL	192.38	244.81	233.64
	POSC	191.30	242.53	234.33
	HACC	189.18	245.06	230.53
Diff_Phar_4	HACC	188.64	245.64	229.7
	LHYBL	192.91	242.84	235.16
	AROM	189.34	240.77	234.1
	AROM	189.45	244.27	231.45
	HDON	191.93	245.56	231.48
	POSC	190.68	239.38	235.47
	LHYBL	188.13	241.17	232.97
Diff_Phar_5	HYBL	189.11	242.89	232.44
	HACC	192.21	239.49	235.88
	LHYBL	193.54	242.28	235.03
	POSC	189.01	244.59	230.17
	HDON	188.18	239.56	234.15
PARP1 & PARP2 (PDB ID: 7R59)				
Selected_phars_parp1	HYBL	12.63	37.13	5.70
	HDON	3.61	45.25	7.76
PARP1 & PARP2 (PDB ID: 7R59)				
Selected_phars_parp2	AROM	13.72	39.75	8.91

Table S3. The comparison of runtime between models. "Time" represents the average time spent on generating one molecule. Numbers in parentheses indicate standard deviations.

Method	Target	Mean Time(s)
Pocket2Mol	ATM	7.75(±1.53)
	PARP1	15.72(±2.65)
	USP1	8.63(±0.62)
ResGen	ATM	48.53(±7.88)
	PARP1	32.87(±6.83)
	USP1	70.43(±9.21)
SurfGen	ATM	42.43(±4.52)
	PARP1	48.48(±3.98)
	USP1	46.83(±4.26)
CMD-GEN (generate molecules)	ATM	0.0015(±5e-5)
	PARP1	0.0014(±5e-5)
	USP1	0.0014(±1e-4)
CMD-GEN (generate molecules + align)	ATM	0.85(±0.43)
	PARP1	1.02(±0.24)
	USP1	0.94(±0.16)

Table S4. PARP1/2 selective inhibitors identified using a generate-screen strategy within de novo design and optimization development modes.

**PARP1 Selective Inhibitors: *De Novo* Design Mode Based on Target Structure (Mode 1)**		
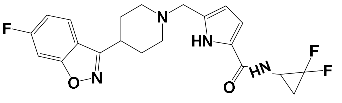	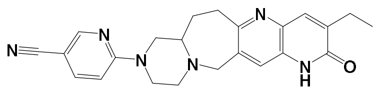	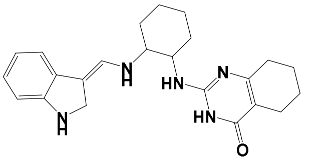
**Z1**	**Z2**	**Z3**
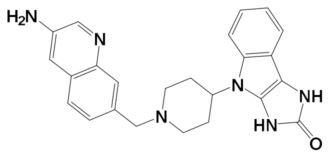	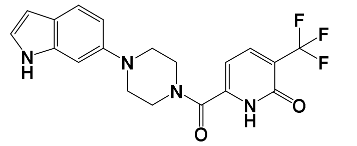	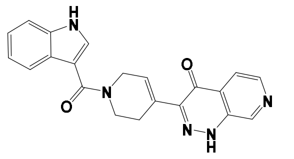
**Z4**	**Z5**	**Z6**
**PARP1 Selective Inhibitors: Optimization Based on Target Structure and AZD5305 (Mode 2)**		
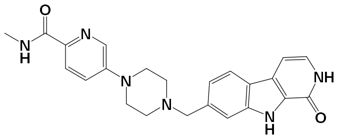	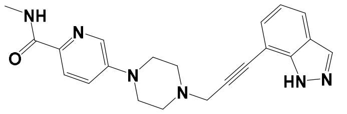	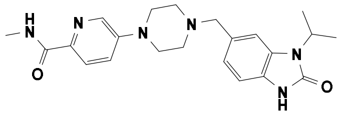
**Y1**	**Y2**	**Y3**
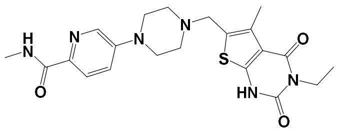	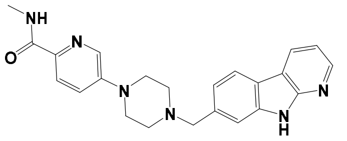	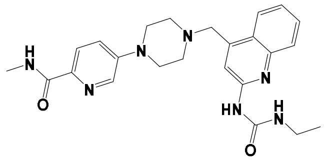
**Y4**	**Y5**	**Y6**
**PARP2 Selective Inhibitors**		
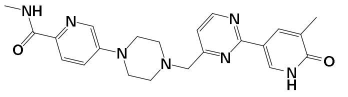	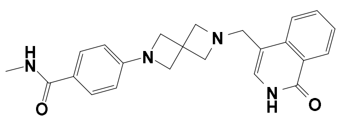	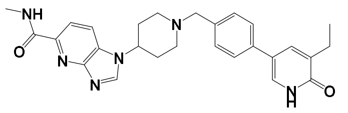
**R1**	**R2**	**R3**

Table S5. Binding free energies of all selected molecules with PARP1 and PARP2.

Index	System	E_{vdW}	E_{ele}	$G_{sol,el}$	$G_{sol,nonel}$	E_{gas}	ΔG_{bind}^{Cal}
AZD 5305	PARP1	-58.78(3.61)	-117.78(25.97)	129.11(23.87)	-6.49(0.31)	-176.56(27.77)	-53.94(5.73)
	PARP2	-58.91(3.03)	-122.01(11.92)	138.16(12.03)	-6.37(0.28)	-180.93(12.62)	-49.14(3.29)
Z1	PARP1	-56.25(1.68)	-109.71(9.86)	123.00(9.86)	-6.63(0.09)	-165.96(9.31)	-49.60(2.50)
	PARP2	-55.97(3.41)	-125.57(7.62)	141.81(7.08)	-6.27(0.09)	-181.55(6.64)	-46.01(2.07)
Z2	PARP1	-56.47(2.33)	-38.63(3.12)	51.70(1.07)	-6.29(0.12)	-95.10(2.22)	-49.69(2.12)
	PARP2	-50.57(1.98)	-27.04(2.89)	41.08(3.26)	-5.79(0.13)	-77.61(3.38)	-42.32(2.73)
Z3	PARP1	-61.47(2.74)	-32.93(3.03)	42.92(2.06)	-6.72(0.21)	-94.40(2.04)	-58.20(1.69)
	PARP2	-55.84(3.38)	-23.01(3.93)	29.46(2.24)	-6.14(0.23)	-78.85(4.09)	-55.52(3.56)
Z4	PARP1	-58.66(2.43)	-108.66(10.18)	126.40(9.84)	-6.38(0.06)	-167.33(9.12)	-47.30(2.38)
	PARP2	-57.10(2.81)	-123.17(9.98)	141.09(9.88)	-6.21(0.15)	-180.28(9.40)	-45.40(2.01)
Z5	PARP1	-50.80(1.69)	-38.01(2.73)	45.52(1.84)	-6.03(0.03)	-88.81(1.72)	-49.32(0.46)
	PARP2	-48.27(1.34)	-25.96(2.97)	33.66(2.23)	-5.51(0.14)	-74.24(3.24)	-46.08(2.87`)
Z6	PARP1	-52.07(3.09)	-35.44(3.04)	48.87(2.16)	-5.77(0.03)	-87.52(4.38)	-44.42(3.29)
	PARP2	-47.06(3.15)	-32.55(4.60)	49.23(2.67)	-5.44(0.25)	-79.61(6.01)	-35.83(4.50)
Y1	PARP1	-59.83(2.88)	-42.45(3.08)	55.86(2.68)	-6.63(0.16)	-102.29(3.16)	-53.05(3.42)
	PARP2	-55.67(2.98)	-24.53(6.30)	44.50(4.16)	-6.23(0.21)	-80.21(5.80)	-41.93(4.43)
Y2	PARP1	-56.73(2.09)	-25.60(3.24)	39.08(2.85)	-6.22(0.14)	-82.34(3.11)	-49.47(2.01)
	PARP2	-55.42(2.46)	-23.69(3.79)	38.21(3.23)	-6.00(0.14)	-79.12(4.11)	-46.92(2.94)
Y3	PARP1	-56.64(2.32)	-106.34(5.98)	118.79(6.35)	-6.36(0.15)	-162.98(6.65)	-50.55(4.45)
	PARP2	-53.80(3.03)	-114.87(9.74)	128.21(9.32)	-5.89(0.27)	-168.67(9.88)	-46.35(3.51)
Y4	PARP1	-61.69(1.64)	-113.05(9.00)	129.08(8.76)	-6.35(0.07)	-174.74(9.16)	-52.02(1.55)
	PARP2	-62.29(2.10)	-104.41(5.19)	122.39(5.09)	-6.61(0.16)	-166.70(4.62)	-50.92(2.40)
Y5	PARP1	-62.09(3.26)	-87.76(6.85)	104.98(6.99)	-5.99(0.15)	-149.86(7.15)	-50.86(2.76)
	PARP2	-58.46(1.84)	-110.78(8.71)	127.96(8.45)	-5.87(0.09)	-169.24(9.02)	-47.15(1.72)
Y6	PARP1	-62.57(1.48)	-111.61(7.64)	125.27(8.02)	-6.93(0.15)	-174.19(6.55)	-55.85(2.78)
	PARP2	-65.66(1.68)	-113.92(8.62)	132.45(9.88)	-7.13(0.28)	-179.59(9.28)	-54.27(2.96)

R1	PARP1	-61.55(3.05)	-116.21(10.54)	129.85(10.59)	-6.85(0.17)	-177.77(10.84)	-54.77(2.62)
	PARP2	-62.63(2.44)	-127.89(7.62)	141.74(8.34)	-7.09(0.23)	-190.53(8.63)	-55.88(3.33)
R2	PARP1	-52.33(2.86)	-105.79(5.95)	114.16(5.72)	-5.90(0.11)	-158.13(5.79)	-49.87(3.49)
	PARP2	-58.57(3.43)	-143.73(6.60)	152.47(6.06)	-6.66(0.09)	-202.30(6.79)	-56.49(3.76)
R3	PARP1	-68.24(2.97)	-118.64(6.80)	140.23(6.32)	-7.05(0.22)	-186.88(5.92)	-53.71(2.38)
	PARP2	-70.32(3.16)	-124.14(7.74)	144.11(6.88)	-7.46(0.13)	-194.46(7.86)	-57.81(3.29)

$\boldsymbol{E_{vdW}}$ and $\boldsymbol{E_{ele}}$ represent van der Waals energy and electrostatic energy, respectively. $\boldsymbol{G_{sol,el}}$ and $\boldsymbol{G_{sol,nonel}}$ represent the electrostatic and non-electrostatic components of the solvation free energy, respectively, and $\boldsymbol{G_{sol}}$ represents their sum. $\boldsymbol{E_{gas}}$ represents the system energy in the gas phase, which is the sum of $\boldsymbol{E_{vdW}}$ and $\boldsymbol{E_{ele}}$. $\Delta\boldsymbol{G_{bind}^{Cal}}$ is the calculated binding free energy. The calculations were performed without considering entropy effects.[8] The unit is kcal/mol.

Table S6. Biological Activity and Selectivity of PARP2-Selective Molecules Generated by CMD-GEN.

Entry	Structure	PARP1 Enzyme			PARP2 Enzyme			Selectivity PARP-1/2
		% control @ 0.01 μM[a]	% control @ 1 μM[a]	IC_{50} (nM)[b]	% control @ 0.01 μM[a]	% control @ 1 μM[a]	IC_{50} (nM)[b]	
**R1**	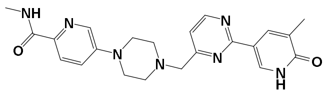	9.1	62.3	3366.7	-1.5	90.3	255.0	13.20
**R2**	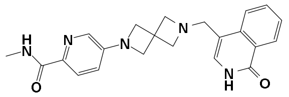	-1.2	88.7	1501.9	7.6	96.8	397.2	3.78
**Olaparib**				0.7			1.4	

[a] % control, The inhibitory activity was evaluated at the compound; [b] IC50, compound's concentration required to inhibit PARP1/2 enzyme activity by 50%;

Synthetic method

Scheme 1. General Preparation of Compound Z2*[a]

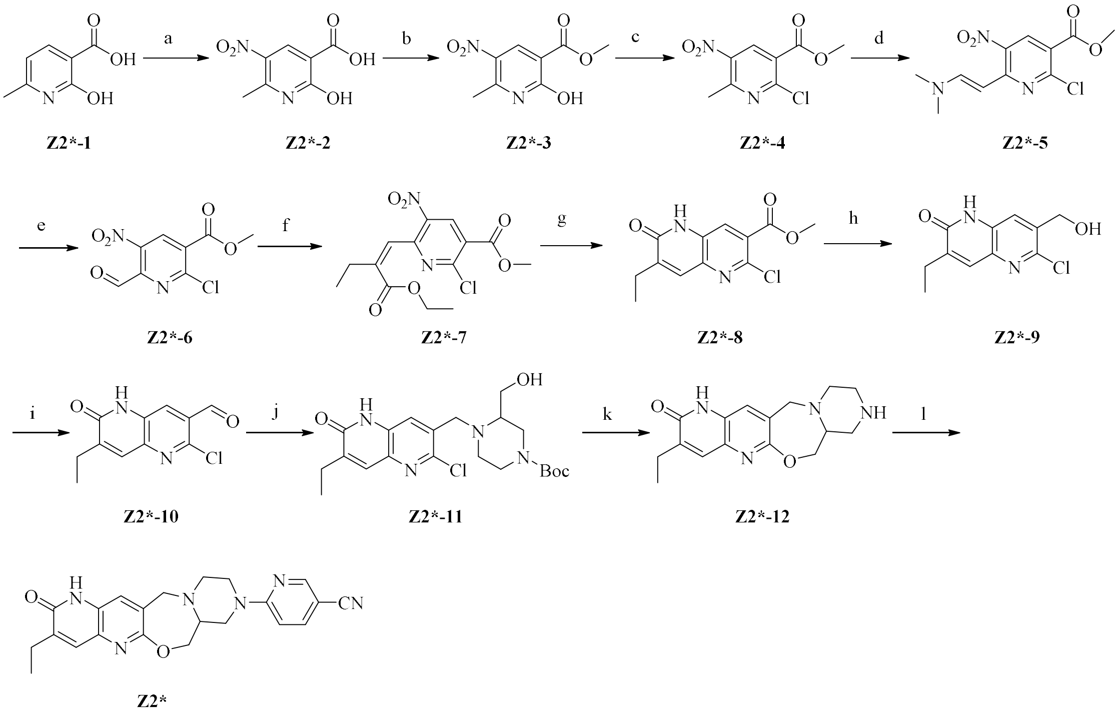

[a]Reagents and conditions: (a) KNO_3, H_2SO_4, 90 ºC 4h; (b) MeOH, H_2SO_4, 70 ºC 4h; (c) $POCl_3$, 110 ºC 3h; (d) DMF-DMA, 100 ºC 3h; (e) THF/H_2O = 1/1 (*v*/*v*), rt, 12h; (f) THF, -70 ºC 2h; (g) AcOH, Fe, 80 ºC 3h; (h) THF, DIBAL-H, rt, 1h; (i) DMP, DCM, rt, 2h; (j) AcOH, $NaBH_3CN$, MeOH, 64 ºC 3h; (k) *t*-BuOK, NMP, 180 ºC 3h; (l) DIPEA, NMP, 120 ºC 2h.

General Preparation of Compound Z2*. Step a: Add **Z2*-1** to concentrated sulfuric acid. At room temperature, add potassium nitrate in portions. Heat the mixture to 90°C and maintain this temperature for 4 hours. After the reaction, pour the mixture into water, leading to the precipitation of a solid. Filter to collect the solid and dry under reduced pressure to obtain **Z2*-2**.

Step b: Add methanol to **Z2*-2**, then slowly add concentrated sulfuric acid at room temperature. Heat the mixture to 70°C and maintain this temperature for 4 hours. Concentrate the reaction mixture under reduced pressure, allowing a solid to precipitate. Filter to collect the solid and dry under reduced pressure to obtain **Z2*-3**.

Step c: Add phosphorus oxychloride to **Z2*-3** and heat to 110°C for 3 hours. After the reaction, concentrate the mixture under reduced pressure, then add the concentrated solution to ice water. Extract with ethyl acetate, wash the organic layer with water three times, and concentrate it under reduced pressure. Purify the resulting crude product by column chromatography to obtain **Z2*-4**.

Step d: Add **Z2*-4** to DMF, then add DMF-DMA. Heat to 100°C and stir for 3 hours. After the reaction, concentrate the mixture under reduced pressure, then add the concentrated solution to water. Extract with ethyl acetate, wash the organic layer with water three times, and concentrate it under reduced pressure. Purify the resulting crude product by column chromatography to obtain **Z2*-5**.

Step e: Add THF/water (1*v*/1*v*) to **Z2*-5**. Cool the mixture to 0°C and then add sodium periodate. Stir at room temperature for 12 hours. After the reaction is complete, add the mixture to water and extract with ethyl acetate. Wash the organic layer with water three times, then concentrate the organic layer under reduced pressure. Purify the resulting crude product by column chromatography using a petroleum ether:ethyl acetate gradient to obtain **Z2*-6**.

Step f: Add sodium hydride to tetrahydrofuran and cool to 0°C. Add (1-ethoxy-1-oxo-2-butyl)phosphonic acid diethyl ester and stir at room temperature for 2 hours. Cool the reaction mixture to -70°C, then add **Z2*-6**. Maintain the reaction at -70°C for 2 hours. After the reaction is complete, quench the reaction mixture with water and extract with ethyl acetate. Wash the organic phase with water three times, then concentrate the organic phase under reduced pressure. Purify the crude product by column chromatography to obtain **Z2*-7**.

Step g: Add **Z2*-7** and iron powder to acetic acid ice. Heat to 80°C and stir for 3 hours. After the reaction is complete, filter the reaction mixture, concentrate the filtrate under reduced pressure, and add the concentrate to water. Adjust the pH to around 8 with saturated sodium bicarbonate, then extract with dichloromethane. Wash the organic phase with water three times, dry over anhydrous sodium sulfate, filter,

and concentrate the filtrate under reduced pressure to obtain **Z2*-8**.

Step h: Add **Z2*-8** to tetrahydrofuran at room temperature. Add DIBAL-H and stir for 1 hour. After the reaction is complete, add the reaction mixture to water. Extract with dichloromethane: Methanol (5:1), dry the organic phase over anhydrous sodium sulfate, filter, and concentrate the filtrate under reduced pressure to obtain **Z2*-9**.

Step i: Add **Z2*-9** to dichloromethane, then add DMP. Stir at room temperature for 2 hours. After the reaction is complete, add the reaction mixture to water. Extract with dichloromethane: methanol (10:1), concentrate the organic phase, and purify the resulting crude product by column chromatography to obtain **Z2*-10**.

Step j: Add **Z2*-10** and 3-(hydroxymethyl)piperazine-1-carboxylic acid tert-butyl ester to methanol, then add 1 drop of glacial acetic acid. Heat to 64°C and react for 3 hours. Add sodium cyanoborohydride and continue stirring for 2 hours. After the reaction is complete, concentrate the reaction mixture under reduced pressure. Purify the resulting crude product by column chromatography to obtain **Z2*-11**.

Step k: Add **Z2*-11** and potassium tert-butoxide to NMP (N-methylpyrrolidone), then heat to 180°C and stir for 3 hours. After the reaction is complete, add the reaction mixture to water and extract with ethyl acetate. Wash the organic phase with water three times, then concentrate the organic phase under reduced pressure. Purify the resulting crude product by column chromatography to obtain **Z2*-12**.

Step l: **Z2*-12** and 6-bromopyridine-3-carbonitrile in NMP. Add DIPEA and heat to 120°C for 2 hours. After the reaction, add the mixture to water and extract with dichloromethane. Concentrate the organic phase to obtain **Z2***. ^{1}H NMR (400 MHz, DMSO-d_6) δ 12.09 (s, 1H), 8.56 (d, J = 2.3 Hz, 1H), 7.98 (dd, J = 9.0, 2.4 Hz, 1H), 7.74 (s, 1H), 7.63 (s, 1H), 7.06 (d, J = 9.1 Hz, 1H), 4.58 (d, J = 13.5 Hz, 2H), 4.37 (d, J = 39.9 Hz, 4H), 3.91 – 3.82 (m, 1H), 3.27 – 2.94 (m, 4H), 2.59 – 2.51 (m, 2H), 1.17 (t, J = 7.4 Hz, 3H). ^{13}C NMR (101 MHz, DMSO-d_6) δ 168.57, 166.27, 161.79, 160.14, 159.20, 152.86, 142.46, 140.87, 135.04, 131.63, 128.21, 125.71, 118.89, 107.49, 70.46, 70.43, 23.58, 12.88, -10.89.

Scheme 2. General Preparation of Compound Z5[a]

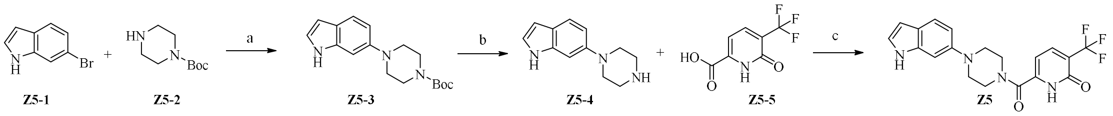

[a]Reagents and conditions: (a) RuPhos Pd G3, Cs_2CO_3, 1,4-dioxane, 100 ºC, 12h; (b) HCl in 1,4-dioxane, rt, 1h; (c) DIPEA, HATU, DCM, rt, 5h.

General Preparation of Compound Z5. Step a: A solution containing **Z5-1** and of **Z5-2** in 1,4-dioxane was treated with Cs_2CO_3 and RuPhos Pd G3. After displacing nitrogen, the reaction mixture was heated to 100°C for 12 h. Upon completion of the reaction, the mixture was subjected to extraction by adding water and ethyl acetate three times. The combined organic phases were then dried using anhydrous sodium sulfate, filtered, and evaporated to dryness. The resulting residue was purified by silica gel chromatography to give **Z5-3**.

Step b: To a solution of **Z5-3** in MeOH and HCl in 1,4-dioxane. The mixture was stirred at room temperature for 1h. After completion, it was concentrated under reduced pressure to give the **Z5-4**.

Step c: To a solution of compound **Z5-4** in DCM was added **Z5-5**, HATU, and DIPEA . The mixture was stirred at room temperature for 5 h. After completion, the mixture was added water and extracted with DCM. The combined organic phase was washed with brine, deied over anhydrous sodium sulfate and concentrated under reduced pressure. The residue was purified by chromatography over silica gel to give the compound **Z5**.

^{1}H NMR (400 MHz, DMSO-d_6) δ 10.82 (t, J = 2.3 Hz, 1H), 9.05 (dt, J = 1.9, 0.9 Hz, 1H), 8.42 – 8.35 (m, 1H), 7.87 (dt, J = 8.3, 0.8 Hz, 1H), 7.40 (d, J = 8.6 Hz, 1H), 7.17 (dd, J = 3.1, 2.3 Hz, 1H), 6.88 (d, J = 2.1 Hz, 1H), 6.80 (dd, J = 8.6, 2.1 Hz, 1H), 6.30 (ddd, J = 3.0, 1.9, 0.8 Hz, 1H), 3.87 (t, J = 5.1 Hz, 2H), 3.54 (dd, J = 6.3, 3.9 Hz, 2H), 3.19 (t, J = 5.2 Hz, 2H), 3.06 (dd, J = 6.1, 3.9 Hz, 2H). ^{13}C NMR (101 MHz, DMSO-d_6) δ 166.05, 158.14, 147.14, 146.04, 146.00, 145.96, 145.92, 137.13, 135.62, 135.59, 135.55, 135.52, 127.96, 126.48, 126.15, 125.83, 125.51, 125.25, 124.36,

123.85, 122.54, 120.73, 112.68, 101.19, 99.16, 51.33, 50.94, 47.11, 42.22.

Scheme 3. General Preparation of Compound Z6*[a]

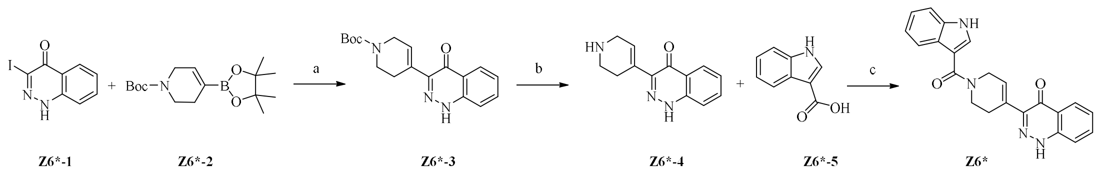

[a]Reagents and conditions: (a) Pd(dppf)Cl_2, K_2CO_3, 1,4-dioxane/H_2O = 10/1 (*v*/*v*), 80 ºC, 2h; (b) HCl in 1,4-dioxane, rt, 1h; (c) DIPEA, HATU, DCM, rt, 5h.

General Preparation of Compound Z6. Step a: A solution of **Z6*-1** in 1,4-dioxane and H_2O was treated with K_2CO_3, **Z6*-2**, and Pd(dppf)Cl_2 (0.02 mmol). The reaction mixture was placed under nitrogen displacement and heated to 80°C for 8 h. Upon completion of the reaction, the mixture was extracted three times with water and ethyl acetate. The combined organic phases were dried over anhydrous sodium sulfate, filtered, and then evaporated to dryness. The resulting residue was purified by silica gel chromatography to give **Z6*-3**.

Step b: To a solution of **Z6*-3** in MeOH and HCl in 1,4-dioxane. The mixture was stirred at room temperature for 1h. After completion, it was concentrated under reduced pressure to give the **Z6*-4**.

Step c: To a solution of compound **Z6*-4** in DCM was added **Z6*-5**, HATU, and DIPEA . The mixture was stirred at room temperature for 5 h. After completion, the mixture was added water and extracted with DCM. The combined organic phase was washed with brine, deied over anhydrous sodium sulfate and concentrated under reduced pressure. The residue was purified by chromatography over silica gel to give the compound **Z6***.

^{1}H NMR (400 MHz, DMSO-d_6) δ 13.39 (s, 2H), 11.30 (s, 1H), 8.13 (d, J = 8.2 Hz,

1H), 7.78 (ddd, J = 8.5, 6.9, 1.5 Hz, 1H), 7.61 (dd, J = 8.3, 5.9 Hz, 2H), 7.52 (d, J = 1.3 Hz, 1H), 7.48 (t, J = 2.7 Hz, 1H), 7.42 (ddd, J = 8.1, 6.9, 1.0 Hz, 1H), 7.10 (dd, J = 8.1, 1.5 Hz, 1H), 6.50 (ddd, J = 3.0, 1.9, 0.9 Hz, 1H), 4.30 (s, 2H), 3.71 (s, 2H), 2.66 (s, 2H). ^{13}C NMR (101 MHz, DMSO-d_6) δ 170.07, 144.45, 140.62, 135.54, 133.94, 131.62, 131.59, 129.11, 128.98, 127.83, 125.10, 125.10, 123.51, 120.24, 120.24, 116.80, 116.79, 113.66, 111.21, 101.70, 101.70.

Scheme 4. General Preparation of Compound Y1*[a]

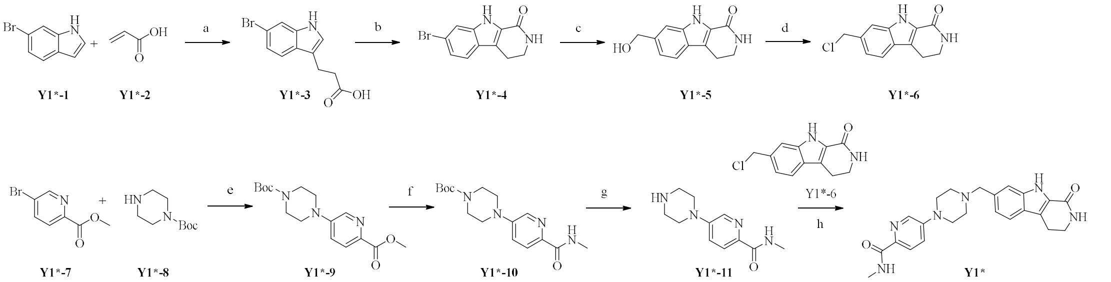

[a]Reagents and conditions: (a) AcOH, 100 ºC, 16h; (b) diphenylphosphoryl azide, triethylamine, toluene, 100 ºC, 3h; (c) (Tributylstannyl) methanol, SPhos Pd G2, 1,4-dioxane, 100 ºC, 12h; (d) DCM, DMF, $SOCl_2$, 0 ºC, 1h; (e) RuPhos Pd G3, Cs_2CO_3, 1,4-dioxane, 100 ºC, 12h; (f) $CH_3NH_2·H_2O$, MeOH, rt, 12h; (g) HCl in 1,4-dioxane, rt, 1h; (h) KI, DIPEA, MeCN, 80 ºC 2h.

General Preparation of Compound Y1*. Step a: Weigh the compounds **Y1*-1**, **Y1*-2** add acetic acid, temperature 100 ºC stirring 16h, after the reaction first concentrated off most of the acetic acid, then add ethyl acetate and sodium bicarbonate solution, the organic phase was washed with saturated brine, anhydrous sodium sulfate drying, filtration, spinning dry, the resulting crude product by column chromatography purification of the compounds **Y1*-3**.

Step b: Compound **Y1*-3**, add triethylamine, toluene, add diphenylphosphoryl azide, temperature 100 ºC stirring 3h, after the reaction is concentrated dry, then add boron trifluoride ethyl ether, room temperature stirring for 6h, then the reaction solution was poured into ice water, add ethyl acetate extraction partition, the organic phase was washed with saturated sodium bicarbonate, saturated brine, anhydrous sodium sulfate

drying, spin drying of the resulting crude product by column chromatography purification of the compound **Y1*-4**.

Step c: Weighing out **Y1*-4**, (Tributylstannyl) methanol, and Sphos Pd G2, we added of 1,4-dioxane under nitrogen displacement, and then heated the reaction to 100 °C for 12 h. Upon completion, the reaction mixture was extracted three times with water and ethyl acetate. The combined organic phases were dried over anhydrous sodium sulfate, filtered, and then concentrated. The resulting residue was purified by silica gel chromatography to give **Y1*-5**.

Step d: To a suspension of compound **Y1*-5** in DCM was added DMF and $SOCl_2$ at 0 °C for 1 h. After completion, the mixture was concentrated to give the compound **Y1*-6**.

Step e: A solution containing **Y1*-7** and of **Y1*-8** in 1,4-dioxane was treated with Cs_2CO_3 and RuPhos Pd G3. After displacing nitrogen, the reaction mixture was heated to 100°C for 12 h. Upon completion of the reaction, the mixture was subjected to extraction by adding water and ethyl acetate three times. The combined organic phases were then dried using anhydrous sodium sulfate, filtered, and evaporated to dryness. The resulting residue was purified by silica gel chromatography to give **Y1*-9**.

Step f: Compound **Y1*-9** was dissolved in MeOH and aqueous methylamine and stirred at room temperature for 12 h. At the end of the reaction, it was concentrated under reduced pressure to give the compound **Y1*-10**.

Step g: To a solution of **Y1*-10** in MeOH and HCl in 1,4-dioxane. The mixture was stirred at room temperature for 1h. After completion, it was concentrated under reduced pressure to give the **Y1*-11**.

Step h: To a suspension of **Y1*-6**, **Y1*-11**, KI and DIPEA in CH_3CN. The mixture was stirred at 80 °C for 2 h. After completion, the solvent was removed under vacuum. Finally, the crude product was purified by silica gel chromatography to obtain compound **Y1***. ^1H NMR (400 MHz, DMSO-d_6) δ 11.56 (s, 1H), 8.41 (q, J = 4.8 Hz,

1H), 8.25 (d, J = 2.8 Hz, 1H), 7.83 (d, J = 8.7 Hz, 1H), 7.54 (d, J = 8.4 Hz, 2H), 7.37 (d, J = 8.5 Hz, 2H), 7.09 – 7.02 (m, 1H), 3.60 (s, 2H), 3.51 (td, J = 6.9, 2.4 Hz, 2H), 3.31 (t, J = 4.7 Hz, 4H), 2.91 (t, J = 6.9 Hz, 2H), 2.79 (d, J = 4.8 Hz, 3H), 2.55 – 2.51 (m, 4H). ^{13}C NMR (101 MHz, DMSO-d_6) δ 165.10, 162.37, 148.52, 140.24, 137.69, 135.52, 134.43, 127.75, 124.63, 122.66, 121.55, 121.29, 120.30, 118.64, 113.13, 63.10, 52.62, 47.15, 26.30, 20.87.

Scheme 5. General Preparation of Compound Y2^a

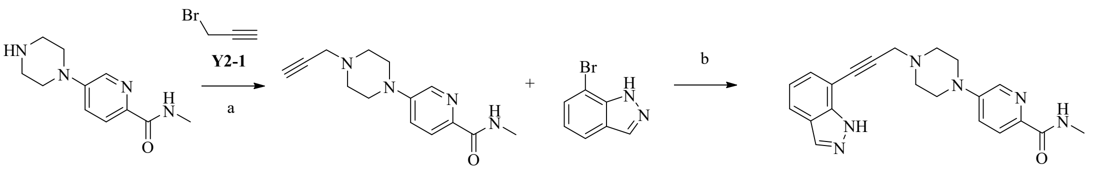

Y1*-11 Y2-2 Y2-3 Y2

^{a}Reagents and conditions: (a) TEA, DCM, rt, 12h; (b) CuI, $Pd(PPh_3)_2Cl_2$, 1,4-dioxane, 100 ºC, 12h.

General Preparation of Compound Y2. Step 1: Compounds **Y1*-11, Y2-1** were dissolved in DCM, then triethylamine was added and stirred overnight at room temperature. After the reaction was completed, an aqueous layer was added and the crude product obtained by spin-drying the organic phase was purified by column chromatography to give compound **Y2-2.**

Step 2: A solution containing **Y2-2** and of **Y2-3** in 1,4-dioxane was treated with CuI and $Pd(PPh_3)_2Cl_2$. After displacing nitrogen, the reaction mixture was heated to 100°C for 12 h. Upon completion of the reaction, the mixture was subjected to extraction by adding water and ethyl acetate three times. The combined organic phases were then dried using anhydrous sodium sulfate, filtered, and evaporated to dryness. The resulting residue was purified by silica gel chromatography to give **Y2**. ^{1}H NMR (400 MHz, DMSO-d_6) δ 13.45 (s, 1H), 8.43 (q, J = 4.8 Hz, 1H), 8.30 (d, J = 2.9 Hz, 1H), 8.17 (s, 1H), 7.85 (dd, J = 8.8, 2.5 Hz, 1H), 7.80 (d, J = 8.1 Hz, 1H), 7.45 (d, J = 7.1 Hz, 1H), 7.41 (dd, J = 9.0, 2.8 Hz, 1H), 7.15 – 7.06 (m, 1H), 3.71 (s, 2H), 3.34 (t, J =

5.2 Hz, 2H), 2.80 (d, J = 4.8 Hz, 3H), 2.76 (t, J = 5.0 Hz, 4H), 2.60 (t, J = 5.0 Hz, 2H). ^{13}C NMR (101 MHz, DMSO-d_6) δ 165.08, 148.52, 148.42, 140.39, 140.36, 135.71, 135.64, 134.91, 130.00, 123.49, 122.66, 121.83, 121.45, 121.41, 120.88, 90.55, 81.24, 75.04, 69.64, 51.54, 51.20, 47.65, 47.11, 47.03, 46.89, 26.30.

Scheme 6. General Preparation of Compound Y4[a]

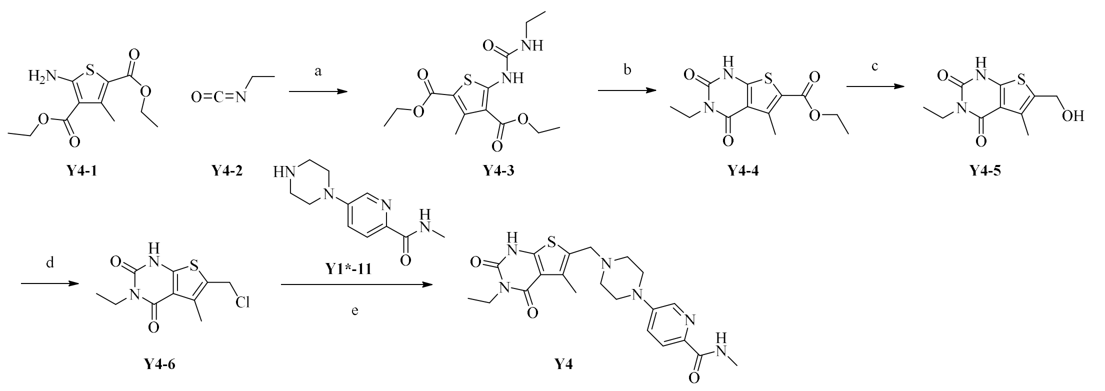

[a]Reagents and conditions: (a) pyridine, 110 ºC, 12h; (b) NaOEt, EtOH, 30 ºC, 1h; (c) $LiAlH_4$, THF, 0 ºC, 1h; (d) DCM, DMF, $SOCl_2$, 0 ºC, 1h; (e) KI, DIPEA, MeCN, 80 ºC 2h.

General Preparation of Compound Y4. Step a: A solution containing **Y4-1** an d of **Y4-2** in pyridine heated to 110°C for 12 h. Upon completion of the reacti on, the mixture was subjected to extraction by adding water and DCM three ti mes. The combined organic phases were then dried using anhydrous sodium su lfate, filtered, and evaporated to dryness. The resulting residue was purified by silica gel chromatography to give **Y4-3**.

Step b: A solution containing **Y4-3** in EtOH heated to 30°C for 1 h. Upon completion of the reaction, the mixture was subjected to extraction by adding water and DCM three times. The combined organic phases were then dried using anhydrous sodium sulfate, filtered, and evaporated to dryness. The resulting residue was purified by silica gel chromatography to give **Y4-4**.

Step c: To a solution of compound **Y4-4** in THF was added $LiAlH_4$ at 0 °C for 1 h.

After completion, the mixture was added DCM and quenched with water. The mixture was stirred at room temperature for 1 h. The mixture was dried over anhydrous sodium sulfate and filtered, and filter cake was washed with MeOH. The filtrate was concentrated under reduced pressure to give the compound **Y4-5.**

Step d: To a suspension of compound **Y4-5** in DCM was added DMF and $SOCl_2$ at 0 °C for 1 h. After completion, the mixture was concentrated to give the compound **Y4-6.**

Step e: To a suspension of **Y4-6**, **Y1*-11**, KI and DIPEA in CH_3CN. The mixture was stirred at 80 °C for 2 h. After completion, the solvent was removed under vacuum. Finally, the crude product was purified by silica gel chromatography to obtain compound **Y4**. ^{1}H NMR (400 MHz, DMSO-d_6) δ 12.27 (s, 1H), 8.44 (q, J = 4.8 Hz, 1H), 8.32 (d, J = 2.9 Hz, 1H), 7.87 (d, J = 8.7 Hz, 1H), 7.46 (dd, J = 8.9, 2.9 Hz, 1H), 4.85 – 3.93 (m, 4H), 3.87 (q, J = 6.9 Hz, 2H), 3.36 – 3.05 (m, 6H), 2.79 (d, J = 4.8 Hz, 3H), 2.45 (s, 3H), 1.12 (t, J = 7.0 Hz, 3H). ^{13}C NMR (101 MHz, DMSO-d_6) δ 164.92, 164.92, 162.65, 159.22, 153.98, 150.33, 150.33, 136.14, 122.72, 122.72, 122.14, 113.32, 113.32, 35.28, 26.33, 14.21, 13.38.

Scheme 7. General Preparation of Compound Y5^a

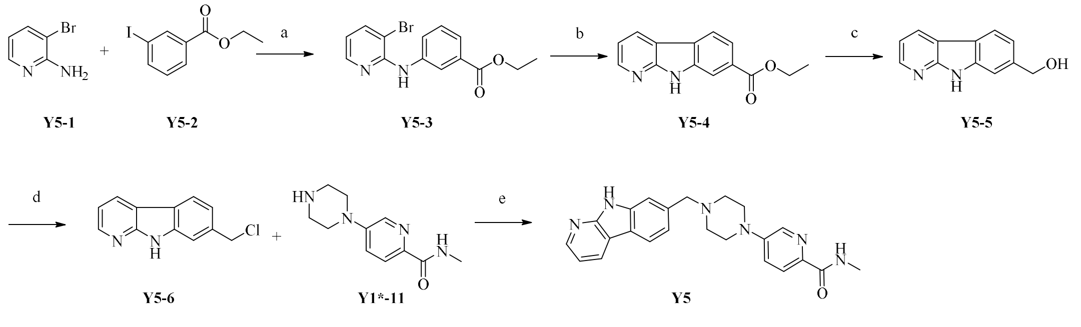

^{a}Reagents and conditions: (a) $Pd(OAc)_2$, Xantphos, Cs_2CO_3, C_7H_8O, 130 ºC, 12h; (b) $Pd(OAc)_2$, DCHPB, DBU, DMA, 130 ºC, 12h; (c) $LiAlH_4$, THF, 0 ºC, 1h; (d) DCM, DMF, $SOCl_2$, 0 ºC, 1h; (e) KI, DIPEA, MeCN, 80 ºC 2h.

General Preparation of Compound Y5. Step a: A solution containing **Y5-1** and of **Y5-2** in C_7H_8O was treated with Cs_2CO_3, $Pd(OAc)_2$, and Xantphos. After displacing nitrogen, the reaction mixture was heated to 130°C for 12 h. Upon completion of the reaction, the mixture was subjected to extraction by adding water and ethyl acetate three times. The combined organic phases were then dried using anhydrous sodium sulfate, filtered, and evaporated to dryness. The resulting residue was purified by silica gel chromatography to give **Y5-3**.

Step b: A solution containing **Y5-3** in DMA was treated with DBU, $Pd(OAc)_2$,and DCHPB. After displacing nitrogen, the reaction mixture was heated to 130°C for 12 h. Upon completion of the reaction, the mixture was subjected to extraction by adding water and ethyl acetate three times. The combined organic phases were then dried using anhydrous sodium sulfate, filtered, and evaporated to dryness. The resulting residue was purified by silica gel chromatography to give **Y5-4**.

Step c: To a solution of compound **Y5-4** in THF was added $LiAlH_4$ at 0 °C for 1 h. After completion, the mixture was added DCM and quenched with water. The mixture was stirred at room temperature for 1 h. The mixture was dried over anhydrous sodium sulfate and filtered, and filter cake was washed with MeOH. The filtrate was concentrated under reduced pressure to give the compound **Y5-5**.

Step d: To a suspension of compound **Y5-5** in DCM was added DMF and $SOCl_2$ at 0 °C for 1 h. After completion, the mixture was concentrated to give the compound **Y5-6**.

Step e: To a suspension of **Y5-6**, **Y1*-11**, KI and DIPEA in CH_3CN. The mixture was stirred at 80 °C for 2 h. After completion, the solvent was removed under vacuum. Finally, the crude product was purified by silica gel chromatography to obtain compound **Y5**. ^{1}H NMR (400 MHz, DMSO-d_6) δ 11.77 (s, 1H), 8.84 (s, 2H), 8.48 (d, J = 7.7 Hz, 1H), 8.40 (d, J = 5.0 Hz, 2H), 8.28 (d, J = 2.9 Hz, 1H), 8.12 (d, J = 7.8 Hz, 1H), 7.83 (d, J = 8.8 Hz, 1H), 7.52 – 7.48 (m, 1H), 7.40 (dd, J = 9.0, 2.9 Hz, 1H), 7.20 (dd, J = 7.7, 4.9 Hz, 2H), 3.61 (pd, J = 6.6, 3.3 Hz, 4H), 3.20 – 3.08 (m, 4H), 2.78 (d,

J = 4.8 Hz, 3H), 2.59 (s, 2H). ^{13}C NMR (101 MHz, DMSO-d_6) δ 163.42, 152.59, 152.59, 147.38, 146.87, 146.35, 141.09, 139.45, 139.45, 129.09, 128.72, 121.41, 120.67, 115.61, 115.61, 115.46, 54.04, 49.67, 42.30, 26.56, 18.57, 17.23, 12.99.

Scheme 8. General Preparation of Compound Y6^a

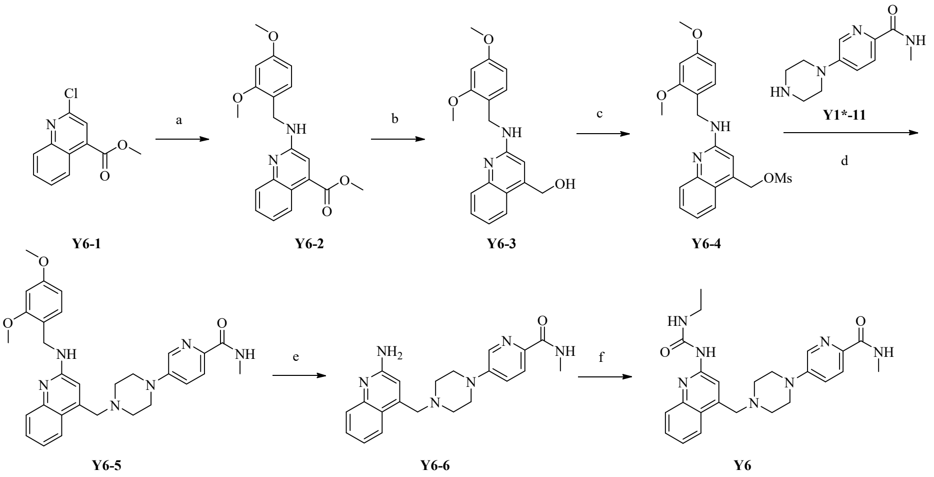

^{a}Reagents and conditions: (a) TEA, NMP, rt, 12h; (b) $LiAlH_4$, THF, 0 ºC, 1h; (c) TEA, MsCl, DCM, rt, 3h; (d) KI, DIPEA, MeCN, 80 ºC 2h; (e) H_2SO_4, DCM, rt, 1h; (f) TEA, DMF, rt, 8h.

General Preparation of Compound Y6. Step a: Compounds **Y6-1**, 2,4-Dimethoxybenzylamine were dissolved in NMP, then TEA was added and stirred overnight at room temperature. After the reaction was completed, water and EA were added to separate the layers, and the crude product obtained by spin-drying the organic phase was purified by column chromatography to give compound **Y6-2**.

Step b: To a solution of compound **Y6-2** in THF was added $LiAlH_4$ at 0 °C for 1 h. After completion, the mixture was added DCM and quenched with water. The mixture was stirred at room temperature for 1 h. The mixture was dried over anhydrous sodium sulfate and filtered, and filter cake was washed with MeOH. The filtrate was concentrated under reduced pressure to give the compound **Y6-3**.

Step c: Compound **Y6-3**, MsCl and TEA were dissolved in DCM and stirred at room temperature for 3 h. After the reaction was monitored for completeness by TLC, the

reaction was quenched by the addition of saturated sodium bicarbonate, and the layers were separated by the addition of ethyl acetate and water in a partition funnel. Extraction was carried out using ethyl acetate for 2-3 times, and the organic phases were combined before being washed with water, saturated saline water, dried over anhydrous sodium sulfate, filtered, and spun dry, respectively. The resulting crude product was purified by column chromatography to give compound **Y6-4**.

Step d: To a suspension of **Y6-4**, **Y1*-11**, KI and DIPEA in CH_3CN. The mixture was stirred at 80 °C for 2 h. After completion, the solvent was removed under vacuum. Finally, the crude product was purified by silica gel chromatography to obtain compound **Y6-5**.

Step e: Compound **Y6-5** was dissolved in 5 ml of dichloromethane, followed by the addition of 0.5 ml of concentrated sulfuric acid and stirred at room temperature for 0.5 h. After the reaction was monitored for completeness by TLC, the pH was adjusted to a weak base by the addition of saturated sodium bicarbonate, and ethyl acetate and water were added, and the layers were partitioned in a partition funnel. Extracted with ethyl acetate for 2-3 times, the organic phases were combined and then washed with water, washed with saturated saline water, dried with anhydrous sodium sulfate, filtered and spun-dried, respectively, to obtain the crude compound **Y6-6**.

Step f: **Y6-6**, ethyl isocyanate, and TEA were dissolved in DMF and stirred at room temperature for 8 h. After the reaction was complete as monitored by TLC, ethyl acetate and water were added and partitioned in a split funnel. It was extracted 2-3 times with ethyl acetate, and the organic phases were combined before being washed with water, washed with saturated brine, dried with anhydrous sodium sulfate, filtered and spun dry, respectively. The resulting crude product was purified by column chromatography to give compound **Y6**. ^{1}H NMR (400 MHz, DMSO-d_6) δ 9.65 (s, 1H), 9.38 (s, 1H), 8.41 (q, J = 4.8 Hz, 1H), 8.28 (d, J = 2.9 Hz, 1H), 8.14 – 8.07 (m, 1H), 7.87 – 7.77 (m, 2H), 7.65 (ddd, J = 8.3, 6.8, 1.4 Hz, 1H), 7.48 – 7.37 (m, 3H), 3.93 (s, 2H), 3.38 (d, J = 5.4 Hz, 4H), 3.34 – 3.24 (m, 2H), 2.78 (d, J = 4.8 Hz, 3H), 2.65 (t, J

= 4.9 Hz, 4H), 1.19 (t, J = 7.2 Hz, 3H). ^{13}C NMR (101 MHz, DMSO-d_6) δ 165.07, 155.22, 153.04, 148.52, 146.21, 146.03, 140.37, 135.55, 130.12, 127.30, 124.63, 124.35, 123.86, 122.67, 121.39, 113.47, 58.71, 52.90, 47.24, 34.42, 26.30, 15.79, 0.58.

Scheme 9. General Preparation of Compound R1*[a]

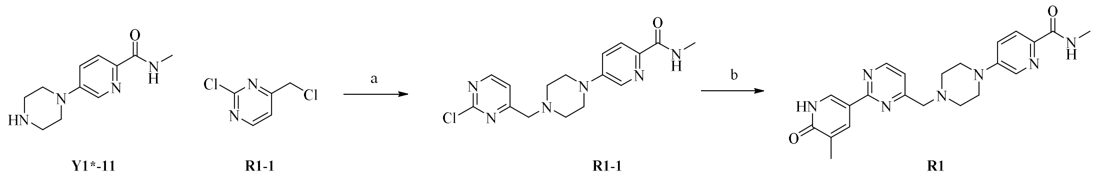

[a]Reagents and conditions: (a) KI, DIPEA, MeCN, 80 ºC 2h; (b) Pd(dppf)Cl_2, K_2CO_3, 1,4-dioxane/H_2O = 10/1 (*v*/*v*), 80 ºC, 2h

General Preparation of Compound R1. Step a: To a suspension of **Y1*-11**, **R1-1**, KI and DIPEA in CH_3CN. The mixture was stirred at 80 °C for 2 h. After completion, the solvent was removed under vacuum. Finally, the crude product was purified by silica gel chromatography to obtain compound **R1-2**.

Step b: A solution of **R1-2** in 1,4-dioxane and H_2O was treated with K_2CO_3, 3-methyl-5-(4,4,5,5-tetramethyl-1,3,2-dioxaborolan-2-yl)pyridin-2(1H)-one, and Pd(dppf)Cl_2 (0.02 mmol). The reaction mixture was placed under nitrogen displacement and heated to 80°C for 8 h. Upon completion of the reaction, the mixture was extracted three times with water and ethyl acetate. The combined organic phases were dried over anhydrous sodium sulfate, filtered, and then evaporated to dryness. The resulting residue was purified by silica gel chromatography to give **R1**. ^{1}H NMR (400 MHz, DMSO-d_6) δ 12.06 (s, 1H), 8.85 (d, J = 5.1 Hz, 1H), 8.46 (q, J = 4.8 Hz, 1H), 8.36 (dd, J = 11.3, 2.7 Hz, 2H), 8.30 – 8.25 (m, 1H), 7.89 (d, J = 8.7 Hz, 1H), 7.49 (dd, J = 8.8, 2.9 Hz, 1H), 7.42 (d, J = 5.0 Hz, 1H), 4.47 (s, 2H), 3.92 – 3.52 (m, 8H), 2.80 (d, J = 4.9 Hz, 3H), 2.09 (s, 3H). ^{13}C NMR (101 MHz, DMSO) δ 164.93, 163.41, 161.98, 158.86, 147.52, 141.22, 136.17, 136.07, 135.37, 128.73, 122.73, 122.09, 118.19, 115.54, 51.84, 26.33, 17.10.

Scheme 10. General Preparation of Compound R2[a]

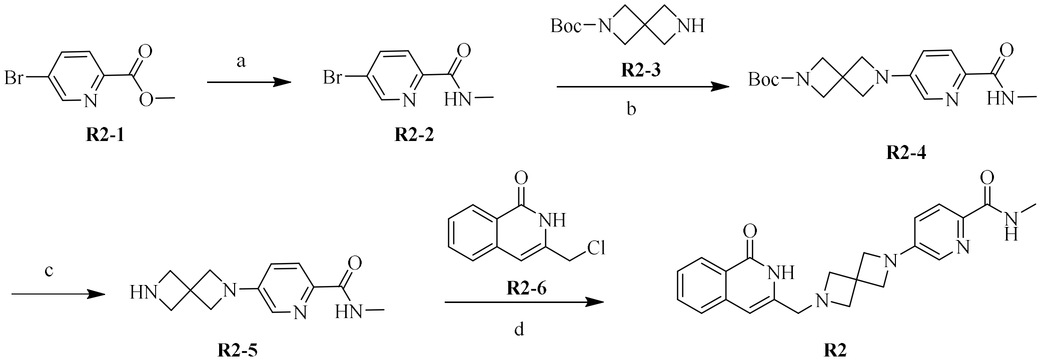

[a]Reagents and conditions: (a) $CH_3NH_2{\cdot}H_2O$, MeOH, rt, 12h; (b) RuPhos Pd G3, Cs_2CO_3, 1,4-dioxane, 100 °C, 12h; (c) HCl in 1,4-dioxane, rt, 1h; (d) KI, DIPEA, MeCN, 80 °C 2h.

General Preparation of Compound R2. Step a: Add methanol to compound **R2-1** and, at room temperature, slowly add an aqueous solution of methylamine. Continue stirring and reacting for 12 hours. After the reaction is complete, concentrate under reduced pressure to obtain compound **R2-2**.

Step b: A solution containing **R2-2** and of **R2-3** in 1,4-dioxane was treated with Cs_2CO_3 and RuPhos Pd G3. After displacing nitrogen, the reaction mixture was heated to 100°C for 12 h. Upon completion of the reaction, the mixture was subjected to extraction by adding water and ethyl acetate three times. The combined organic phases were then dried using anhydrous sodium sulfate, filtered, and evaporated to dryness. The resulting residue was purified by silica gel chromatography to give **R2-4**.

Step c: To a solution of **R2-4** in MeOH and HCl in 1,4-dioxane. The mixture was stirred at room temperature for 1h. After completion, it was concentrated under reduced pressure to give the **R2-5**.

Step d: To a suspension of **R2-5**, **R2-6**, KI and DIPEA in CH_3CN. The mixture was stirred at 80 °C for 2 h. After completion, the solvent was removed under vacuum. Finally, the crude product was purified by silica gel chromatography to obtain compound **R2**. ^{1}H NMR (400 MHz, Methanol-*d*4) δ 8.26 (dd, *J* = 8.2, 1.3 Hz, 1H), 8.04 (d, *J* = 2.9 Hz, 1H), 7.83 (d, *J* = 8.6 Hz, 1H), 7.66 (ddd, *J* = 8.3, 7.0, 1.4 Hz, 1H),

7.56 (dd, J = 8.1, 1.2 Hz, 1H), 7.45 (ddd, J = 8.1, 6.9, 1.2 Hz, 1H), 7.04 (dd, J = 8.6, 2.8 Hz, 1H), 6.51 (s, 1H), 3.88 (s, 2H), 3.57 (s, 2H), 3.47 (s, 2H), 3.28 – 3.17 (m, 4H), 2.93 (s, 3H). ^{13}C NMR (101 MHz, MeOD-d_4) δ 166.87, 163.90, 147.75, 147.70, 138.24, 137.53, 137.26, 134.23, 134.18, 132.68, 126.54, 126.34, 126.16, 124.70, 122.77, 116.68, 116.53, 105.24, 60.75, 59.14, 58.58, 58.53, 45.66, 40.67, 39.80, 24.96.

Biological Assay Methods

PARP1 inhibitory activity assay. The histone coated 384-well plates were prepared by adding 25 μL histone solution to each well and incubating overnight at 4°C. Prepared PBST buffer, blocking buffer, and assay buffer. Washed the histone coated 384-well plate 3 times using PBST buffer solution. It was blocked with 50 μL blocking buffer at room temperature for 1 h. The plate was washed 3 times using PBST buffer. 2000x compounds were prepared in the source plate, and 50nl compound were transferred from the source plate to the 96-well intermediate plate with 19.95ul assay buffer, shaken, and centrifuged at 1000rpm for 1 min. 5 μL DMSO/compound was then transferred to each well. The PARP1 and DNA mixture was incubated for 10 min at 25°C, followed by the addition of 10 μL PARP1 and DNA (except min control), incubated with the compound for 10 min at RT, and 10 μL DNA was added to the minimal control of assay plate. Added 10 μL of 2.5x NAD+ to each well and incubated for 60 min at 25°C. The plate was washed 3 times with PBST buffer and then assayed. Added 20 μL of anti-Poly/Mono-ADP Ribose Rabbit mAb. Incubated for 1.5 h at RT and washed the plate 3 times with PBST buffer. Then added, 20 μL of diluted (1:2000 in Blocking buffer) anti-rabbit IgG, HRP-linked Antibody. Incubated for 1 h at RT and washed the plate 3 times using PBST buffer. Read chemiluminescence on Envision immediately after adding 25 μL of Femto-ECL Substrate A and Femto-ECL Substrate B (1:1) mix.

The IC_{50} values were calculated by analyzing the concentration-response curves.

Fit the data to obtain inhibition values using equation (1)

Equation (1): inh %=(Max-Signal)/ (Max-Min)*100

Fit the data to obtain IC_{50} values using equation (2)

Equation (2): Y=Bottom + (Top-Bottom)/(1+(IC_{50}/X)*HillSlope)

Y is %inhibition and X is compound concentration.

PARP2 inhibitory activity assay. The histone coated 384-well plates were prepared by adding 25 μL histone solution to each well and incubating overnight at 4°C. Prepared PBST buffer, blocking buffer, and assay buffer. Washed the histone coated 384-well plate 3 times using PBST buffer solution. It was blocked with 50 μL blocking buffer at room temperature for 1 h. The plate was washed 3 times using PBST buffer. 2000x compounds were prepared in the source plate, and 50nl compound were transferred from the source plate to the 96-well intermediate plate with 19.95ul assay buffer, shaken, and centrifuged at 1000rpm for 1 min. 5 μL DMSO/compound was then transferred to each well. The PARP2 mixture was incubated for 10 min at 25°C, followed by the addition of 10 μL PARP2 (except min control), incubated with the compound for 10 min at RT, and 10 μL assay buffer was added to the minimal control of assay plate. Added 10 μL of 2.5x Biotin-NAD+ to each well and incubated for 90 min at 25°C. The plate was washed 3 times with PBST buffer and then assayed. Added 25 μL of Stre-HRP. Incubated for 1 h at RT and washed the plate 3 times with PBST buffer. Read chemiluminescence on Envision immediately after adding 25 μL of Femto-ECL Substrate A and Femto-ECL Substrate B (1:1) mix. The IC_{50} values were calculated by analyzing the concentration-response curves.

Fit the data in Excel to obtain inhibition values using equation (1)

Equation (1): inh %=(Max-Signal)/ (Max-Min)*100

Fit the data in XL-Fit to obtain IC_{50} values using equation (2)

Equation (2): Y=Bottom + (Top-Bottom)/(1+(IC_{50}/X)*HillSlope)

Y is %inhibition and X is compound concentration.

^{1}H NMR Spectrum of **Z2***

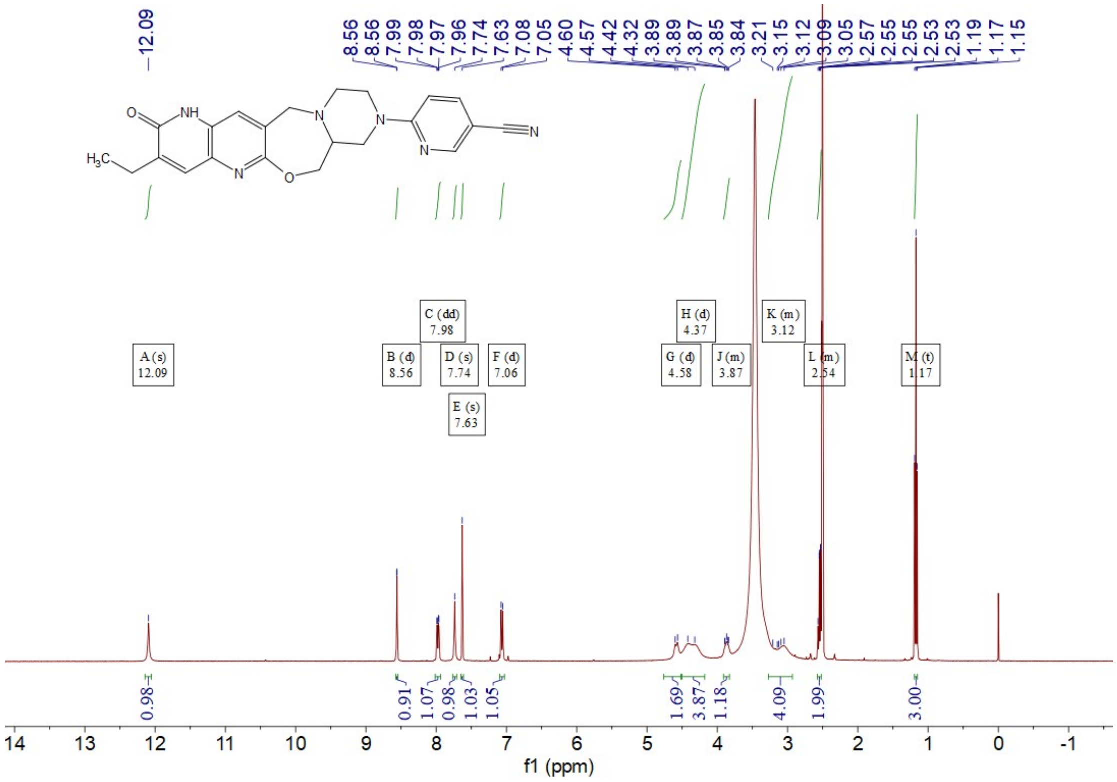

^{13}C NMR Spectrum of **Z2***

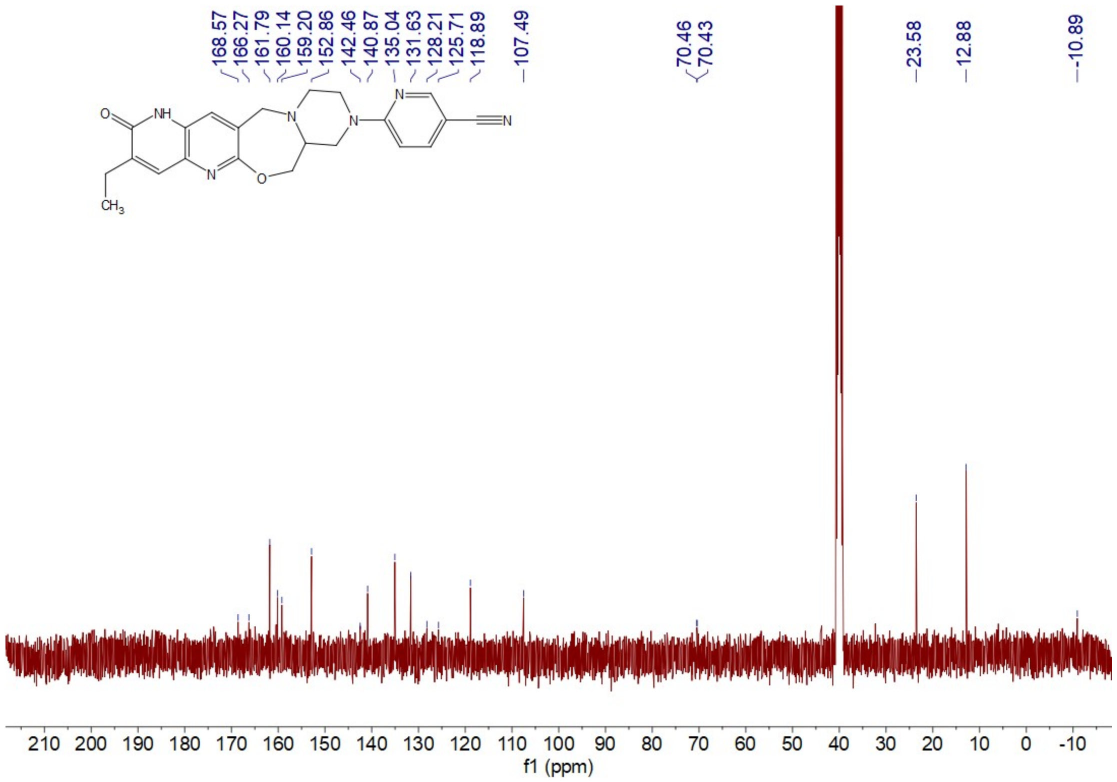

^{1}H NMR Spectrum of **Z5**

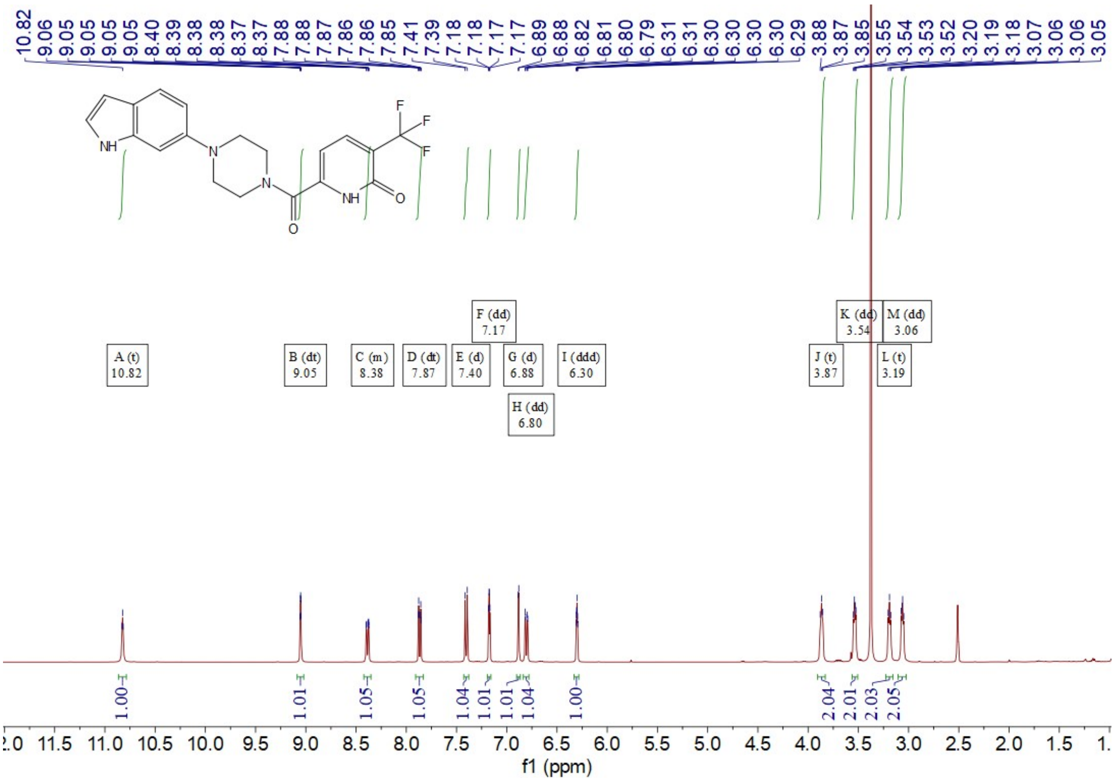

^{13}C NMR Spectrum of **Z5**

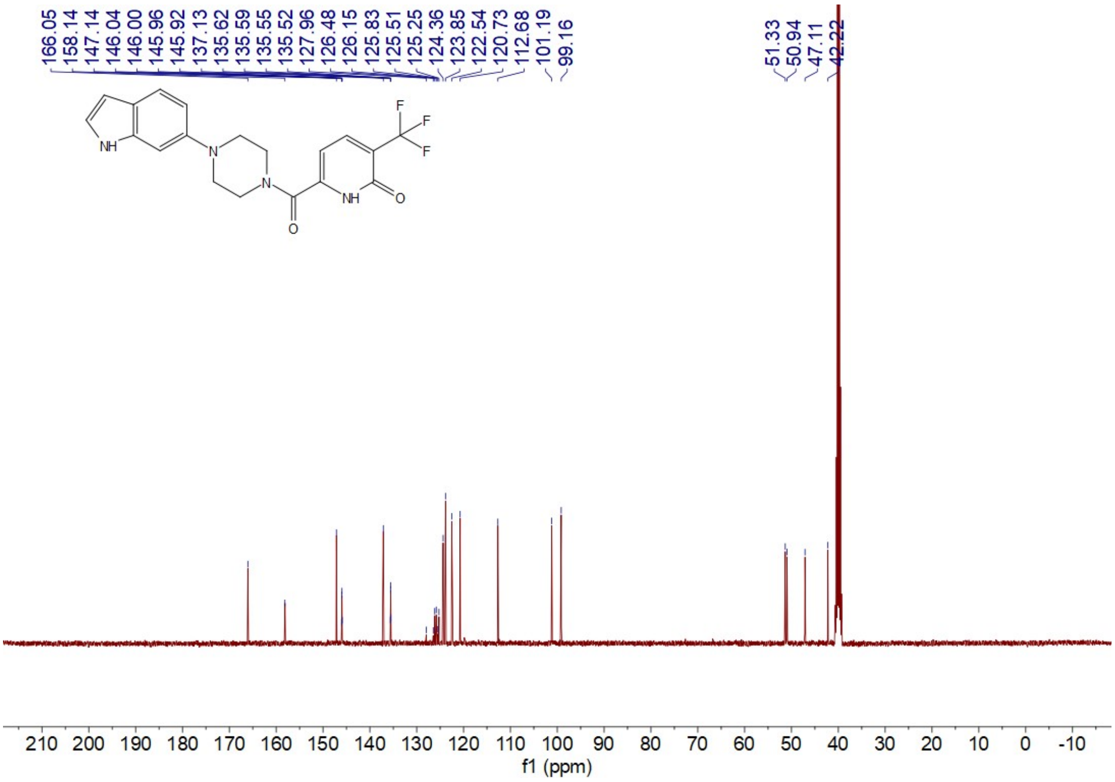

^{1}H NMR Spectrum of **Z6***

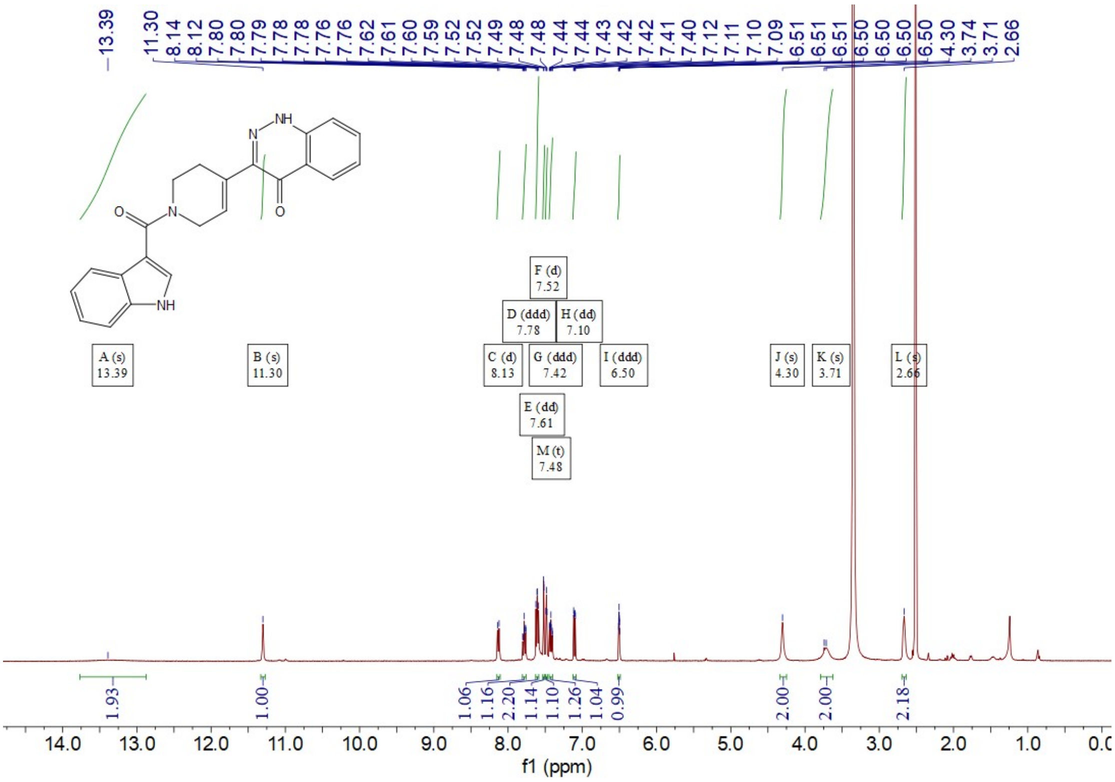

^{13}C NMR Spectrum of **Z6***

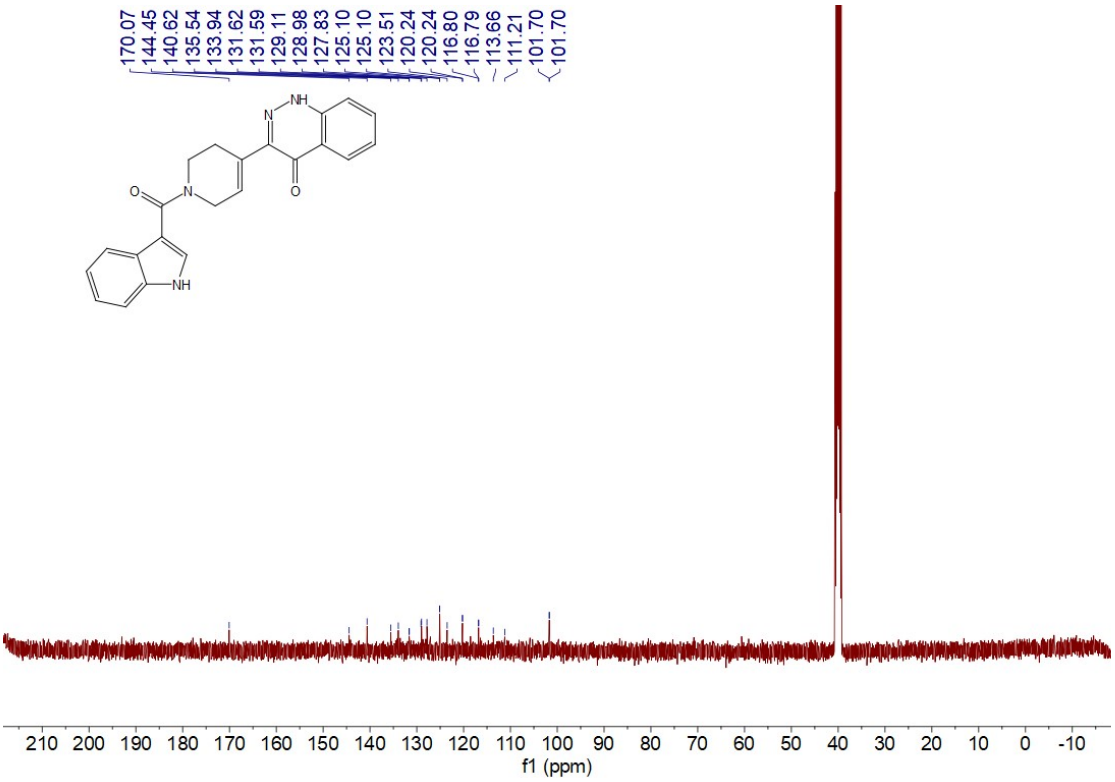

^{1}H NMR Spectrum of **Y1***

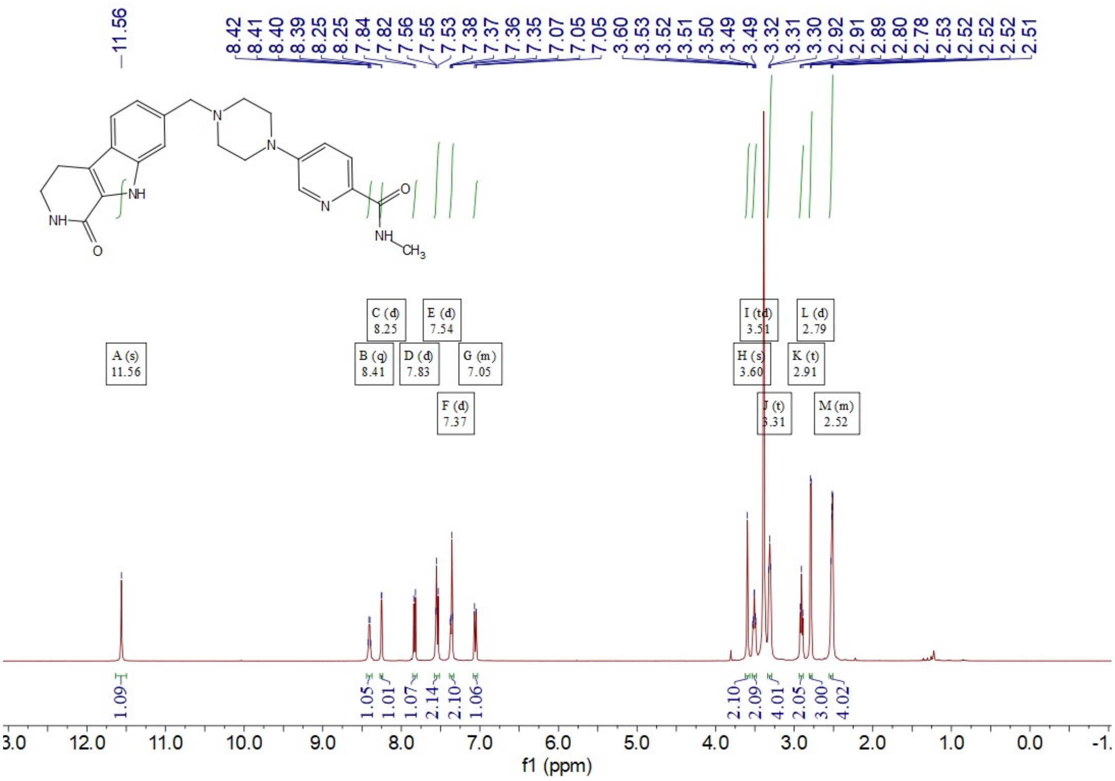

^{13}C NMR Spectrum of **Y1***

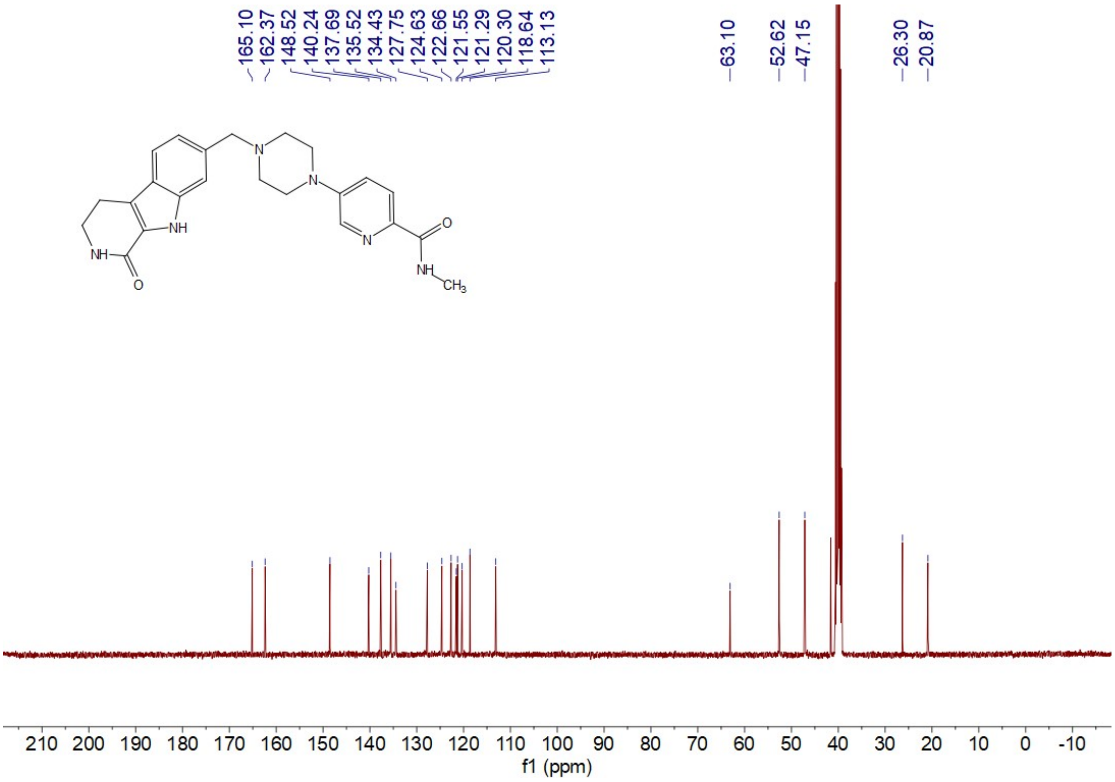

^{1}H NMR Spectrum of **Y2**

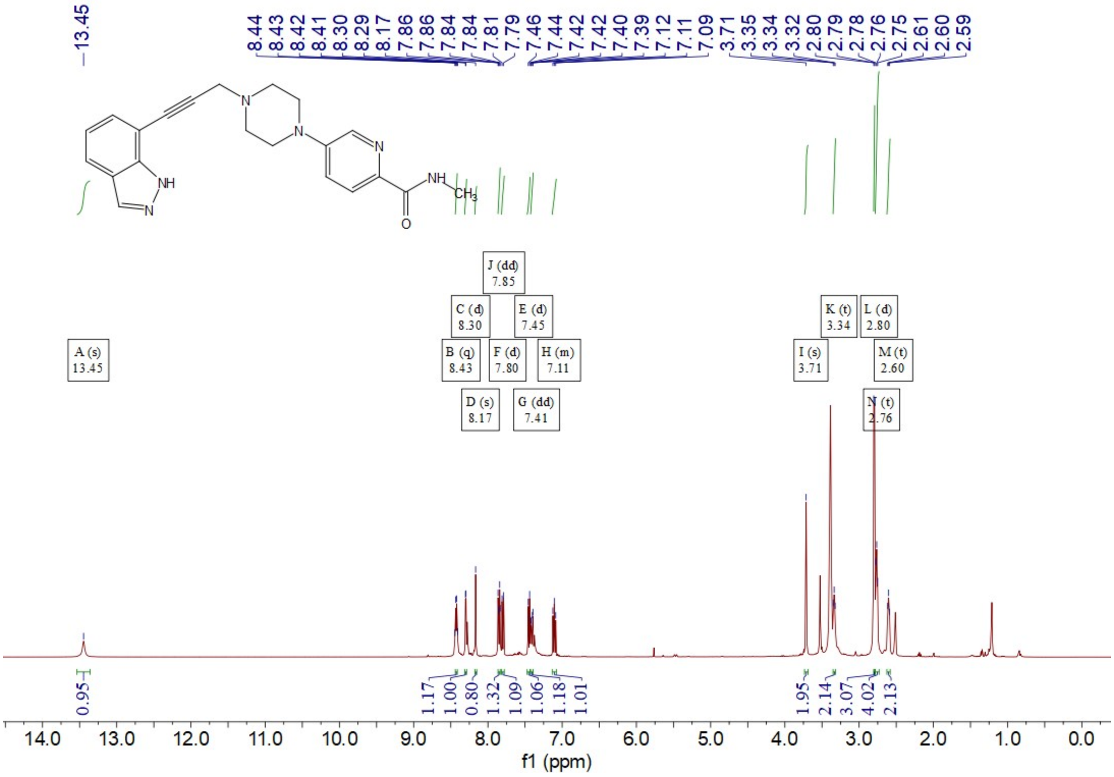

^{13}C NMR Spectrum of **Y2**

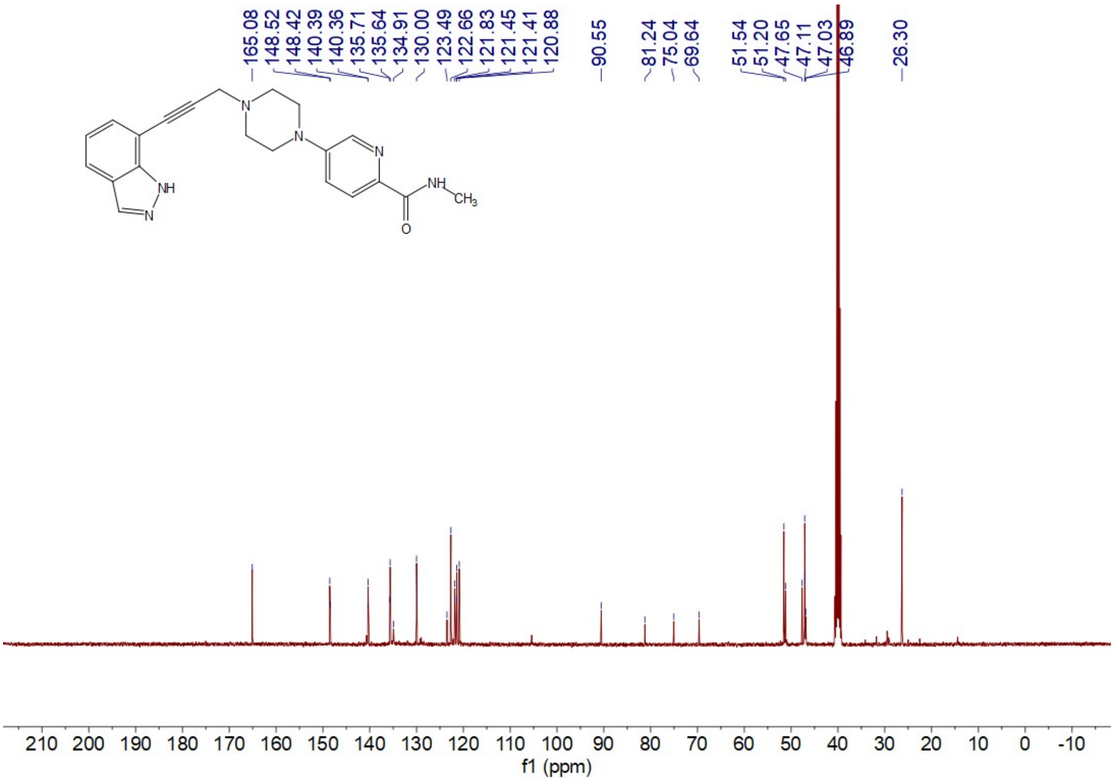

^{1}H NMR Spectrum of **Y4**

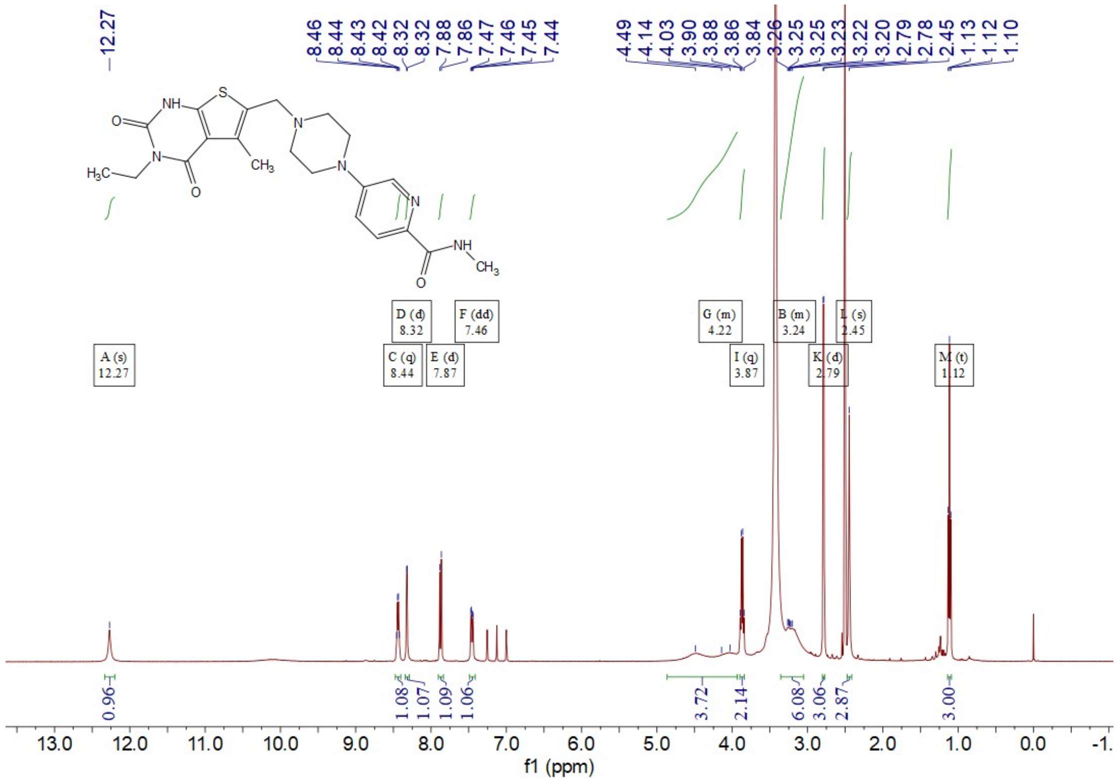

^{13}C NMR Spectrum of **Y4**

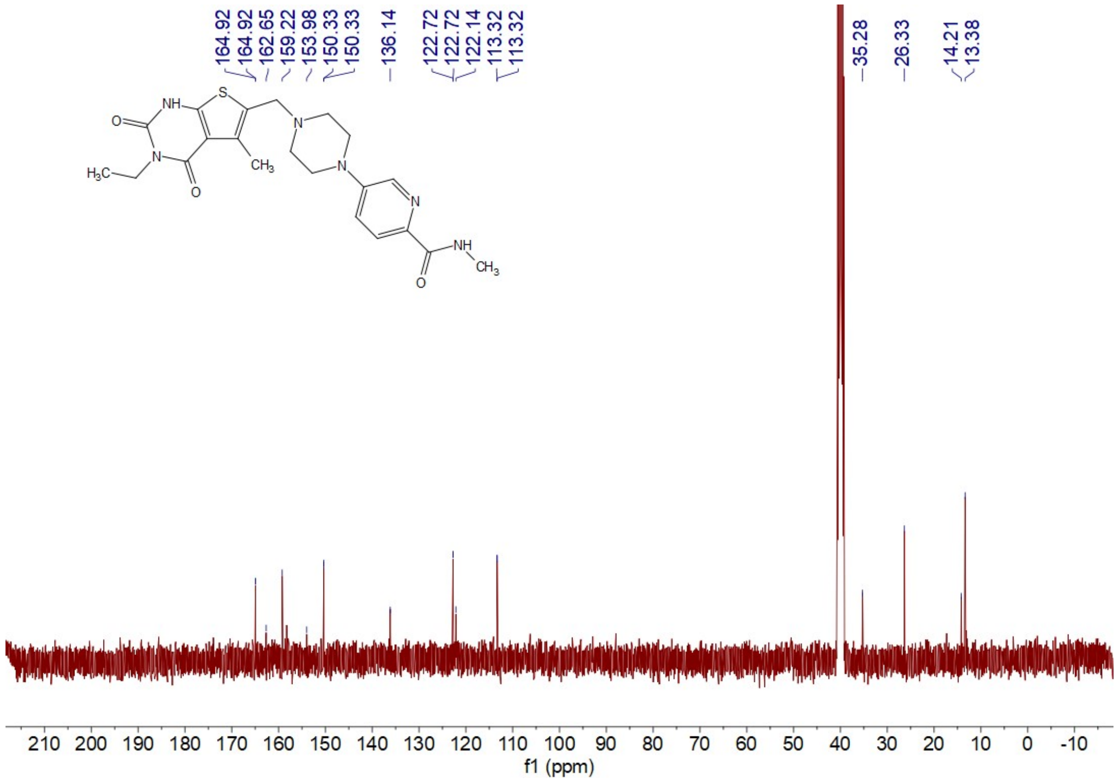

^{1}H NMR Spectrum of **Y5**

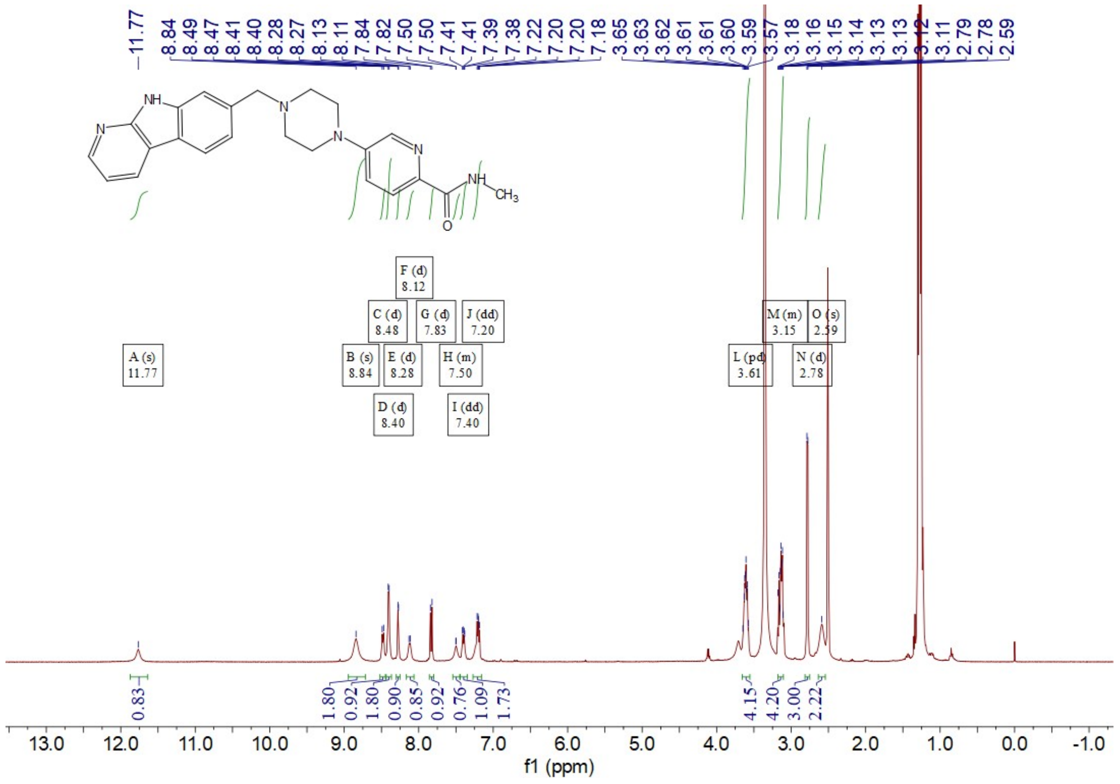

^{13}C NMR Spectrum of **Y5**

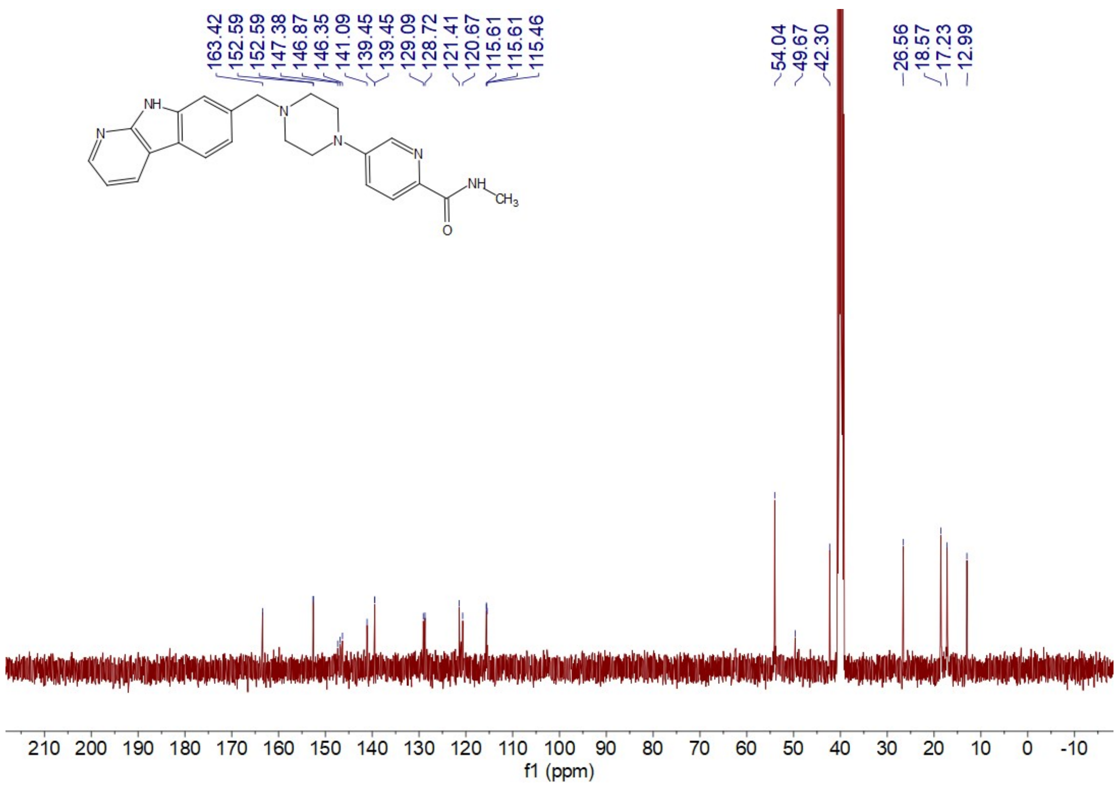

^{1}H NMR Spectrum of **Y6**

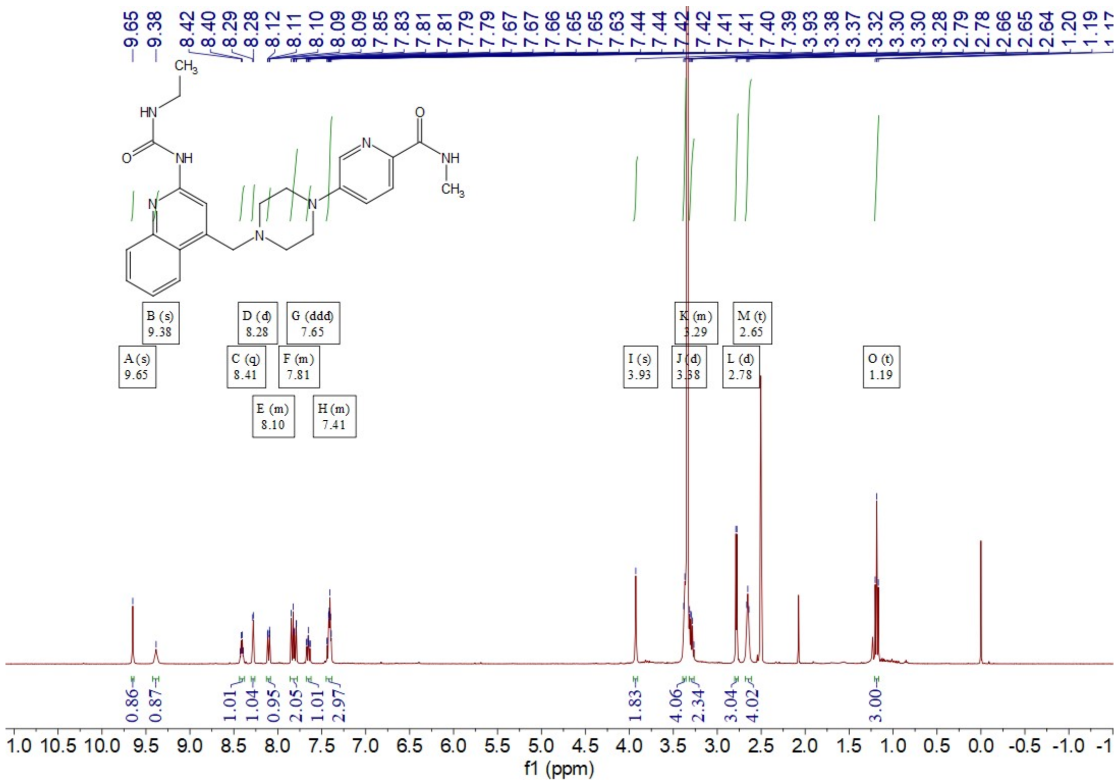

^{13}C NMR Spectrum of **Y6**

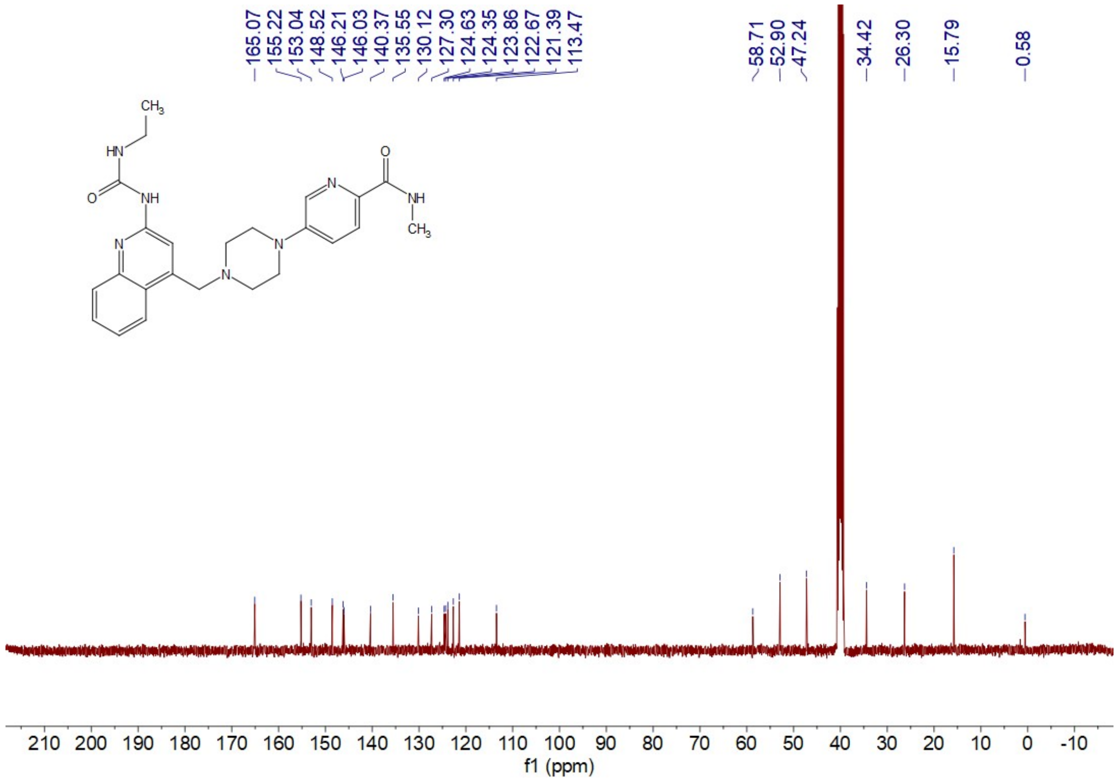

^{1}H NMR Spectrum of **R1**

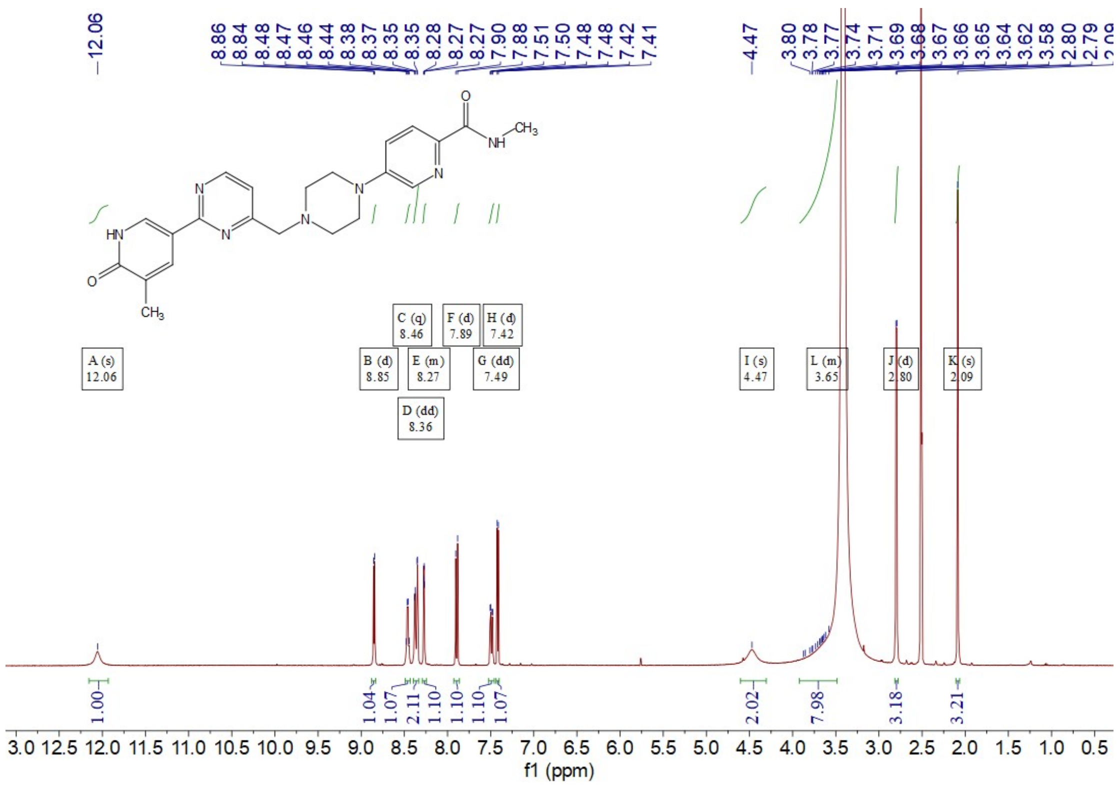

^{13}C NMR Spectrum of **R1**

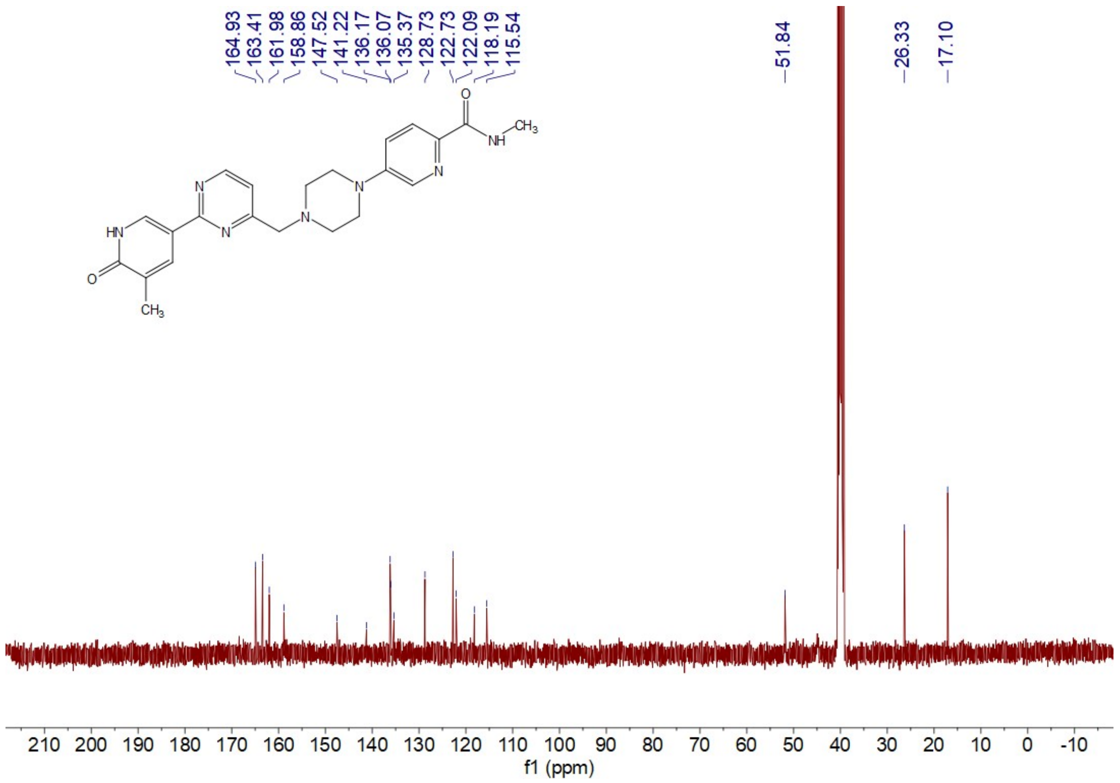

^{1}H NMR Spectrum of **R2**

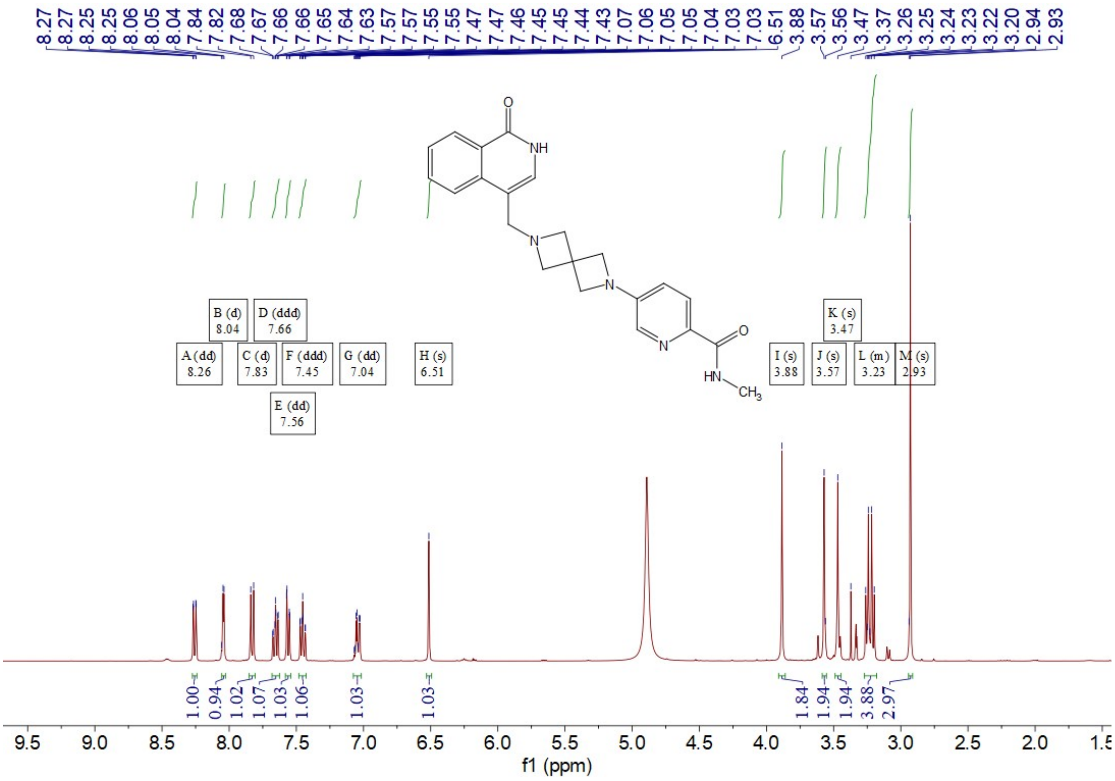

^{13}C NMR Spectrum of **R2**

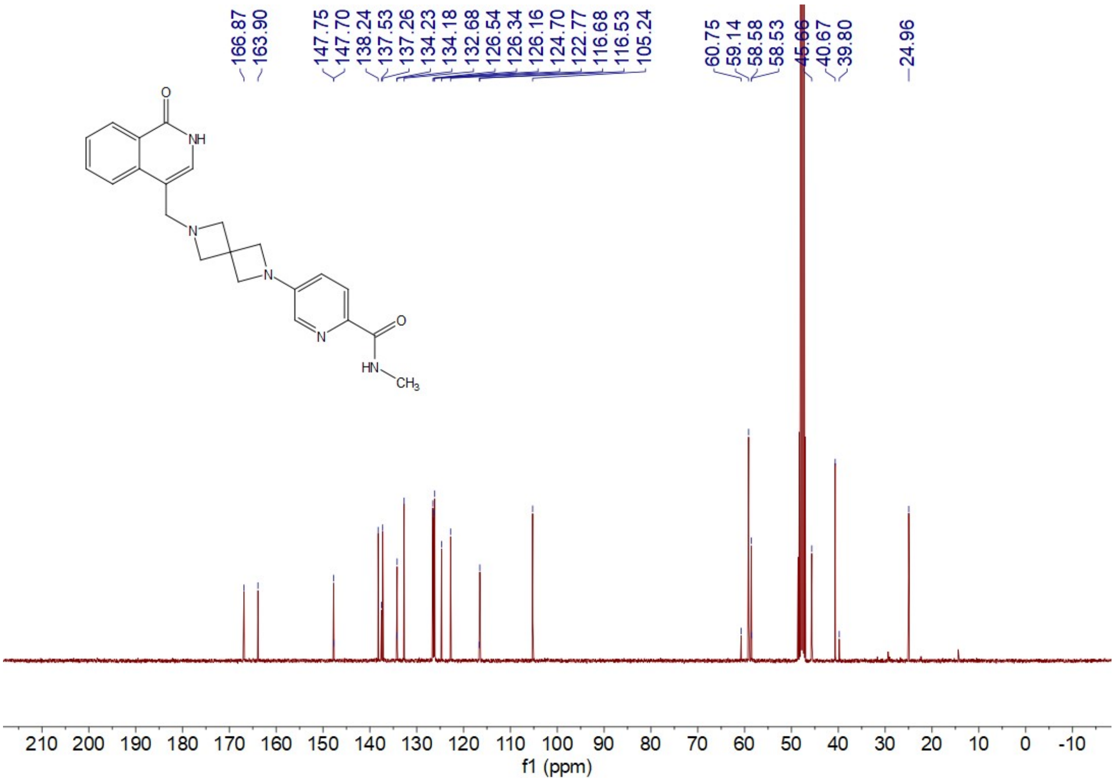

References

1. Diederik P. Kingma, J.B. Adam: A Method for Stochastic Optimization. *arXiv*, 1412.6980 (2017).
2. Ragoza, M., Masuda, T. & Koes, D.R. Generating 3D molecules conditional on receptor binding sites with deep generative models. *Chem. Sci.* **13**, 2701-2713 (2022).
3. Meng Liu, Y.L., Kanji Uchino, Koji Maruhashi, Shuiwang Ji. Generating 3D Molecules for Target Protein Binding. *arXiv*, 2204.09410 (2022).
4. Peng, X.*, et al.* Pocket2Mol: Efficient Molecular Sampling Based on 3D Protein Pockets. in *39th International Conference on Machine Learning, ICML 2022*, Vol. 162 17644-17655 (2022).
5. Arne Schneuing, Y.D., Charles Harris, Arian Jamasb, Ilia Igashov, Weitao Du, Tom Blundell, Pietro Lió, Carla Gomes, Max Welling, Michael Bronstein, Bruno Correia. Structure-based Drug Design with Equivariant Diffusion Models. *arXiv*, 2210.13695 (2023).
6. Zhang, O.*, et al.* ResGen is a pocket-aware 3D molecular generation model based on parallel multiscale modelling. *Nat. Mach. Intell.* **5**, 1020-1030 (2023).
7. Zhang, O.*, et al.* Learning on topological surface and geometric structure for 3D molecular generation. *Nat. Comput. Sci.* **3**, 849-859 (2023).
8. Sun, H.*, et al.* Assessing the performance of MM/PBSA and MM/GBSA methods. 7. Entropy effects on the performance of end-point binding free energy calculation approaches. *Physical Chemistry Chemical Physics* **20**, 14450-14460 (2018).
